# Estimating reproductive costs in marine mammal bioenergetic models: a review of current knowledge and data availability

**DOI:** 10.1093/conphys/coac080

**Published:** 2023-01-18

**Authors:** Elizabeth A McHuron, Stephanie Adamczak, Daniel P Costa, Cormac Booth

**Affiliations:** Cooperative Institute for Climate, Ocean, and Ecosystem Studies, University of Washington, Seattle, WA, 98105, USA; Ecology and Evolutionary Biology Department, University of California Santa Cruz, Santa Cruz, CA, 95064, USA; Ecology and Evolutionary Biology Department, University of California Santa Cruz, Santa Cruz, CA, 95064, USA; SMRU Consulting, Scottish Oceans Institute, St Andrews, UK

## Abstract

Reproductive costs represent a significant proportion of a mammalian female's energy budget. Estimates of reproductive costs are needed for understanding how alterations to energy budgets, such as those from environmental variation or human activities, impact maternal body condition, vital rates and population dynamics. Such questions are increasingly important for marine mammals, as many populations are faced with rapidly changing and increasingly disturbed environments. Here we review the different energetic costs that marine mammals incur during gestation and lactation and how those costs are typically estimated in bioenergetic models. We compiled data availability on key model parameters for each species across all six marine mammal taxonomic groups (mysticetes, odontocetes, pinnipeds, sirenians, mustelids and ursids). Pinnipeds were the best-represented group regarding data availability, including estimates of milk intake, milk composition, lactation duration, birth mass, body composition at birth and growth. There were still considerable data gaps, particularly for polar species, and good data were only available across all parameters in 45% of pinniped species. Cetaceans and sirenians were comparatively data-poor, with some species having little or no data for any parameters, particularly beaked whales. Even for species with moderate data coverage, many parameter estimates were tentative or based on indirect approaches, necessitating reevaluation of these estimates. We discuss mechanisms and factors that affect maternal energy investment or prey requirements during reproduction, such as prey supplementation by offspring, metabolic compensation, environmental conditions and maternal characteristics. Filling the existing data gaps highlighted in this review, particularly for parameters that are influential on bioenergetic model outputs, will help refine reproductive costs estimated from bioenergetic models and better address how and when energy imbalances are likely to affect marine mammal populations.

## Teaser

Estimates of how much energy a female needs to reproduce successfully are required to understand the impact of environmental perturbations on marine mammal populations. We review the state of knowledge and data availability on reproductive energetics, highlighting key data gaps to help refine future estimates from bioenergetic models.

## Introduction

Bioenergetic models provide valuable insight into conservation concerns facing many marine mammal populations, such as climate change, prey availability and disturbance from human activities (e.g. Villegas-Amtmann *et al.,* 2015; Reimer *et al.,* 2019; Silva *et al.,* 2020; Gallagher *et al.,* 2021). They also have broader ecosystem applications since results can be used to inform the integration of marine mammals into ecosystem-based fisheries management (Chasco *et al.,* 2017; Goedegebuure *et al.,* 2017; McHuron *et al.,* 2020). Modeling approaches range from static “accounting” models, where the primary goal is often to estimate individual- and population-level prey consumption, to more dynamic approaches that simulate the allocation of energy to different processes (e.g. metabolism, growth, reproduction) to examine population-level processes, often within an individual-based modeling framework (Pirotta, 2022).

Reproductive females are a focal group of many bioenergetic approaches. This is largely because survival of reproductive females and reproductive rates are key drivers of marine mammal population dynamics (Blázquez *et al.,* 2020; Fruet *et al.,* 2021; Luck *et al.,* 2022). Reproduction is also energetically expensive, meaning that females (and their offspring) may be more susceptible to perturbations in energy balance during this time. An inability to obtain sufficient prey or energy reserves may result in abortion or changes in lactation duration (e.g. Trillmich and Limberger, 1985; Pitcher *et al.,* 1998; Wasser *et al.,* 2017), skipped reproductive events (e.g. Marsh and Kwan, 2008; Gailey *et al.,* 2020), or in extreme cases, female mortality (Chinn *et al.,* 2016). For example, end-lactation syndrome, mortality due to depletion of energy reserves during lactation, has been observed more frequently in southern sea otters (*Enhydra lutris*) from resource limited populations compared with nonresource limited ones (Chinn *et al.,* 2016). In addition, females often comprise a large proportion of a population’s total prey consumption, in part due to the high cost of lactation (McHuron *et al.,* 2020), making them an important group to model in terms of their potential impacts on food web dynamics.

Accurate characterization of the role of reproductive females in food webs and the impacts of natural and anthropogenic stressors on this demographic group requires an understanding of the energetic costs of reproduction at various temporal scales. The reproductive energetics of pinnipeds has been extensively studied, albeit in a limited number of species. Their ease of handling and ability to house them in human care has led to comprehensive studies on energy expenditure, mass dynamics, and milk composition and intake (see *Gestation* and *Lactation* below). Knowledge on reproductive energetics for the less specious marine mammal groups (ursids, sirenians, and mustelids) is variable, ranging from little knowledge in sirenians to more extensive knowledge in polar bears (*Ursus maritimus*; Derocher *et al.,* 1993; Arnould and Ramsay, 1994; Hedberg *et al.,* 2011). Comparatively little is known of the reproductive energetics of cetaceans, the most specious group of marine mammals. This is mostly due to their large body size and fully aquatic nature, since many studies on reproductive energetics require some form of animal handling. While there are numerous cetacean bioenergetic models that have estimated the costs of reproduction, it has been some time since there has been a review of cetacean reproductive energetics or the parameters that influence the cost of reproduction (Perrin and Reilly, 1984; Oftedal, 1997). There is a need to revisit available data given the accumulation of data from new studies, many of which use recent technological advancements to collect data from free-ranging animals, and the recent identification of lactation energetics as a key unanswered question for bioenergetic applications to marine mammal management and conservation (McHuron *et al.,* 2022a).

Here we provide a review of the current state of knowledge on the reproductive energetics of marine mammals, focusing on the specific costs of gestation and lactation and how these costs are estimated in traditional bioenergetic models (i.e. not dynamic energy budget [DEB] models). We also review data on food consumption during reproduction because, although it is the manifestation of the cumulative sum of an individual's current (and potentially future) energy needs, food consumption can provide useful information about reproductive costs and the existence of potential energetic compensatory mechanisms. A primary goal of this review was to compile existing data on the parameters used to estimate reproductive costs in marine mammals to not only highlight data gaps, but also provide a useful resource for researchers in parameterizing bioenergetic models. While we covered all taxonomic groups, we focused particular effort toward cetaceans given previous reviews on pinnipeds (Lavigne *et al.,* 1982; Oftedal *et al.,* 1987a; Costa, 1991; Costa and Valenzuala-Toro, 2021; Costa and Maresh, 2022) and the clear need to better understand the reproductive energetics of cetaceans.

## Data Availability

To compile data availability on common parameters used in marine mammal bioenergetic models, we performed literature searches using Google Scholar and keywords relevant to each parameter and species. We also searched review articles on reproductive energetics and common marine mammal reference material (e.g. Encyclopedia for Marine Mammals) for values and their original sources. We traced the literature back to the source where the original estimate was obtained; in many cases, this proved to be a highly circuitous route as many papers cited other papers that were also not original sources. It was not always possible to obtain a copy of what was assumed to be the original source; when this occurred, we note this in the supplemental tables and provide both the citing research and source citation. We recommend caution in applying these values without accessing the original data sources. The exception to this was when the citation was to a known review, such as a book chapter, because in our experience these often yielded uncited values or reference to other sources. To avoid perpetuating erroneous or poorly informed parameters and overstating data availability, we did not include parameter values for which no citation was given. We also did not include every single reference for a given parameter and species, as this was not the goal of the review. While this effort was thorough, there is a vast amount of gray literature on marine mammals, and we recognize that it is possible that we missed data. We used the Society for Marine Mammalogy’s Taxonomy List of Marine Mammal Species and Subspecies, updated in June 2021, for species classification (https://marinemammalscience.org/science-and-publications/list-marine-mammal-species-subspecies/). On this list there were 15 extant species of mysticetes, 77 odontocetes (with one included as possibly extinct), 18 phocids, 14 otariids, 1 odobenid, 2 mustelids, 4 sirenians and 1 ursid.

## Gestation

Energetic costs during gestation are incurred because the female must invest energy in creating and maintaining the fetus and other tissues associated with pregnancy. Physiological changes during pregnancy are not well documented for marine mammals, but in other mammals include changes in the circulatory (e.g. increased blood volume, changes in cell counts and platelet production), musculoskeletal (e.g. increased bone turnover) and renal systems (Soma-Pillay *et al.,* 2016; Torres *et al.,* 2018), in addition to the necessary changes to the reproductive system (e.g. development of the placenta, mammary tissue). The metabolic cost of pregnancy is referred to as the heat increment of gestation (HIG), defined as the amount of extra heat produced during gestation above nongestation levels. The gestation costs discussed in this section, the HIG and the energy stored in the fetus and associated tissues, do not include the energy needed by the female for her own metabolic needs (outside of the HIG), as while they are incurred during pregnancy, they are not a cost of gestation per se.

Pinnipeds, polar bears and sea otters exhibit embryonic diapause (delayed implantation) that occurs following conception, which considerably slows embryonic growth (Daniel, 1971). Diapause has not yet been documented in marine otters (*Lontra felina*) but is likely given its prevalence in other mustelids, including the closely related North American river otter (*L. canadensis*) and sea otter (Sinha *et al.,* 1966; Thom *et al.,* 2004). Diapause lasts anywhere from 1.5 to 8 months, depending on species (Sinha *et al.,* 1966; Boyd, 1991; Atkinson, 1997). Most bioenergetic models do not consider this time of “nonactive” gestation when estimating energy budgets since any costs are likely negligible.

The most common approach for estimating the HIG in marine mammal bioenergetic models is the formula of Brody, $(kcals)=4400\bullet {M}^{1.2}$, where $M$ is the mass at birth in kg (Brody, 1938). This equation was derived using data from domesticated animals (rats, chickens, rabbits, pigs, cows, goats and horses) and humans, and represents the total HIG. Despite its widespread use, Brody (1938) noted that the numerical constants of his equation were tentative and subject to further revision given the limited number of individuals the equation was derived from (all but one species had data from one to six individuals). To our knowledge, such a revision was never undertaken. Other equations, as noted by Yunker et al. (2005), include those of Moen (1973) for ruminants and Robbins (1983) for ungulates. We do not provide any discussion or elaboration of these two equations given they are rarely used in the marine mammal literature, and we were unable to access the original sources. One alternate approach that has been used to estimate the HIG includes incorporating estimates of fetal mass into maternal mass, which is then used to estimate maternal metabolic demands (Hin *et al.,* 2019).

Body mass at birth is the only parameter required to estimate the HIG from Brody's equation. Estimates of body mass at birth (or length that can be converted to body mass) exist for 100% (odobenids, otariids, ursids), 94% (phocids), 75% (sirenians), 66% (mysticetes), 64% (odontocetes) and 50% (mustelids) of marine mammal species (Supplementary Tables S1-S5). Since the resulting value represents costs across the entire gestation period, many studies convert this to a daily cost using fetal growth curves (Pirotta *et al.,* 2018; McHuron *et al.,* 2021) or gestation duration (Gallagher *et al.,* 2018), the latter of which assumes linear increases in the HIG. Fetal growth curves are available for 100% (odobenids), 53% (mysticetes), 39% (phocids), 18% (odontocetes), 14% (otariids) and 0% (sirenians, mustelids, ursids) of marine mammal species (Supplementary Tables S6-S10). Increases in fetal size with age typically follows an exponential pattern (e.g. Trites, 1991; Robeck *et al.,* 2015; Lanzetti *et al.,* 2020), resulting in relatively small energetic costs throughout much of pregnancy, only increasing in the later stages of gestation. For example, estimated gestation costs for southern right whales (*Eubalaena australis*) were 0 MJ day^−1^ at conception and 41 MJ day^−1^ at the end of second trimester, but increased to 725 MJ day^−1^ by the end of gestation (Christiansen *et al.,* 2022b).

The energy stored in fetal tissue is typically calculated based on mass at birth and estimates of the chemical composition of the fetus. As above, these costs can be extrapolated to daily costs using fetal growth curves to calculate the amount of new mass added daily. In cetaceans, fat deposition increases as gestation progresses, both in terms of relative blubber deposition and the lipid content of blubber and muscle (Lockyer, 1993; Borrell *et al.,* 1995; Struntz *et al.,* 2004). For example, the percent blubber content of long-finned pilot whale (*Globicephala melas*) fetuses increased from zero during early gestation to an estimated 71% at birth (Borrell *et al.,* 1995). Bottlenose dolphin (*Tursiops truncatus*) fetuses close to parturition had one of the thickest blubber layers (relative to body size) across all life history categories, which may help newborn dolphins thermoregulate and maintain buoyancy (Struntz *et al.,* 2004). The energetic content of harp seal (*Pagophilus groenlandica*) pups increased rapidly in the last two months of gestation (Stewart *et al.,* 1989), indicating that pinnipeds also exhibit increases in fat deposition toward the end of gestation despite additional thermoregulatory tissues (fur) and their semi-aquatic nature. In support of this, the lipid content in blubber of ringed seal (*Pusa hispida*) and California sea lion (*Zalophus californianus*) fetuses increased with fetal age (Greig *et al.,* 2007; Brown *et al.,* 2016). Similar patterns in fat deposition have been detected in some other mammals, such as guinea pigs, pigs and humans (Engle and Lemons, 1986; McPherson *et al.,* 2004; Toro-Ramos *et al.,* 2015).

Since there are very few estimates of fetal or neonatal body composition for marine mammals, most bioenergetic models do not account for the change in body composition during fetal growth, instead using body composition at birth (or near birth) to estimate energy storage. Estimates of fat and protein content of full-term fetuses or newborn phocid pups range from 3.0% to 14.0% and from 18.6% to 23.1%, respectively (Supplementary Table S11). Newborn or late-term cetacean fetuses are comprised of 22.0% to 43.4% blubber, 13.1% to 17.6% muscle and 12.0% to 14.5% viscera, although these ranges are based on a very limited number of species and individuals (Supplementary Table S11). Lipid composition of blubber ranges from 42.3% to 79.2% (Gauthier *et al.,* 1998; Struntz *et al.,* 2004; Dunkin *et al.,* 2005; Pedro *et al.,* 2017).

The inclusion of energy storage in other pregnancy-related tissues in bioenergetic models is variable, with some including these costs and others omitting it. Data from pinnipeds indicate that the vast majority (90–95%) of energy storage during gestation is in the fetus itself (Anderson and Fedak, 1987; Lydersen, 1995). Lavigne and Stewart (1979) noted that the energy density of harp seal placenta was 1.13 kcal g^−1^ (4.73 MJ kg^−1^) wet weight which, when combined with the mass of the placenta, resulted in a total energy content of 1430 kcal (5.98 MJ), roughly 5.8% of the energy stored in the fetus. The energy density of ringed seal placenta was similar at 3.75 MJ kg^−1^, for a total cost of 1.3 MJ (Lydersen, 1995). Boyd (1990) and Yunker et al. (2005) both provide equations for the relationship between placental mass and fetal mass, although they are for a single species (gray seals *Halichoerus grypus*), and it is unknown whether this relationship would hold outside the mass range of their study.

Gestation may affect energy demands through other pathways, but these costs have not been well quantified for marine mammals. For example, pregnant bottlenose dolphins experienced increased drag due to changes in body morphology that affected frontal surface area (Noren *et al.,* 2011). Using fluid dynamics modeling, Nousek McGregor (2010) predicted that pregnant North Atlantic right whales (*Eubalaena glacialis*) have increased locomotor costs due to a 3% to 4% increase in drag caused by changes in body morphology. In deep-diving northern elephant seals (*Mirounga angustirostris*), pregnant females exhibited shorter dive durations after the 3^rd^ month of pregnancy, potentially because of increased fetal oxygen needs. Such changes in diving behavior have the potential to affect energy balance since they limit the amount of time seals can spend at depth foraging (Hückstädt *et al.,* 2018).

## Lactation

Total and daily costs of lactation vary drastically among marine mammals. Energetic investment in offspring is driven in part by differences in body size, which ranges from < 5 kg for a female marine otter (Soto-Azat *et al.,* 2011) to upward of 150 000 kg for an adult female blue whale (*Balaenoptera musculus*, Lockyer, 1976). Other factors, such as lactation duration and lactation strategy (i.e. where the animal falls on the capital-income spectrum), also drive interspecific variation in lactation costs. For example, costs of lactation in hooded seals (*Cystophora cristata*), which have the shortest lactation duration of any mammal at four days, were on average 756 MJ or 14 MJ day^−1^ kg^-0.83^ (Oftedal *et al.,* 1993). This contrasts with otariid seals, such as Antarctic fur seals (*Arctocephalus gazella*) where total lactation costs were 813 to 1064 MJ or about 1.6 MJ day^−1^ kg^-0.83^ across the roughly 4-month lactation period (Arnould, 1997). Delivering high daily amounts of energy to offspring is more efficient than less energy delivered over a prolonged time because a female has to cover the metabolic overhead of her offspring throughout lactation (Boness and Bowen, 1996; Costa and Maresh, 2022). Many species do not, however, have the reserve capacity to achieve such high rates of energy transfer.

The cost of lactation is largely comprised of the energy contained within the milk itself. A female may also expend metabolic energy to produce the milk, but these costs appear to be negligible in many marine mammals. Using doubly labeled water (DLW), Costa and Trillmich (1988) and Costa and Gentry (1986) found no difference in onshore field metabolic rates of lactating and nonlactating Antarctic and northern fur seals (*Callorhinus ursinus*), suggesting negligible costs associated with milk production. While it is possible that any increased costs were obscured by measurement error or because milk production costs were incurred outside the measurement interval (i.e. at sea), similar results were found between resting metabolic rates of lactating and nonlactating female California sea lions in human care (Williams *et al.,* 2007). In contrast, sea otters do exhibit a metabolic cost of milk production, as evidenced by increases in resting metabolic rate above nonreproductive levels in the second month following parturition that remained elevated throughout lactation (Thometz *et al.,* 2016a). Disparities between sea otters and pinnipeds may be due to an increased need by sea otters for *de novo* synthesis of fatty acids and because of their limited capacity to store energy reserves, since the fatty acids present in blood lipids of fasting seals are nearly identical to those in milk (Riedman and Ortiz, 1979; Costa and Trillmich, 1988; Thometz *et al.,* 2016a). As with gestation, we do not include further discussion of the female's metabolic overhead during lactation since it is not a cost of lactation per se (but see *Maternal food intake during gestation and lactation*).

The cost of lactation depends on the energy density of milk (determined by milk composition), the rate of milk consumption by offspring, and the duration of lactation. There are a variety of ways that lactation costs are estimated in bioenergetic models, largely due to data availability and model complexity. Some studies take a ‘top-down’ approach, where lactation costs are estimated based on the female’s output, such as milk output (or milk intake by offspring), body mass changes (only in capital breeders), or differences in food intake between reproductive and nonreproductive animals (e.g. Christiansen *et al.,* 2016; Bejarano *et al.,* 2017; Gallagher *et al.,* 2018; McHuron *et al.,* 2020). The alternate ‘bottom-up’ approach sums the costs experienced by offspring to determine maternal investment, such as metabolic rates, growth, and waste production (e.g. Lockyer, 1981; Fortune *et al.,* 2013; Villegas-Amtmann *et al.,* 2015). In the following sections, we provide an overview of the data availability and general patterns for the key parameters used to estimate lactation costs in bioenergetic models. We do not review offspring metabolic rates; this is a large topic unto itself, and metabolic rates are covered in a separate review in this special issue (Noren in review). Additional discussion of many of the parameters described below can be found in various reviews by Olav Oftedal (Oftedal *et al.,* 1987a; Oftedal, 1993; Oftedal *et al.,* 1996; Oftedal, 1997, 2000).

### Milk composition and energy density

**Figure 1:**
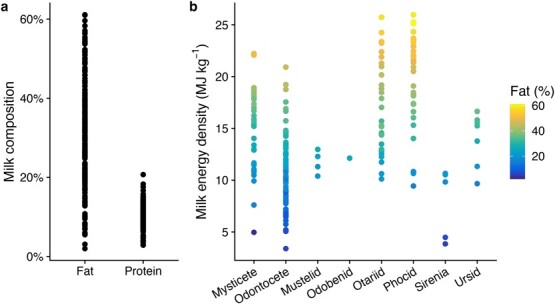
Fat and protein composition (a) and energy density (b) of marine mammal milk. In b, milk energy density was calculated using fat and protein compositions and conversion factors of 39.3 MJ kg^−1^ (fat) and 24.5 MJ kg^−1^ (protein). See Supplementary Tables S12-S15 for values and sources.

Proximate composition of milk has been determined in 100% (otariids, odobenids, ursids), 55% (phocids), 53% (mysticetes), 50% (mustelids), 26% (odontocetes) and 25% (sirenians) of species (Supplementary Tables S12-S15). Fat composition of marine mammal milk is extremely variable, ranging from < 10% to nearly 60% (Fig. 1, Supplementary Tables S12–S15). While phocids have the highest mean milk fat content of any marine mammal, the range of values almost entirely overlaps with that of otariids. The average milk fat composition of females otariids with newborn pups (28.2–38.6%) is within the range observed for phocids (16.1–40.8%), excluding hooded seals that have milk fat contents above 50% (Oftedal *et al.,* 1988; Werner, 1996; Arnould and Hindell, 1999; Mellish *et al.,* 1999b; Georges *et al.,* 2001). It is difficult to draw comparisons for most other groups given small samples sizes and the general lack of good temporal coverage in sample collection, which has a considerable effect on milk fat composition (e.g. Derocher *et al.,* 1993; Mellish *et al.,* 1999a; Donohue *et al.,* 2002; Lang *et al.,* 2005; West *et al.,* 2007). In otariids, milk composition changes not only across the lactation interval but also across a nursing visit (Costa and Gentry, 1986; Georges *et al.,* 2001). In the only cetacean study to examine temporal variation, West et al. (2007) found that milk fat estimates from bottlenose dolphins never exceeded 25.2%, suggesting that at least some odontocetes have lower milk fat compositions than pinnipeds and mysticetes. Milk protein composition is much less variable, with most values between 8% and 11% across all marine mammal taxonomic groups.

**Figure 2:**
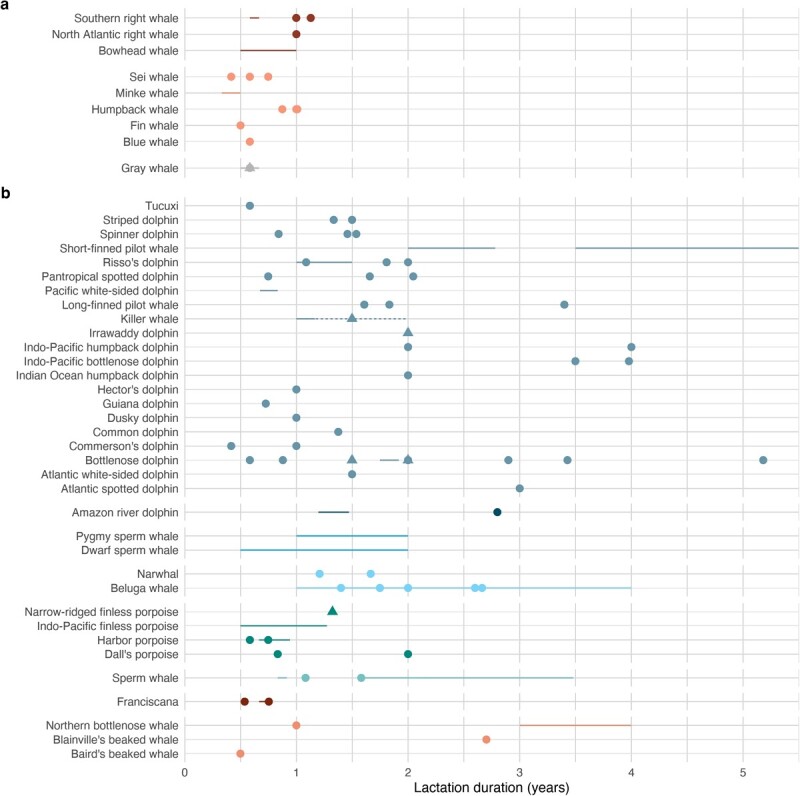
Mean lactation durations of mysticetes (a) and odontocetes (b), ordered and colored by family. Point estimates represent means from individual studies, with shapes indicating whether data were collected from animals in the wild (circle) or human care (triangle). Line ranges are provided when a range of values was given instead of a single mean, with dashed lines indicating data collected from animals in human care. See Supplementary Tables S17 and S18 for values and sources.

Milk energy density can be measured directly using bomb calorimetry or calculated based on the proximate composition of milk and the energy density of protein and fat. Other components of milk, such as carbohydrates, are often not included in energy density estimates since they comprise a relatively small proportion (often < 1%) of marine mammal milk and thus are not always measured (Oftedal, 1993; West *et al.,* 2007; Eisert *et al.,* 2013). Energy density values derived using proximate composition are highly correlated with those from bomb calorimetry (Oftedal *et al.,* 2014). Based on conversion factors (39.3 MJ kg^−1^ for fat and 24.5 MJ kg^−1^ for protein), energy densities of marine mammal milk typically range from 3.4 to 26.0 MJ kg^−1^ (Fig. 1). Energy density of individual samples may in some cases exceed this range, as we primarily used study averages to calculate these values. Since protein composition is broadly similar across all marine mammals, variation in milk energy density is primarily driven by fat composition.

### Milk intake rates

Milk intake has been measured in 100% (ursids), 43% (otariids) and 39% (phocids) of species using labeled water (Supplementary Table S16). Zhang et al. (2014) report milk intake in a captive spotted seal (*Phoca largha*) fed expressed milk, bringing the total for phocids to 44%. Milk intake rates vary from an average of 0.23 to 0.68 kg day^−1^ in polar bears, 0.35 (reported in mL day^−1^) to 2.1 kg day^−1^ in otariids, and 1.2 to 10.4 kg day^−1^ in phocids. Pinniped milk intake rates (kg day^−1^ and MJ day^−1^) typically increase as the pup grows, although the relationship between milk intake rates and body mass may break down at different pup developmental states (Oftedal *et al.,* 1987b; McDonald *et al.,* 2012). Milk intake rates do not always increase linearly until the end of lactation, at least in otariids where weaning is a gradual process. For example, Antarctic and Australian fur seals (*Arctocephalus pusillus doriferus*) exhibited peak milk intake rates at roughly 70% and 67% of the lactation interval, respectively (Arnould *et al.,* 1996; Arnould and Hindell, 2002). It is unclear if this is a general pattern for otariids, since northern fur seal pups exhibited increased milk intake rates until at least 95 days of age, roughly 70% to 80% of the lactation interval (Donohue *et al.,* 2002). Sex differences in milk intake rates have been detected in some studies (Costa and Gentry, 1986; Oftedal *et al.,* 1987b) but not others (Kretzmann *et al.,* 1993; Lunn and Arnould, 1997; Arnould and Hindell, 2002; Donohue *et al.,* 2002). Across 2 years, Donohue et al. (2002) found that female northern fur seal pups consumed less milk per day in one year but not the other. When present, such differences appear to be largely driven by differences in body mass and not because of differential maternal investment. Male pups may be heavier than female ones because of how they invest that energy in growth, with female pups accumulating more fat mass and male pups more lean tissue mass (Arnould *et al.,* 1996; Arnould and Hindell, 2002).

There are no empirical measurements of milk intake for cetaceans, mustelids or sirenians. Estimates of cetacean milk output are provided in Table 9 of Oftedal (1997); however, these values were derived using either (1) estimates of calf growth rates and the assumed relationship between kg of milk ingested per kg of body mass gained, or (2) assumptions about milk production based on the mass of mammary tissue. Assumptions about milk ingestion to growth and milk production based on mammary tissue mass were both derived from phocid seals. Resulting estimates of milk output ranged from 22 to 220 kg day^−1^ (330–4000 MJ day^−1^) in mysticetes and from 0.42 to 9.0 kg day^−1^ (6.0–110 MJ day^−1^) in odontocetes. Sumich (1986) estimated gray whale calves required 17.9 kg day^−1^ of milk (1430 kg of milk across an 80 day stay in a breeding lagoon) based on a bioenergetic model of calf energy needs, a value that is considerably different than the 60 to 150 kg day^−1^ estimated by Oftedal (1997) from mammary gland mass. Differences in predicted milk outputs may be due to the reliance on phocid data to estimate the amount of milk produced by each gram of mammary tissue. Because of the lack of information on milk output/intake, most recent bioenergetic models on species from these groups have used other approaches to estimate lactation costs.

### Lactation duration

Lactation durations have been estimated for 100% (ursids, odobenids, mustelids, otariids), 89% (phocids), 60% (mysticetes), 45% (odontocetes) and 25% (sirenians) of marine mammal species (Fig. 2; Supplementary Tables S17–S21). There are estimates in the literature for two additional mysticetes (Bryde's *Balaenoptera edeni* and pygmy right whales *Caperea marginata*), but these estimates are speculative at best (Ross *et al.,* 1975; Best, 1977) and were not included in the above percentages. In compiling lactation durations, we also generally excluded literature that did not explicitly provide a formal estimate of lactation duration, such as when anecdotal observations of weaning time were reported (e.g. Clark and Odell, 1999). The abundance of lactation duration data for cetaceans is somewhat misleading. Many of these estimates are not based on actual observations but assumed from other approaches, such as the presence of milk (or prey) in stomach contents of young animals, or the relative occurrence of different reproductive stages in harvested animals. In some cases, this can result in lactation durations that are not well resolved, particularly if the composition of fisheries catches is nonrandom (Kasuya, 1978). Even when direct observations are available, they are often based on associations between an adult female and her presumed calf, which could result in erroneous estimates if they remain associated for reasons other than nursing, are not always together (a common occurrence), or are in fact not a mother-calf pair (Hamilton *et al.,* 2022).

Lactation durations of marine mammals vary greatly among species, from a minimum of 4 days in the hooded seal to an average of 3 to 4 years in some odontocetes. Within pinnipeds, phocids have much shorter lactation durations (4 days to 5 months) than otariids (4 months to several years), which is consistent with observations that phocids have a greater reliance on energy reserves (capital) stored prior to the breeding season than otariids that support costs by foraging during lactation (income). Reviews of lactation durations of pinnipeds and analysis of the factors that may have led to differing lactation strategies between phocids and otariids can be found elsewhere (Schulz and Bowen, 2004, 2005; Ferguson, 2006; Costa and Valenzuala-Toro, 2021; Costa and Maresh, 2022). Mysticetes, which are capital breeders (or mostly), tend to have shorter lactation durations than odontocetes, with average durations of 1 year or less. In contrast, odontocetes typically nurse their calves for 1.5 to 4 years, with maximum durations up to 13+ years. Porpoises and some river dolphins are exceptions that nurse their calves for less than one year on average (Supplementary Table S18), although there are conflicting estimates for Dall's porpoise *(Phocoenoides dalli)* that make it unclear whether they follow this same pattern (Kasuya, 1978; Newby, 1982).

New data on lactation durations continue to refine earlier estimates. For example, a recent study by Feyrer et al. (2020) using stable isotope analysis of teeth showed that lactation durations of northern bottlenose whales (*Hyperoodon ampullatus*) were 3 to 4 years instead of the previous best estimate of at least 1 year, which was a minimum estimate based on the presence of milk and prey in the stomach of a one-year old calf (Benjaminsen and Christensen, 1979). Satellite telemetry data from a single leopard seal (*Hydrurga leptonyx*), a species where lactation durations are unconfirmed but speculations range from 10 days to 8 weeks (Southwell *et al.,* 2003), provides preliminary support for a 2-week lactation duration (Kienle *et al.,* 2022). In North Atlantic right whales, the use of genetic identification revealed that at least two calves never observed on the foraging grounds with their mothers survived, indicating successful weaning as early as 7.5 to 8.0 months of age on the northbound migration (Hamilton *et al.,* 2022).

A particular challenge in estimating total lactation costs is that there is a high degree of intraspecific variation in lactation durations in some species. Bioenergetic models and associated outputs, such as dynamic models estimating reproductive parameters, can be sensitive to lactation durations (New *et al.,* 2013). Variation is minimal in phocids (on the order of days) given short lactation durations, but even small absolute fluctuations can have significant impacts on individual lactation costs (Mellish *et al.,* 1999a). Otariids can extend lactation durations by months or even years, typically in response to environmental conditions; such extensions often come at the expense of the reproductive effort in following years (Pitcher *et al.,* 1998; Trillmich and Wolf, 2008) and can considerably increase the total cost of lactation (McHuron, unpubl. data). Variation in lactation duration in odontocetes is large, both in terms of mean and maximum durations. For example, wild bottlenose dolphins from Ireland had mean lactation durations of 2.9 years (Baker *et al.,* 2018), whereas dolphins in Shark Bay weaned at over 3 years of age (Mann *et al.,* 2000, *Tursiops* sp.), and those in Brazil around the age of 2 years (Fruet *et al.,* 2015). Lactation durations of individuals ranged from 2.0 to 8.59 years (Mann *et al.,* 2000; Baker *et al.,* 2018). Bottlenose dolphin calves managed in human care weaned between 1 and 3 years of age (Peddemors *et al.,* 1992; Reddy *et al.,* 1993; Kastelein *et al.,* 2002). Population differences may largely be driven by resource availability, while within-population differences have been associated with maternal age (Best *et al.,* 1984; Kasuya and Marsh, 1984; Martin and Rothery, 1993; Karniski *et al.,* 2018), body size (Mellish *et al.,* 1999a) and body condition (Cordes and Thompson, 2013). When lactation durations drastically exceed mean durations, it is assumed, although technically unknown, that continued lactation is more about comfort and sociality and that offspring gain little in terms of nutrition.

### Milk efficiency

The digestive efficiency of milk in marine mammals has not been directly measured but is typically assumed to be high given the fat content of milk and measurements of prey digestive efficiency. Ortiz et al. (1984) suggested that it must at least be as high as 90% to 95% in northern elephant seal pups, since predicted mass gain based on milk energy intake was very close to actual mass gain. In sheep, digestive and metabolizable energy efficiencies, which account for fecal and fecal + urinary energy losses, were 98.4% and 95.6% of gross energy intake, respectively (Jagusch and Mitchell, 1971). Proximate composition of sheep milk was 5.74% to 6.76% protein and 5.90% to 8.00% fat, which is considerably different from marine mammal milk that is higher in both fat and protein composition. While higher fat content should increase digestive efficiency, higher protein content could increase urinary energy losses if the protein is catabolized. It is likely, however, that protein in a growing pup or calf is used to create structural tissue (Condit and Ortiz, 1987), so it is unclear how transferable these values are to marine mammals.

### Offspring growth

Growth costs in bioenergetic models are often calculated based on published growth curves and data on the chemical composition of growth (see also Adamczak *et al.* in review). Growth curves (length or mass at age) have been published for 100% (odobenids, ursids, phocids), 79% (otariids), 75% (sirenians), 67% (mysticetes), 51% (odontocetes) and 50% (mustelids) of marine mammal species (Supplementary Tables S6–S10). For cetaceans, dependent calf measurements are largely lacking from growth curves, in large part because many of these curves were generated using harvested animals, a data source biased toward larger individuals. This is changing, however, with the ability to use photogrammetry to estimate body size, in addition to the accumulation of measurements collected from stranded individuals or those sampled as part of long-term research programs (Cheney *et al.,* 2018; Venuto *et al.,* 2020; Fortune *et al.,* 2021). Multiphase growth models indicate that calf growth is rapid in the first few months to a year following parturition (Cockcroft and Ross, 1990; Ferrero and Walker, 1996; Gol’din, 2004; McFee *et al.,* 2010; Agbayani *et al.,* 2020; Fortune *et al.,* 2021), highlighting the need for multiphase growth curves to avoid miscalculations of growth costs. For example, gray whale calves reached one-third of their total expected body mass during their first year (Agbayani *et al.,* 2020), while North Atlantic right whale calves had attained 47% of the mass of a sexually mature animal by 9.6 months (Fortune *et al.,* 2021). The inability to accurately model growth using a single curve has also been noted for pinnipeds (McLaren, 1993). There is evidence from a limited number of species that growth rates and asymptotic lengths may in some cases be population-specific, although this is not well resolved for most species (Galatius and Gol’din, 2011; Baker *et al.,* 2014; Durban *et al.,* 2021).

The energy stored in tissues is typically estimated using either the protein and lipid composition of growth or the allocation of growth to different tissue types (e.g. blubber, muscle, viscera) in conjunction with the lipid and protein composition of each tissue type. Because tissues inherently differ in their energy density, the allocation of energy to different tissue types influences the estimated cost of growth. For example, total estimated growth costs of southern right whale calves varied between 458 000 and 995 000 MJ, depending on the assumed lipid and protein concentration of blubber, muscle, viscera and bone (Christiansen *et al.,* 2022a). Data on growth composition for cetaceans are lacking, but corresponding data from pinnipeds suggests that life-history strategies play a role in energy allocation. Phocid pups typically deposit large amounts of lipid (as blubber) during the relatively short lactation period (Kretzmann *et al.,* 1993; Oftedal *et al.,* 1993), whereas otariid pups appear to prioritize lean tissue growth (and physiological development) across a much longer lactation period (Oftedal *et al.,* 1993; Donohue *et al.,* 2002). Cockcroft and Ross (1990) found a similar pattern of lean tissue prioritization in young bottlenose dolphins, with the ratio of composition diverging from 1:1 when animals exceeded approximately 44 kg in body mass. This does not mean that lipid deposition cannot be significant in otariids, as about 50% of the growth of young-of-the-year Steller sea lions (*Eumetopias jubatus*) aged 5 to 10 months came from lipid deposition (Rehberg *et al.,* 2018). In southern right whale calves, blubber thickness at different sites on the body increased between 31% and 59% from neonates to young calves and 48% to 100% from young to old calves (Marón *et al.,* 2021), although the contribution of lean tissue growth is unknown. Tissue-specific growth may also vary with sex (Arnould *et al.,* 1996; Arnould and Hindell, 2002), but sex-specific growth strategies largely do not appear to impact maternal energy investment (see *Milk intake rates*).

### Allonursing

Allonursing, where milk is provided to nonfilial offspring, has the potential to affect maternal energy budgets during lactation, primarily by reducing the amount of energy a female allocates to offspring while still maintaining offspring growth. In pinnipeds, observations of allonursing behavior are relatively widespread, having been observed in at least 5 phocids, odobenids and 11 otariids (e.g. Bartholomew, 1959; Riedman and Le Boeuf, 1982; Childerhouse and Gales, 2001; de Bruyn *et al.,* 2010; Pitcher *et al.,* 2011; Arso Civil *et al.,* 2021). This behavior is often attributed to misdirected parental care (e.g. misidentification by the mother) or milk theft by the pup, although there are isolated examples of extended fostering, such as when a female has lost her own pup. Fostering has also been observed in polar bears (Malenfant *et al.,* 2016) and sea otters (Staedler and Riedman, 1989), while allonursing and fostering have been observed in manatees (Bonde, 2009). Allonursing is less common in cetaceans, having been observed in sperm whales (*Physeter macrocephalus*) and captive beluga whales (*Delphinapterus leucas*) (Leung *et al.,* 2010; Konrad *et al.,* 2019), although this may be because it is difficult to observe in the wild and not because it does not occur in other species. There are also several isolated reports of adoption in wild bottlenose dolphins (Howells *et al.,* 2009; Sakai *et al.,* 2016) and one report of calf-switching in North Atlantic right whales (Frasier *et al.,* 2010). In contrast to pinnipeds, allonursing in sperm whales appears to be best explained by kin selection rather than maternal mistakes or deliberate milk stealing attempts (Konrad *et al.,* 2019).

It is largely unknown how much energy is transferred to offspring when they allosuckle nor how this impacts the energy budget or allocation decisions of the mother. The captive beluga whale calf from Leung *et al*. (2010) primarily suckled from her mother during the first 13 months, with allonursing events increasing in frequency between 15 months and 34 months of age. Overall nursing frequency decreased as the calf aged, with food consumption beginning around 12 months, suggesting that the energy gain from allonursing was likely minimal. Because of the general lack of knowledge around the frequency of allonursing and indications that energy transfer may be minimal, this behavior has not been considered when constructing energy budgets of marine mammals. One thing to consider moving forward is whether, in odontocetes where kin selection may be the driving cause, allonursing may become more energetically important with environmental change and potential reductions in prey availability. In primates, environmental conditions (high seasonality and high variability) were positively correlated with the frequency of allonursing, potentially because it increases fitness during periods of low food availability (Louppova, 2019).

### Prey supplementation

**Figure 3:**
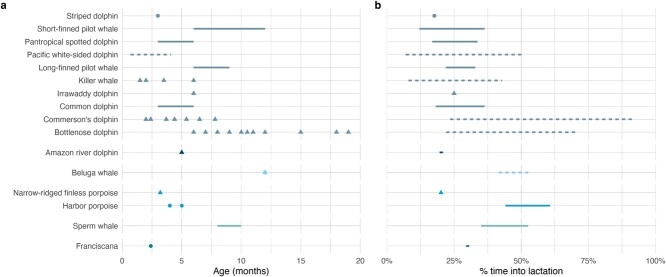
The age (a) or percent time into lactation (b) at which supplemental foraging has been documented in odontocete calves. Shapes and line types indicate whether data are from animals in the wild (circle, solid line) or managed in human care (triangle, dashed line). In b, the percent time into lactation was calculated using average lactation durations (see Fig. 2). When mean lactation duration was a range, we calculated the minimum (earliest age/longest lactation duration) and maximum (oldest age/shortest lactation duration) percent time that feeding likely occurred, with resulting values presented as a range. See Supplementary Table S22 for values and sources.

During lactation, offspring may supplement energy gained from milk with prey, either because of an actual energy need or to obtain valuable foraging and diving skills prior to independence. The occurrence of this behavior varies widely among marine mammals. In phocids, there is little indication that much (if any) foraging occurs, even for species where pups accompany mothers to sea. Prey ingestion of harbor seal (*Phoca vitulina*) pups, one of the most aquatic phocid species during pup development (Bowen *et al.,* 1999), only comprised an average of 0.7% of ingestion events (Sauvé *et al.,* 2014). In otariids, lactation duration, and potentially sex (see Piedrahita *et al.,* 2014), appears to play a role in the extent of prey supplementation; no foraging occurs in northern or Antarctic fur seal pups that suckle for roughly 4 months, whereas frequent at-sea trips and supplemental foraging have been documented for species where pups do not wean until the end of their first or second year of life (Fowler *et al.,* 2007; Lowther and Goldsworthy, 2016). Even though walrus (*Odobenus rosmarus*) calves, sea otter pups, and polar bear cubs all accompany their mothers while they are foraging, they do not independently forage with any frequency until they are in their second year of life (walrus and polar bears; Stirling and Latour, 1978; Fay, 1982) or near weaning at 20 to 24 weeks of age (sea otters; Payne and Jameson, 1984). There is at least one record of foraging by a dependent manatee (*Trichechus manatus*; Reynolds, 1981), but no mention of at what age this behavior begins or how frequently it might occur. Supplemental feeding by mysticete calves has been observed in at least one species, humpback whales (Clapham and Mayo, 1987; Szabo and Duffus, 2008), but no foraging was observed in North Atlantic right whale calves across the first 9 months of their lives, even when on the foraging grounds (Cusano *et al.,* 2019). In odontocetes, supplemental feeding appears to be widespread and occurs as early as the first month or two of age (Fig. 3, Supplementary Table S22), although the age at which it starts is highly variable among individuals. Many of these observations were based on animals in human care, which could be biased toward earlier ages, but stomach contents and behavioral data from free-ranging odontocetes generally support such findings (Fig. 3, Supplementary Table S22).

For most species, the contribution of prey to a dependent offspring’s energy budget is likely small given supplementation typically occurs late in lactation and young animals may be inefficient foragers. For mustelids and polar bears, this excludes prey provided by the mother, which occurs much earlier than independent foraging and likely contributes significantly to offspring energy needs (Payne and Jameson, 1984; Derocher and Stirling, 1998). Odontocetes appear to be an exception, where detailed records of calf food intake indicate rapid increases in the amount of prey consumed once feeding begins, with many individuals consuming similar quantities of prey to that observed at weaning in the months or even years prior to weaning (Kastelein *et al.,* 2002, 2003). Data from wild odontocete calves support these findings that maternal investment declines as the calf ages (Fruet *et al.,* 2015; Matthews and Ferguson, 2015). This indicates that prey supplementation by many odontocete calves likely plays a large role in meeting their energy needs, significantly reducing the energy demands on the female during much of lactation. It is unknown if this would hold true for deep-diving odontocetes, like sperm whales and beaked whales, where physiological constraints may restrict the ability of calves to forage. The diving capabilities of three first-year sperm whale calves were well-developed, with at least one calf reaching adult diving depths (Tønnesen *et al.,* 2018), but it is largely unknown how much foraging actually occurs on such dives.

**Figure 4:**
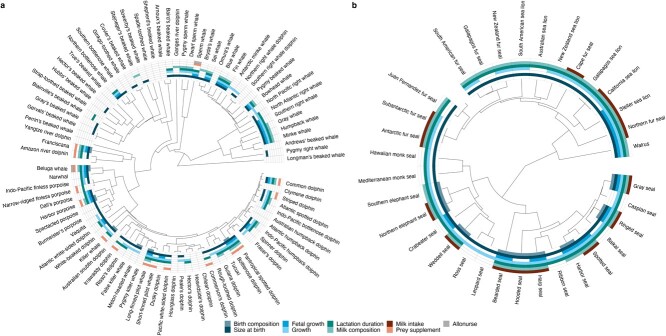
Data availability for parameters typically used to estimate reproductive costs in marine mammal bioenergetic models in cetaceans (a) and pinnipeds (b), overlaid on a phylogenetic tree. Colors correspond to different data types, with the presence of color indicative of published data (body composition at birth, length or mass at birth, fetal growth curves, milk composition, milk intake rates) or confirmation of specific behaviors (prey supplementation, allonursing; cetaceans only). Missing species labels correspond to species currently recognized as subspecies by the Society for Marine Mammalogy Taxonomic Committee in the June 2021 species list. Currently recognized species not included on the tree are: Rice’s whale (*Balaenoptera ricei*), Sato’s beaked whale (*Berardius minimus*), Deraniyagala’s beaked whale (*Mesoplodon hotaula*), the Indus river dolphin (*Platanista minor*), and the Indian Ocean humpback dolphin (*Sousa plumbea*). Phylogenetic tree created using R packages *treeio* (Wang *et al.,* 2020), *ggtree* (Yu, 2020), and *ggtreeExtra* (Xu *et al.,* 2021) using data from the VertLife Project (www.vertlife.org).

Supplemental foraging by dependent offspring is not considered in most bioenergetic models. It has been included in some odontocete bioenergetic models (New *et al.,* 2013; Bejarano *et al.,* 2017; Gallagher *et al.,* 2018; Hin *et al.,* 2019), typically by assuming some linear reduction in maternal investment. In some models, prey supplementation may be accounted for even it is not explicitly considered by shortening the lactation duration to the time across which most calf energy demands are presumed to be met by milk. Exclusion of prey supplementation from bioenergetic models may result in overestimates of maternal investment in offspring and maternal energy requirements. This could be problematic in dynamic models where behavioral decisions are dependent on maternal energy gain and maternal and offspring body condition. It is unlikely to affect population-level prey consumption estimates from accounting models since it does not change the amount of prey extracted from the ecosystem, just who is extracting it. There may be some differences though, as dependent offspring may not forage on the same prey as adult animals that are larger in size and more experienced (Field *et al.,* 2007; Lundström *et al.,* 2010; Jeglinski *et al.,* 2012). While most odontocete studies have used a linear relationship to describe the decrease in energy investment by the mother through time, this may not be the most appropriate functional form for this relationship since prey consumption of captive individuals often increases rapidly and then plateaus well before weaning (see references in Supplementary Table S22). For mustelids and polar bears, supplemental prey provided by the mother should be treated separately in bioenergetic models from that consumed by the mother to produce milk (or used for her own energy needs), since it is going directly to the offspring. Failure to separate this prey from that being ingested would result in an overestimate of prey energy needs, since there are no fecal or urinary energy losses incurred by the female.

### Multiple offspring

With the exception of polar bears and marine otters, marine mammals typically give birth to a single offspring. Twins have been reported in all taxonomic groups of marine mammals (Kawamura, 1969; Spotte, 1982; Jameson and Bodkin, 1986; Fay *et al.,* 1991; Osborn *et al.,* 2012; Davison *et al.,* 2016; Gutiérrez *et al.,* 2021; Drinkwater and Branch, 2022), but these reports are uncommon to rare. In his review on the incidence of twins in pinnipeds, Spotte (1982) noted that twins are typically underweight for their age, with no reports of both twins surviving to weaning in the wild. In polar bears, 4-month old singleton cubs weighed 25% to 35% more than those from twin litters (Derocher and Stirling, 1998), with higher survival to 6 months for singleton cubs compared with those from twin and triplet litters (Derocher and Stirling, 1996). Olesiuk *et al*. (1990) reported two occurrences of twins in killer whales (*Orcinus orca*), with one pair surviving to at least 7.5 years of age. In Hawaiian monk seals (*Neomonachus schauinslandi*), 0.14% of births resulted in twins, with a 57.1% pooled survival rate from birth to weaning. Almost all surviving twins had lower axillary girth at weaning than singleton pups born in the same year (Schultz *et al.,* 2011). These patterns of lower birth mass and the difficultly in rearing multiples are consistent with measurements from other mammals suggesting that parental cost is higher with twins than singletons (Link *et al.,* 2006; Huck *et al.,* 2014).

Most marine mammals wean their current offspring before the subsequent one is born, but there are some pinnipeds, namely the Galapagos fur seal (*Arctocephalus galapagoensis*) and Galapagos sea lion (*Zalophus wollebaeki*), where a female may concurrently nurse a new pup and an older sibling. Milk allocation to each offspring has not been measured but presumably is reduced, at least to the new pup, given growth rates and survival probability are considerably lower in pups with an older sibling compared to those without (Trillmich and Wolf, 2008). Overlap between current and future reproductive efforts (simultaneous pregnancy and lactation) commonly occurs in otariids, some odontocetes, and even some mysticetes (Pallin *et al.,* 2018). Comparisons of fetal masses between lactating and nonlactating otariids indicated that fetuses from lactating females were smaller, suggesting that simultaneous pregnancy and lactation may reduce energy investment in fetal growth (Lima and Paez, 1995; Trillmich and Wolf, 2008). Given the rare occurrence of supporting multiple offspring during one reproductive period, almost no bioenergetic models estimate costs associated with concurrently rearing more than one offspring, except of course for species where it is a common occurrence (Reimer *et al.,* 2019) and when the costs of gestation and lactation are experienced simultaneously.

## Maternal Food Intake during Gestation and Lactation

Bioenergetic models typically model the energetic costs of reproduction as additive. As a result, predicted energy needs or food intake during reproduction are almost always higher than during a nonreproductive state (assuming all else is equal). Animals do not experience the costs of reproduction in isolation though, and there may be mechanisms to help offset reproductive costs and minimize the need to increase prey intake or use of stored energy reserves. Metabolic depression, which could occur through physiological or behavioral mechanisms, has been detected in some mammals during gestation and lactation (Krockenberger, 2003; Korine *et al.,* 2004), including several pinniped species (Mellish *et al.,* 2000; Sparling *et al.,* 2006; Shuert *et al.,* 2020). While it has not been studied in marine mammals, an alternative mechanism for minimizing the need to increase food intake during reproduction is through an increase in digestive efficiency (Pereira *et al*., 2020).

The potential mismatch that any compensation mechanisms could create between actual and predicted energy needs from a bioenergetic model will depend on how likely the estimated metabolic costs are to incorporate such mechanisms. For example, this issue may largely be irrelevant for pinniped lactation costs where metabolic costs are estimated from DLW, since most of these measurements are derived from lactating females. The challenge in incorporating such compensatory mechanisms in bioenergetic models lies primarily in limitations associated with collecting data to determine the occurrence and magnitude of compensation. In wild populations, there also may be mismatches between energy needs and prey intake due to heterogeneity in prey resources and life history patterns. These potential mismatches could result in inaccurate estimates of prey intake from bioenergetic models, particularly in accounting models where estimates of prey consumption are typically calculated from energy requirements (i.e. they do not take prey availability into account).

Changes in food intake of gestating marine mammals in human care are inconsistent, with conclusions typically drawn from a small number of individuals across only part of gestation. Some studies report no change or even decreases in food intake (in kg prey) during gestation (Joseph *et al.,* 1987; Cheal and Gales, 1991; Kastelein *et al.,* 1993, 2002, 2003), while others report increases, particularly later in gestation (Asper *et al.,* 1988; Kastelein *et al.,* 1990; Reddy *et al.,* 1993; Kastelein *et al.,* 2000a; McHuron *et al.,* 2022b). There have also been conflicting findings within a species, such as for killer whales where Kastelein et al. (2003) and Williams *et al*. (2011) found no change in food intake while Asper et al. (1988) documented increases beginning in the 13th month of gestation. In one of the few studies on the cost of pregnancy in free-ranging marine mammals, Shero *et al*. (2018) documented an increase in diving effort by pregnant Weddell seals (*Leptonychotes weddelli*) compared with nonreproductive females, particularly during the last trimester, which was attributed to an increase in gestational energy demand. There are fairly consistent observations across species that food intake decreases in the days to weeks leading up to birth, regardless of whether it fluctuates at other time periods (Ronald and Thomson, 1981; Joseph *et al.,* 1987; Kastelein *et al.,* 1990, 2000b; Robeck *et al.,* 2005; McHuron *et al.,* 2022b). For some species, increases in food intake may not be related to the costs of gestation but rather due to an increase energy storage during gestation for the subsequent lactation interval (Noren *et al.,* 2014).

During lactation, most studies of marine mammals in human care report increases in food intake above pre-lactation levels (e.g. Kastelein *et al.,* 2000b, Kastelein *et al.,* 2000a, 2002, 2003; Williams *et al.,* 2007; McHuron *et al.,* 2022b). Species that rely more heavily on energy reserves during lactation may be an exception, since much of the energy for lactation is stored prior to parturition (Kastelein *et al.,* 1990). Robeck et al. (2005) did not report an increase in food intake above pre-lactation demands in beluga whales, with food intake returning to pre-parturient levels within 15 days. Whether this represents a true lack of food increase remains unknown, since there was considerable variation in both pre- and post-parturient intake, food intake was only reported for 30 days, and animals where food intake did not return to pre-parturient values within 15 days were excluded from the study.

The duration and magnitude of increase in food intake during lactation varies considerably, both within and across studies. For example, Kastelein et al. (2002) reported that food intake of bottlenose dolphins peaked 1 to 5 months after parturition, with total food intake during lactation between 48% and 98% higher than during nonreproductive periods. Asper et al. (1988) found that consumption of a single lactating killer whale peaked around 110 to 118 kg day^−1^, approximately 80% higher than intake during early gestation, with consumption remaining elevated (at 95 kg day^−1^) for six months past the time of weaning at 1.5 years of age. Reports from other species also show peak consumption estimates close to or in excess of 100% higher than nonreproductive periods (Kastelein *et al.,* 2000b, 2003; McHuron *et al.,* 2022b). Few comparative studies of food intake in wild marine mammals exist. Estimates derived from stomach contents of female northern fur seals shot at sea indicated that lactating females consumed 60% more food than nonlactating ones (Perez and Mooney, 1986). Similar data from harbor porpoises (*Phocoena phocoena*) in the Bay of Fundy indicated that lactating females consumed more than twice the prey mass and caloric content of nonlactating females (Recchia and Read, 1989). While not direct evidence, increases in diving effort throughout much of lactation from sea otters and several pinniped species are consistent with the increased food consumption documented in captive animals (Bowen *et al.,* 2001b; Hoskins and Arnould, 2013; Thometz *et al.,* 2016b).

## Factors that Affect Reproductive Energetics

Prey availability is the most influential extrinsic factor influencing reproductive investment in marine mammals. Poor environmental conditions (or poor maternal condition) reduces energetic investment in reproduction as evidenced by changes in offspring production, smaller offspring mass and reduced growth, and in extreme cases, offspring mortality (Costa *et al.,* 1989; Trites, 1992; Soto *et al.,* 2004; Christiansen *et al.,* 2014; McClatchie *et al.,* 2016; McMahon *et al.,* 2017; Gailey *et al.,* 2020; Páez-Rosas *et al.,* 2021; Smith, 2021). While it has not been studied in marine mammals, there is some evidence from other mammals that glucocorticoids in milk (indicative of maternal stress) are associated with offspring growth, potentially through changes in milk production (Stead *et al.,* 2022). Stress is a potential concern for some marine mammal populations given associations between increased glucocorticoids and ocean noise (Rolland *et al.,* 2012), warranting further investigation of how stress might affect reproductive energetics.

Maternal characteristics, such as mass and age, are well documented as influencing aspects of pinniped reproductive energetics. Maternal body size is often, although not always, positively correlated with birth mass (Boyd and McCann, 1989; Bowen *et al.,* 1994; Boltnev and York, 2001; Wheatley *et al.,* 2006). Larger females, regardless of whether they are capital or income-breeding species, often allocate more energy to their pups, either because of increased milk output, longer lactation durations, or both (Iverson *et al.,* 1993; Arnbom *et al.,* 1997; Mellish *et al.,* 1999a; Bowen *et al.,* 2001a; Crocker *et al.,* 2001; McDonald *et al.,* 2012). Changes in body volume of female southern right whales during the first three months of lactation follow a similar pattern, with longer, robust females losing volume at a faster rate than smaller, leaner ones (Christiansen *et al.,* 2018). Maternal age, irrespective of body mass, can affect factors such as milk production and milk fat content, although these differences do not necessarily translate to higher total reproductive investment (McDonald and Crocker, 2006; Lang *et al.,* 2011a, 2011b, 2012). For example, although primiparous gray seals had a reduced physiological capacity for milk production and storage compared with multiparous females, primiparous females compensated by increasing nursing frequency (Lang *et al.,* 2011a, Lang *et al.,* 2011b, Lang *et al.,* 2012). Maternal age may have other effects on offspring growth unrelated to variation in maternal energy investment, as has been shown for northern elephant seals where pups of older mothers exhibited more energy-saving behaviors (Hooper *et al.,* 2019) and gray seals where older mothers selected for birth sites that did not experience flooding (Allen *et al.,* 2022). While still a developing field, there does appear to be an individual-based component to reproductive expenditure not explained by other factors, such that some females consistently have lower or higher reproductive energy costs than others (Lang *et al*., 2009; Paterson *et al.,* 2016).

In general, the influences of intrinsic factors on reproductive effort are not explicitly incorporated in accounting or dynamic bioenergetic models. They are, however, often implicitly included since most models incorporate variation in reproductive parameters when such data are available. The effects of environmental variation on reproductive costs may be incorporated in a similar way, or explicitly modeled using year-specific values for parameters influencing reproductive costs, such as offspring growth rates and maternal activity budgets (e.g. McHuron *et al.,* 2020). In dynamic bioenergetic models, the effects of environmental variation on reproductive output may be explicitly modeled through allocation rules or functional relationships between maternal body condition and reproductive effort (e.g. Hin *et al.,* 2019; McHuron *et al.,* 2021). These attempts should be viewed as a first effort to incorporate the effect of energy deficiency on reproductive costs, as the specific mechanisms and thresholds in marine mammals that result in a reduced rate of energy delivery to offspring are largely unknown. As the focus of most accounting models is not on the individual, it is likely not overly important how intrinsic factors are accounted for when estimating reproductive costs. This may not be the case for dynamic bioenergetic models, since these are typically individual-based models and often focus on linkages between energy reserves, vital rates and population dynamics. Thus, the fact that not all individuals invest the same in reproduction could affect predicted population trajectories, particularly if environmental stressors or disturbances disproportionately impact some individuals and not others, or if there are shifts in the age structure of a population. In practice, it remains difficult to accurately incorporate these effects given limited data availability.

## Conclusions

Many pressing management and conservation questions, such as those revolving around the impacts of climate change and anthropogenic disturbance on marine mammal populations, are difficult to fully address without an understanding of how much energy a female must invest to successfully gestate and wean her offspring. Bioenergetic modeling approaches use a variety of input parameters to estimate these costs, such as offspring growth, body mass and composition, and milk composition and intake rates. Data availability for these parameters are comparatively sparse in cetaceans relative to pinnipeds, particularly for beaked whales, although data gaps remain for some polar phocids (Fig. 4). There was also extremely limited information on these parameters for sirenians and the marine otter. On the one hand, our estimates for data availability represent a minimum since we excluded references to values with an estimate but no citation. On the other hand, many estimates we included are based on observations from just a few individuals, anecdotal observations, or inferred from other data sources and are thus tentative at best.

Concerted efforts to help refine existing estimates will improve the accuracy of bioenergetic model outputs, especially those involved in calculating lactation costs. Lactation duration is one example of a parameter that is not only influential on bioenergetic model output (New *et al.,* 2013) but also one that can be estimated from free-ranging animals across all taxonomic groups. The recent work of Feyrer *et al*. (2020) and Hamilton *et al*. (2022) highlight how new data can alter our perceptions of lactation durations and relationships between lactation duration and offspring survival. Prey supplementation by odontocetes calves is another example of a parameter that deserves further attention, both in terms of understanding temporal dynamics in free-ranging animals and in how important that contribution is to a calf's energy budget. The widespread, and often early, occurrence of prey supplementation across odontocetes suggests that calf costs may rapidly exceed a female’s physiological capability to support the entirety of its energetic needs by milk. If this is true, disruptions that alter successful foraging (either environmental or anthropogenic) may be magnified because both the mother and the calf are contributing to the calf's energy needs.

Reproductive costs occur in concert with a female’s own energy needs (e.g. maintenance, locomotion, thermoregulation), but total energy budgets are not always additive since marine mammals (like other mammals) appear to have some compensatory mechanisms to cope with added reproductive costs. At present, it is difficult to incorporate such mechanisms into bioenergetic models given a general lack of understanding of how widespread these mechanisms are, their timing and magnitude, and whether there are any downstream effects on fitness (e.g. reduced immune function). Compensatory mechanisms may, however, become increasingly important in mitigating high reproductive costs since many marine mammal populations face increasingly altered prey landscapes.

## Funding

This review was funded by the Office of Naval Research (N000142012392), with support from the Marine Mammal Commission (MMC 19-173).

## Data availability

All data are incorporated into the article and its online supplementary material.

## Supplementary Material

Web_Material_coac080
