# Supplementary material for: Estimating reproductive costs in marine mammal bioenergetic models: a review of current knowledge and data availability
Source: Conserv Physiol. 2023 Jan 18;11(1):coac080. doi: 10.1093/conphys/coac080 (PMC9845964; doi:10.1093/conphys/coac080)
Supplement: Web_Material_coac080 [file web_material_coac080.zip › McHuron et al Reproductive Energetics_R1_Supplementary Tables.docx]

**Supplementary Material:** “Estimating reproductive costs in marine mammal bioenergetic models: a review of current knowledge and data availability”

Elizabeth A. McHuron^1*^, Stephanie Adamczak^2^, Daniel P. Costa^2^, Cormac Booth^3^

^1^Cooperative Institute for Climate, Ocean, and Ecosystem Studies, University of Washington, Seattle, WA, 98105, USA

^2^Ecology and Evolutionary Biology Department, University of California Santa Cruz, Santa Cruz, CA, 95064, USA

^3^SMRU Consulting, Scottish Oceans Institute, St Andrews, UK.

Number of Tables: 22

Number of Figure: 0

The supplemental tables below are intended to represent the availability of parameters relevant to bioenergetic models for marine mammals. In many cases, references and specific values do not reflect all available references as this was not the purpose of the review. For some parameters, it has been noted when estimates were tentative or believed to be tentative, usually because of small sample size. Lack of this denotation does not necessarily imply that other parameter estimates are robust. Species were classified following the Society for Marine Mammalogy Taxonomy Committee, updated June 2021, although we do separate out measurements for Australian and Guadalupe fur seals, which are currently classified as subspecies of Cape and Juan Fernandez fur seals, respectively. While we attempted to access source references, we have noted where this was not possible by citing a reference within another reference.

**Table S1.** Body length (m) and mass (kg) at birth of mysticetes. When available, mean estimates are presented; otherwise values represent point estimates. Ranges incorporate data across multiple studies when applicable. Values for Antarctic minke and pygmy right whales are not based on actual data from these species.

| Species | Length (m) | Mass (kg) | Reference |
| --- | --- | --- | --- |
| Antarctic minke whale | 2.7 - 2.9 |  | (Ohsumi *et al.*, 1970) |
| Blue whale | 7.0 |  | (Mackintosh and Wheeler, 1929) |
| Bowhead whale | 4.0 - 4.5 |  | (Koski *et al.*, 1993; Lubetkin *et al.*, 2012) |
| Bryde’s whale | 3.81 - 3.96 |  | (Best, 2001) |
| Fin whale | 6.5 |  | (Mackintosh and Wheeler, 1929) |
| Gray whale | 4.6 | 972 | (Agbayani *et al.*, 2020) |
| Humpback whale | 4.3 |  | (Chittleborough, 1958) |
| Minke whale | 2.7 | 305 | (Lockyer, 1981a) |
| North Atlantic right whale | 3.9 - 4.3 | 940 | (Christiansen *et al.*, 2020; Fortune *et al.*, 2021) |
| Pygmy right whale | 1.6 - 2.2 |  | (Ross *et al.*, 1975) |
| Sei whale | 4.4 – 4.6 |  | (Jonsgard, 1951) in (Ohsumi, 1966), |
| Southern right whale | 6.1 | 1,076 - 1,147 | (Christiansen *et al.*, 2019) |

**Table S2**. Body length (cm) and mass (kg) at birth of odontocetes. When available, mean estimates are presented; otherwise values represent point estimates. Ranges incorporate data across multiple studies when applicable. A ‘tentative’ estimate denotes when the value appeared to be based on particularly limited data, but this does not mean that other estimates without that designation should be considered well informed.

| Species | Length (cm) | Mass (kg) | Reference |
| --- | --- | --- | --- |
| Amazon river dolphin | 80.0 - 83.5 | 6.9 - 12.0 | (Boede *et al.*, 2018; Martin and Da Silva, 2018) |
| Atlantic spotted dolphin | 128.7 |  | (Siciliano *et al.*, 2007) |
| Atlantic white-sided dolphin | 108.0 - 122.0 | 25.0 | (Sergeant *et al.*, 1980; Perrin and Reilly, 1984) |
| Baiji | 70.0^a^ |  | (Chen *et al.*, 1984) |
| Baird’s beaked whale | 450.0 - 460.0 |  | (Omura *et al.*, 1955; Mead, 1984) |
| Beluga whale | 137.0 - 175.0 | 78.3 - 88.9 | (Brodie, 1971; Burns and Seaman, 1986; Heide-Jørgensen and Teilmann, 1994 and references therein; Robeck *et al.*, 2005) |
| Bottlenose dolphin | 96.2 - 120.0 | 13.8 - 14.9 | (Kasuya *et al.*, 1986; Cockcroft and Ross, 1990a, 1990b; Mattson *et al.*, 2006; Neuenhoff *et al.*, 2011; Gol’din and Gladilina, 2015) |
| Burmeister’s porpoise | 86.0 |  | (Reyes and van Waerebeek, 1995) |
| Commerson’s dolphin | 55.0 - 65.0 | 4.5 - 5.5 | (Joseph *et al.*, 1987) |
| Common dolphin | 87.0 - 93.0 | 8.7 | (Danil and Chivers, 2007; Murphy *et al.*, 2009) |
| Cuvier’s beaked whale | 270.0^b^ |  | (Mead, 1984) |
| Dall’s porpoise | 100.0 | 11.3 | (Kasuya, 1978; Ferrero and Walker, 1999) |
| Dusky dolphin | 91.0 - 102.0 | 9.6 | (Cipriano, 1992; van Waerebeek and Read, 1994) |
| Dwarf sperm whale | 103.0 | 14.0 | (Plön, 2004) |
| False killer whale | 155.0 - 175.0 | 4.9 - 6.8 | (Ferreira *et al.*, 2014) |
| Franciscana | 68.9 - 85.0^c^ |  | (Kasuya and Brownell-Jr., 1979; Harrison *et al.*, 1981; Ramos *et al.*, 2000; Denuncio *et al.*, 2018) |
| Fraser’s dolphin | 110.0 |  | (Amano *et al.*, 1996) |
| Ganges river dolphin | 70.0^b^ |  | (Kasuya, 1972a) |
| Gervais’ beaked whale | 210.0^b^ |  | (Mead, 1984) |
| Gray’s beaked whale | 242.0^b^ |  | (Hale, 1931 in Ohsumi *et al.*, 1994) |
| Guiana dolphin | 90.0 - 109.0 |  | (Ramos *et al.*, 2010) |
| Harbor porpoise | 71.3 - 76.4 |  | (Mohl-Hansen, 1954; Sørensen and Kinze, 1994; Börjesson and Read, 2003; Ólafsdóttir *et al.*, 2003; Learmonth *et al.*, 2014) |
| Hubbs’ beaked whale | 250.0^b^ |  | (Mead *et al.*, 1982) |
| Indian Ocean humpback dolphin | 104.3 - 115.0 | 14.0 | (Plön *et al.*, 2015) |
| Indo-Pacific finless porpoise | 75.0 - 85.0 | 5.0 | (Jefferson *et al.*, 2002 and references therein) |
| Indo-Pacific humpback dolphin | 101 - 111.6 |  | (Jefferson *et al.*, 2012; Nolte, 2013) |
| Irrawaddy dolphin | 91.0 - 105.0 | 10.0 - 12.0 | (Arnold and Heinsohn, 1996; Stacey and Leatherwood, 1997) |
| Killer whale | 200.0 - 244.0 | 153.8 | (Bigg, 1982; Asper *et al.*, 1988; Clark *et al.*, 2000) |
| Long-finned pilot whale | 163.1 - 178.0 | 73.8 - 75.0 | (Sergeant, 1962; Kasuya *et al.*, 1988; Bloch *et al.*, 1993; Martin and Rothery, 1993; Betty *et al.*, 2022) |
| Melon-headed whale | 94.0 - 112.0 |  | (Bryden *et al.*, 1977; Amano *et al.*, 2014) |
| Narwhal | 157.0 - 161.0 |  | (Hay, 1984; Heide-Jørgensen and Garde, 2011) |
| Northern bottlenose whale | 305.0 |  | (Benjaminsen, 1972) |
| Northern right-whale dolphin | 100.0 |  | (Ferrero and Walker, 1993) |
| Pacific white-sided dolphin | 91.0 - 94.0 | 12.0 - 14.0 | (Ferrero and Walker, 1996; Iwasaki and Kasuya, 1997 in Boy *et al.*, 2011; Dalton *et al.*, 2005) |
| Pantropical spotted dolphin | 83.2 - 91.0 |  | (Kasuya *et al.*, 1974; Hohn and Hammond, 1985) |
| Peale’s dolphin | 115.0^b^ |  | (Boy *et al.*, 2011) |
| Pygmy sperm whale | 120.0 | 53.0 | (Plön, 2004) |
| Risso’s dolphin | 99.0 - 140.0^d^ |  | (Kasuya, 1978; Kasuya and Shiraga, 1985; Amano and Miyazaki, 2004) |
| Rough-toothed dolphin | 100.0^b^ |  | (West, 2002) |
| Short-finned pilot whale | 139.5.0 - 185.0 | 37.0 | (Kasuya and Marsh, 1984; Kasuya and Matsui, 1984; Kasuya and Tai, 1993) |
| Southern bottlenose whale | 291.0^b^ |  | (Ross, 1979 in Mead, 1984) |
| Sowerby’s beaked whale | 240.0^b^ |  | (Mead, 1984) |
| Spectacled porpoise | 90.0 - 100.0 |  | (Goodall and Schiavini, 1995) |
| Sperm whale | 368.0 - 407.0 | 1,016.0 | (Best *et al.*, 1984; Clarke *et al.*, 2011 and references therein) |
| Spinner dolphin | 77.0 - 79.7 |  | (Perrin *et al.*, 1977; Larese and Chivers, 2009; Chivers *et al.*, 2019) |
| Striped dolphin | 92.5 - 116.0^e^ | 11.3 | (Miyazaki, 1977; Aguilar, 1991; Bishop, 2014) |
| Tucuxi | 106.0 |  | (Ramos *et al.*, 2000) |
| Vaquita | 70.0 - 75.0 |  | (Hohn *et al.*, 1996) |
| White-beaked dolphin | 125.0 |  | (Tomilin, 1957 in Perrin and Reilly, 1984) |

^a^Tentative estimate. Zhou (2008) lists at 91.5 cm

^b^Tentative estimate

^c^Likely closer to 70.0 cm

^d^Upper estimate tentative

^e^Upper estimate of length likely overestimated due to small sample size

**Table S3.** Birth mass (kg) of phocids. Length is not shown since mass is commonly reported. When available, mean estimates are presented; otherwise values represent point estimates. Ranges represent variation in means or point estimates. We noted when values appeared to be based on limited data (noted as tentative or potentially tentative depending on our ability to access source material).

| Species | Mass (kg) | Reference |
| --- | --- | --- |
| Baikal seal | 3.0 - 4.0^a^ | (Pastukhov, 1993 in Baranov *et al.*, 2001) |
| Bearded seal | 37.1 | (Kovacs *et al.*, 2020) |
| Caspian seal | see^b^ | (Wilson *et al.*, 2016) |
| Crabeater seal | 30.0 | (Shaughnessy *et al.*, 2019) |
| Gray seal | 14.6 - 16.3 | (Kovacs and Lavigne, 1986) |
| Harbor seal | 10.7 - 11.1 | (Ellis *et al.*, 2000) |
| Harp seal | 10.7 - 10.8 | (Stewart and Lavigne, 1980; Lydersen and Kovacs, 1996) |
| Hawaiian monk seal | 36.0 - 38.0^c^ | (Kenyon and Rice, 1957) |
| Hooded seal | 21.9 | (Mellish *et al.*, 1999b) |
| Leopard seal | 29.5^c^ | (Brown, 1952) |
| Mediterranean monk seal | 15.0 - 26.0^a^ | (Sergeant *et al.*, 1978; see references in Karamanlidis *et al.*, 2016) |
| Northern elephant seal | 35.0 | (Holser *et al.*, 2021) |
| Ribbon seal | 9.5^a^ | (Burns, 1970; Tikhomirov, 1968 in Boveng *et al.*, 2013) |
| Ringed seal | 4.6 - 5.1 | (Lydersen *et al.*, 1992; Auttila *et al.*, 2016) |
| Ross seal | 16.8^c^ | (Thomas *et al.*, 1980) |
| Southern elephant seal | 39.8 - 46.8 | (Arnbom *et al.*, 1997) |
| Spotted seal | 8.1^a,d^ | (Zhang *et al.*, 2014) also see references in (Boveng *et al.*, 2009) |
| Weddell seal | 26.7 - 30.8 | (Bryden *et al.*, 1984; Wheatley *et al.*, 2006) |

^a^Potentially tentative estimate

^b^Numerous mentions of 5.0 kg but no citations. See reference for example

^c^Tentative estimate

^d^Mention of 7 – 12 kg elsewhere but most cited literature was not original

**Table S4**. Birth mass (kg) of otariids and odobenids. Length is not shown since mass is commonly reported. Ranges represent variation in means (if available) or point estimates. We noted when values appeared to be based on limited data but that could not be confirmed (denoted as potentially tentative). Common subspecies from different geographic regions are shown separately, with the species name listed in parentheses.

| Species | Mass (kg) | Reference |
| --- | --- | --- |
| Antarctic fur seal | 4.8 - 5.8 | (Boyd and McCann, 1989; McDonald *et al.*, 2012) |
| Australian (Cape) fur seal | 7.2 - 8.3 | (Arnould and Hindell, 2002) |
| Australian sea lion | 5.2 - 6.2^a^ | (McIntosh and Kennedy, 2013) |
| California sea lion | 7.3 - 9.6^b^ | (Ono *et al.*, 1987) |
| Galapagos fur seal | 3.2 - 3.8^c^ | (Trillmich and Wolf, 2008) |
| Galapagos sea lion | 5.3 - 7.0^c^ | (Trillmich and Wolf, 2008) |
| Guadalupe (Juan Fernandez) fur seal | 4.7 - 6.3 | (Gálvez *et al.*, 2020) |
| Juan Fernandez fur seal | 5.2 - 6.4 | (Ochoa-Acuna *et al.*, 1998) |
| New Zealand fur seal | 4.0 - 4.2 | (Goldsworthy, 2006) |
| New Zealand sea lion | 9.7 - 10.6 | (Chilvers *et al.*, 2007) |
| Northern fur seal | 5.5 - 6.1 | (Boltnev *et al.*, 1998) |
| South American fur seal | 3.3 – 6.0 | (Vaz-Ferreria and Ponce de Leon, 1987 and references therein; Franco-Trecu, 2017) |
| South American sea lion | 11.4 – 14.2 | (Vaz Ferreira, 1982; Cappozzo *et al.*, 1991) |
| Steller sea lion | 18.7 - 22.4^d^ | (Davis *et al.*, 2002) |
| Subantarctic fur seal | 4.4 - 5.0 | (Guinet and Georges, 2000) |
| Walrus | 60.0 - 63.0^e^ | (Fay, 1982; Kastelein, 1994) |

^a^Deceased newborns

^b^Pups < 7 days old

^c^Pups 3 – 5 days old

^d^Pups 1 – 5 days old

^e^Potentially tentative

**Table S5.** Birth mass of mustelids, ursids, and sirenians. When available, mean estimates are presented; otherwise values represent point estimates. Ranges represent variation in means or point estimates. We noted when values appeared to be based on limited data (denoted as tentative estimate).

| Species | Mass (kg) | Reference |
| --- | --- | --- |
| Amazonian manatee | 14.6^a^ | (Carter *et al.*, 2008; also see Amaral *et al.*, 2010 and Mendoza *et al.*, 2019) |
| Dugong | 20 - 35^b^ | (Marsh *et al.*, 1984 and references therein) |
| West Indian manatee | 34.2 - 34.6 | (Borges *et al.*, 2012) |
| Polar bear | 0.65 | (Kenny and Bickel, 2005) |
| Sea otter | 1.9 - 2.3 | (Kenyon, 1969; Monnett *et al.*, 1991) |

^a^Includes body mass of two full-term fetuses

^b^Tentative estimate

**Table S6.** Growth curve availability for mysticetes. Calf focus indicates whether there was a specific analysis on young animals or when growth curves included a considerable number of samples from animals in their first year of life.

| Species | Length-Age | Weight-Age | Length-Weight | Fetal Length -Age or Weight-Age | Calf focus |
| --- | --- | --- | --- | --- | --- |
| Antarctic minke whale^a^ |  |  |  |  |  |
| Blue whale^b^ |  |  |  |  |  |
| Bryde’s whale^c^ |  |  |  |  |  |
| Bowhead whale^d^ |  |  |  |  |  |
| Fin whale^b,e^ |  |  |  |  |  |
| Sei whale^f^ |  |  |  |  |  |
| Gray whale^g^ |  |  |  |  |  |
| Humpback whale^h^ |  |  |  |  |  |
| Minke whale^i^ |  |  |  |  |  |
| North Atlantic right whale^j^ |  |  |  |  |  |
| North Pacific right whale^k^ |  |  |  |  |  |
| Southern right whale^l^ |  |  |  |  |  |

^a^(Ohsumi *et al.*, 1970; Ohsumi and Masaki, 1975)

^b^(Mackintosh and Wheeler, 1929; Frazer and Huggett, 1973; Lockyer, 1976, 1981a; Branch, 2008; Lanzetti *et al.*, 2020)

^c^(Lockyer, 1976)

^d^(Reese *et al.*, 2001; Lubetkin *et al.*, 2008, 2012; George, 2009)

^e^(Ohsumi *et al.*, 1958)

^f^(Frazer and Huggett, 1973; Lockyer, 1976, 1981a; Lanzetti *et al.*, 2020)

^g^(Lockyer, 1976; Sumich *et al.*, 2013; Agbayani *et al.*, 2020)

^h^(Frazer and Huggett, 1973; Lockyer, 1976; Stevick, 1999; Boye *et al.*, 2020; Lanzetti *et al.*, 2020)

^i^(Frazer and Huggett, 1973; Lockyer, 1981b; Markussen *et al.*, 1992; Lanzetti *et al.*, 2020)

^j^(Fortune *et al.*, 2012, 2021)

^k^(Omura *et al.*, 1969; Fortune *et al.*, 2021)

^l^(Best, 1994; Christiansen *et al.*, 2019, 2022b, 2022a)

**Table S7**. Growth curve availability for odontocetes. Calf focus indicates whether there was a specific analysis on young animals or when growth curves included a considerable number of samples from animals in their first year of life.

| Species | Length-Age | Weight-Age | Length-Weight | Fetal Length-Age or Weight-Age | Calf focus |
| --- | --- | --- | --- | --- | --- |
| Atlantic spotted dolphin^a^ |  |  |  |  |  |
| Atlantic white-sided dolphin^b^ |  |  |  |  |  |
| Baiji^c^ |  |  |  |  |  |
| Baird’s beaked whale^d^ |  |  |  |  |  |
| Beluga whale^e^ |  |  |  |  |  |
| Bottlenose dolphin^f^ |  |  |  |  |  |
| Clymene dolphin^g^ |  |  |  |  |  |
| Common dolphin^h^ |  |  |  |  |  |
| Dall’s porpoise^i^ |  |  |  |  |  |
| Dusky dolphin^j^ |  |  |  |  |  |
| Dwarf sperm whale^k^ |  |  |  |  |  |
| False killer whale^l^ |  |  |  |  |  |
| Indo-Pacific finless porpoise^m^ |  |  |  |  |  |
| Franciscana^n^ |  |  |  |  |  |
| Fraser’s dolphin^o^ |  |  |  |  |  |
| Ganges river dolphin^p^ |  |  |  |  |  |
| Guiana dolphin^q^ |  |  |  |  |  |
| Harbor porpoise^r^ |  |  |  |  |  |
| Hector’s dolphin^s^ |  |  |  |  |  |
| Indian Ocean humpback dolphin^t^ |  |  |  |  |  |
| Indo-Pacific bottlenose dolphin^u^ |  |  |  |  |  |
| Indo-Pacific humpback dolphin^v^ |  |  |  |  |  |
| Indus river dolphin^p^ |  |  |  |  |  |
| Killer whale^w^ |  |  |  |  |  |
| Long-finned pilot whale^x^ |  |  |  |  |  |
| Melon-headed whale^y^ |  |  |  |  |  |
| Narwhal^z^ |  |  |  |  |  |
| Northern bottlenose whale^aa^ |  |  |  |  |  |
| Northern right whale dolphin^ab^ |  |  |  |  |  |
| Pacific white-sided dolphin^ac^ |  |  |  |  |  |
| Pantropical spotted dolphin^ad^ |  |  |  |  |  |
| Peale’s dolphin^ae^ |  |  |  |  |  |
| Pygmy sperm whale^k^ |  |  |  |  |  |
| Risso’s dolphin^af^ |  |  |  |  |  |
| Rough-toothed dolphin^ag^ |  |  |  |  |  |
| Short-finned pilot whale^ah^ |  |  |  |  |  |
| Sperm whale^ai^ |  |  |  |  |  |
| Spinner dolphin^aj^ |  |  |  |  |  |
| Striped dolphin^ak^ |  |  |  |  |  |
| Tucuxi^al^ |  |  |  |  |  |
| Vaquita^am^ |  |  |  |  |  |
| White-beaked dolphin^an^ |  |  |  |  |  |

^a^(Siciliano *et al.*, 2007)

^b^(Sergeant *et al.*, 1980; Rogan *et al.*, 1997)

^c^(Chen and Liu, 1989; Gao and Zhou, 1992)

^d^(Kasuya, 1977)

^e^(Brodie, 1971; Burns and Seaman, 1986; Doidge, 1990a; Heide-Jørgensen and Teilmann, 1994; Robeck *et al.*, 2005; Suydam, 2009; Luque and Ferguson, 2010; Robeck *et al.*, 2015; Vos *et al.*, 2020)

^f^(Kasuya *et al.*, 1986; Cockcroft and Ross, 1990c; Read *et al.*, 1993; Mattson *et al.*, 2006; McFee *et al.*, 2010; Neuenhoff *et al.*, 2011; McFee *et al.*, 2012; Gol’din and Gladilina, 2015; Bejarano *et al.*, 2017; Venuto *et al.*, 2020)

^g^(Jefferson, 1995)

^h^(Ferrero and Walker, 1995; Danil and Chivers, 2007; Murphy *et al.*, 2009; Lanzetti *et al.*, 2020; Grandi *et al.*, 2022)

^i^(Kasuya, 1978; Ferrero and Walker, 1999)

^j^(Cipriano, 1992; van Waerebeek and Read, 1994)

^k^(Plön, 2004)

^l^(Kastelein *et al.*, 2000; Ferreira *et al.*, 2014)

^m^(Kasuya *et al.*, 1986; Shirakihara *et al.*, 1993; Jefferson *et al.*, 2002)

^n^(Kasuya and Brownell-Jr., 1979; Harrison *et al.*, 1981; Ramos *et al.*, 2000; Barreto and Rosas, 2006; Botta *et al.*, 2010; Denuncio *et al.*, 2018)

^o^(Amano *et al.*, 1996)

^p^(Braulik *et al.*, 2021). See (Kasuya, 1972a) for calf length by month

^q^(Ramos *et al.*, 2010)

^r^(Read, 1990; Sørensen and Kinze, 1994; Read and Hohn, 1995; Lockyer and Kinze, 2003; Ólafsdóttir *et al.*, 2003; Richardson *et al.*, 2003; Learmonth *et al.*, 2014)

^s^(Slooten, 1991; Webster *et al.*, 2010)

^t^(Plön *et al.*, 2015)

^u^(Kemper *et al.*, 2019)

^v^(Jefferson *et al.*, 2012; Nolte, 2013)

^w^(Bigg, 1982; Asper *et al.*, 1988; Kriete, 1995; Clark *et al.*, 2000; Kastelein *et al.*, 2003; Best *et al.*, 2010; Fearnbach *et al.*, 2011; Kotik *et al.*, 2022)

^x^(Frazer and Huggett, 1973; Kasuya *et al.*, 1988; Bloch *et al.*, 1993; Lockyer, 1993; Anabella *et al.*, 2017; Betty *et al.*, 2022)

^y^(Bryden *et al.*, 1977; Miyazaki *et al.*, 1998; Amano *et al.*, 2014)

^z^(Hay, 1984; Garde *et al.*, 2007; Heide-Jørgensen and Garde, 2011)

^aa^(Benjaminsen, 1972; Christensen, 1973; Bloch *et al.*, 1996)

^ab^(Ferrero and Walker, 1993)

^ac^(Dalton *et al.*, 1995, 2005; Ferrero and Walker, 1996; Heise, 1996)

^ad^(Kasuya *et al.*, 1974; Kasuya, 1976; Perrin *et al.*, 1976; Hohn and Hammond, 1985; Lanzetti *et al.*, 2020)

^ae^(Boy *et al.*, 2011)

^af^(Amano and Miyazaki, 2004; Chen *et al.*, 2011; Bloch *et al.*, 2012)

^ag^(West, 2002; Siciliano *et al.*, 2007)

^ah^(Kasuya and Matsui, 1984; Kasuya and Tai, 1993)

^ai^(Ohsumi, 1965; Lockyer, 1991; Evans and Hindell, 2004; Clarke *et al.*, 2011; Glarou *et al.*, 2022)

^aj^(Perrin *et al.*, 1977, 2005; Larese and Chivers, 2009; Chivers *et al.*, 2019)

^ak^(Kasuya, 1972b, 1976; Miyazaki, 1977; Calzada *et al.*, 1997; Bishop, 2014)

^al^(Ramos *et al.*, 2000; Di Beneditto and Ramos, 2004)

^am^(Hohn *et al.*, 1996)

^an^(Galatius *et al.*, 2013)

**Table S8**. Growth curve availability for phocids. We did not include information on whether growth curves focused on young age classes since most phocid studies model young-of-the-year growth separately.

| Species | Length-Age | Weight-Age | Length-Weight | Fetal Length-Age or Weight-Age |
| --- | --- | --- | --- | --- |
| Baikal seal^a^ |  |  |  |  |
| Bearded seal^b^ |  |  | No curve but data provided |  |
| Caspian seal^c^ |  |  |  |  |
| Crabeater seal^d^ |  |  |  |  |
| Gray seal^e^ |  |  |  |  |
| Harbor seal^f^ |  |  | No curve but data provided | Combined with spotted seal |
| Harp seal^g^ |  |  |  |  |
| Hawaiian monk seal^h^ |  |  |  |  |
| Hooded seal^i^ |  |  |  |  |
| Leopard seal^c^ |  |  |  |  |
| Mediterranean monk seal^j^ |  |  |  |  |
| Northern elephant seal^k^ |  |  |  |  |
| Ribbon seal^l^ |  |  |  |  |
| Ringed seal^m^ |  |  |  |  |
| Ross seal^n^ |  |  |  |  |
| Southern elephant seal^o^ |  |  |  |  |
| Spotted seal^p^ |  |  |  |  |
| Weddell seal^c,q^ |  |  |  |  |

^a^(Amano *et al.*, 2000)

^b^(McLaren, 1958; Benjaminsen, 1973; Burns and Frost, 1979; Andersen *et al.*, 1999)

^c^(McLaren, 1993)

^d^(Laws *et al.*, 2003a, 2003b)

^e^(Yunker *et al.*, 2005; Hauksson, 2007)

^f^(Naito and Nishiwaki, 1972; Burns and Gol’tsev, 1984; Hutchinson *et al.*, 2016; Harding *et al.*, 2018)

^g^(Stewart *et al.*, 1989; Hammill *et al.*, 1995; Chabot and Stenson, 2002)

^h^(Baker *et al.*, 2014)

^i^(Wiig, 1985)

^j^(Sergeant *et al.*, 1978; Murphy *et al.*, 2012)

^k^(Clinton, 1994; Holser *et al.*, 2021)

^l^(Quakenbush and Citta, 2008)

^m^(Krafft *et al.*, 2006; Chambellant *et al.*, 2012; Ferguson *et al.*, 2018)

^n^(Skinner and Klages, 1994)

^o^(Bell *et al.*, 2005)

^p^(Naito and Nishiwaki, 1972; Quakenbush *et al.*, 2009)

^q^(Bryden *et al.*, 1984)

**Table S9.** Growth curve availability for otariids and odobenids. We did not include information on whether growth curves focused on young age classes since most studies for these species model young-of-the-year growth separately. Common subspecies from different geographic regions are shown separately, with the species name listed in parentheses.

| Species | Length-Age | Weight-Age | Length-Weight | Fetal Length-Age or Weight-Age |
| --- | --- | --- | --- | --- |
| Antarctic fur seal^a^ |  |  |  |  |
| Australian (Cape) fur seal^b^ |  |  |  |  |
| California sea lion^c^ |  |  |  |  |
| Galapagos sea lion^d^ |  |  |  |  |
| New Zealand fur seal^e^ |  |  |  |  |
| New Zealand sea lion^f^ |  |  |  |  |
| Northern fur seal^g^ |  |  |  |  |
| South American fur seal^h^ |  |  |  |  |
| South American sea lion^i^ |  |  |  |  |
| Steller sea lion^j^ |  |  |  |  |
| Subantarctic fur seal^k^ |  |  |  |  |
| Walrus^l^ |  |  |  |  |

^a^(McLaren, 1993)

^b^(Arnould and Warneke, 2002)

^c^(Greig *et al.*, 2005; Laake *et al.*, 2016)

^d^(Mueller *et al.*, 2011)

^e^(Dickie and Dawson, 2003; McKenzie *et al.*, 2007)

^f^(Childerhouse *et al.*, 2010)

^g^(Trites, 1991; Trites and Bigg, 1996)

^h^(Lima and Paez, 1995)

^i^(Grandi *et al.*, 2010)

^j^(Winship *et al.*, 2001, 2002)

^k^(Bester and Van Jaarsveld, 1994; Dabin *et al.*, 2004; Authier *et al.*, 2011)

^l^(Fay, 1982; Garlich-Miller and Stewart, 1998, 1999)

**Table S10**. Growth curve availability for mustelids, sirenians, and ursids.

| Species | Length-Age | Weight-Age | Length-Weight | Fetal Length-Age or Weight-Age |
| --- | --- | --- | --- | --- |
| Amazonian manatee^a^ |  |  |  |  |
| Dugong^b^ |  |  |  |  |
| West Indian manatee^c^ |  |  |  |  |
| Polar bear^d^ |  |  |  |  |
| Sea otter^e^ |  |  |  |  |

^a^(Amaral *et al.*, 2010; Vergara-Parente *et al.*, 2010; Mendoza *et al.*, 2019)

^b^(Marsh, 1980; Goto *et al.*, 2004; Adulyanukosol *et al.*, 2009; Cherdsukjai *et al.*, 2020)

^c^(Schwarz and Runge, 2009)

^d^(Derocher and Wiig, 2002)

^e^(Laidre *et al.*, 2006; Tinker *et al.*, 2019)

**Table S11.** Body composition at birth, presented either as % of body mass that is water, protein, or fat, or the percent body mass that is muscle, blubber, and viscera. The percentage of blubber that is lipid is also shown. When available, estimates represent means.

| Species | n | Water | Protein | Fat | Muscle | Blubber | Viscera | Blubber lipid | Reference |
| --- | --- | --- | --- | --- | --- | --- | --- | --- | --- |
| *Odontocetes* |  |  |  |  |  |  |  |  |  |
| Beluga whale | 1 |  |  |  |  |  |  | 79.2 | (Gauthier *et al.*, 1998) |
| Bottlenose dolphin |  |  |  |  |  |  |  |  |  |
|  | 5 |  |  |  |  | 24.0 |  | 57.0 | (Struntz *et al.*, 2004) |
|  | 6 |  |  |  |  |  |  | 55.82 | (Dunkin *et al.*, 2005) |
| Franciscana | 4 |  |  |  |  | 32.5 |  |  | (Caon *et al.*, 2008) |
| Harbor porpoise | 12-18 |  |  |  | 13.1 | 43.4 |  | 67.9 | (Lockyer, 1995) |
| Killer whale |  |  |  |  |  |  |  |  |  |
|  | 1 |  |  |  |  |  |  | 83.7 | (Pedro *et al.*, 2017) |
|  | 2 |  |  |  |  |  |  | 42.3 - 48.0 | Pedro *et al.*, 2017) |
| Long-finned pilot whale^a^ |  |  |  |  | 17.57 | 21.97 | 14.54 | 70 | (Lockyer, 1993) |
|  |  |  |  |  |  |  |  |  |  |
| Melon-headed whale | 3 |  |  |  |  | 42.0 |  |  | (Kajiwara *et al.*, 2008) |
| *Phocids* |  |  |  |  |  |  |  |  |  |
| Bearded seal^b^ | 3 | 65.9 | 23.1 | 9.1 |  |  |  |  | (Lydersen *et al.*, 1996) |
| Gray seal |  |  |  |  |  |  |  |  |  |
|  | 6 | 70.2 | 18.6 | 4.9 |  |  |  |  | (Iverson *et al.*, 1993) |
|  | 16^c^ | 65.1 | 22.6 | 9.4 |  |  |  |  | (Lang *et al.*, 2011) |
|  | 15^d^ | 64.0 | 22.1 | 11.1 |  |  |  |  | (Lang *et al.*, 2011) |
|  | 18 | 67.9 | 23.8 | 5.3 |  |  |  |  | (Mellish *et al.*, 1999a) |
| Harbor seal | 7 | 62.9 |  | 11.3 |  |  |  |  | (Bowen *et al.*, 1992) |
| Harp seal |  |  |  |  |  |  |  |  |  |
|  | 6 | 66.0 | 23.0 | 7.0 |  |  |  |  | (Lydersen and Kovacs, 1996) |
|  | 5 | 69.8 | 21.4 | 3.0 |  |  |  |  | (Oftedal *et al.*, 1996) |
| Hooded seal |  |  |  |  |  |  |  |  |  |
|  | 14 |  |  |  | 17.3 | 30.7 | 12.0 |  | (Guerrero, 2018) |
|  | 4 | 61.9 | 19.7 | 14.0 |  |  |  |  | (Oftedal *et al.*, 1993) |
| Northern elephant seal | 9^e^ |  |  | 4.0 |  |  |  |  | (Rea and Costa, 1992) |
| Ringed seal | 3 | 70.1 |  | 4.75 |  |  |  |  | (Lydersen *et al.*, 1992) |
| Southern elephant seal | 19 |  |  | 3.8 |  |  |  |  | (Hindell, 1994) |
| Weddell seal | 25 |  | 22.8 | 8.6 |  |  |  |  | (Wheatley *et al.*, 2006) |

^a^Fetus or near-term fetus

^b^Estimated for a 40 kg fetus

^c^Primiparous females. Pups sampled at 3 days of age

^d^Multiparous females. Pups sampled at 3 days of age

^e^Pups sampled at 2 - 5 days of age

**Table S12.** Proximate composition (water, fat, and protein) of mysticete milk, separated by species. The number of samples is shown (n) when available. The original reference is shown from the citing research if we could not access it, indicated when a reference is cited within another reference.

| Species | n | Water | Protein | Fat | Reference |
| --- | --- | --- | --- | --- | --- |
| Blue whale |  |  |  |  |  |
|  | 1 | 0.5052 | - | 0.3462 | (Clowes, 1929) |
|  | 1 | 0.4162 | - | 0.3659 | (Clowes, 1929) |
|  | 1 | 0.61 | 0.12 | 0.20 | (Backhaus, 1904) in (Clowes, 1929) |
|  | 1 | 0.457 | 0.12 | 0.4 | (White, 1953) |
|  | 1 | 0.486 | 0.113 | 0.391 | (White, 1953) |
|  | 1 | 0.426 | - | 0.459 | (Zenkovich, 1938) |
|  | 1 | 0.505 | - | 0.346 | (Zenkovich, 1938) |
|  | 1 | - | 0.1076 | 0.4985 | (Gregory *et al.*, 1955) |
|  | 1 | - | 0.1356 | 0.3479 | (Gregory *et al.*, 1955) |
|  | 1 | - | 0.1529^a^ | 0.031^a^ | (Gregory *et al.*, 1955) |
|  | 1 | 0.605 | 0.124 | 0.2 | (Oftedal, 1997) |
|  | - | 0.472 | 0.128 | 0.381 | (Heyerdahl, 1930) in (Siversten, 1941) |
|  | - | - | - | 0.50 | (Kirpichnikov, 1949)^b^ in (Tomilin, 1957) in (Lockyer, 1984) |
|  | - | - | 0.141 | 0.390 | (Schmidt-Nielsen and Frog, 1933) in (Siversten, 1941) |
| Bowhead whale |  |  |  |  |  |
|  | 1 | 0.698 | 0.094 | 0.194 | (Scheibe, 1908) in (Oftedal, 1997) |
|  | 1 | 0.488 | 0.07 | 0.438 | Unknown in (Yablokov *et al.*, 1974) |
| Bryde’s whale | 1 | 0.521 | 0.147 | 0.296 | (Best, 1960) in (Oftedal, 1997) |
| Fin whale |  |  |  |  |  |
|  | 1 | 0.5419 | - | 0.302 | (Clowes, 1929) |
|  | 1 | 0.534 | 0.133 | 0.33 | (Ohta *et al.*, 1953) |
|  | 1 | 0.55 | 0.123 | 0.318 | (Ohta *et al.*, 1953) |
|  | 1 | 0.541 | 0.105 | 0.325 | (Ohta *et al.*, 1953) |
|  | 1 | 0.579 | 0.114 | 0.286 | (Lauer and Baker, 1969) |
|  | 1 | 0.481 | 0.113 | 0.397 | (White, 1953) |
|  | 1 | 0.618 | 0.131 | 0.185 | (White, 1953) |
|  | 1 | 0.762 | 0.036 | 0.171 | (White, 1953) |
|  | 1 | 0.38 | 0.085 | 0.51 | (White, 1953) |
|  | 1 | 0.45 | 0.121 | 0.378 | (White, 1953) |
|  | 1 | 0.5 | 0.102 | 0.378 | (White, 1953) |
|  | 1 | 0.42 | - | 0.444 | (Zenkovich, 1938) |
|  | 1 | 0.465 | - | 0.42 | (Zenkovich, 1938) |
|  |  | 0.6186 | 0.1119 | 0.222 | (Takata, 1921) |
|  | 2 | - | 0.104 | 0.336 | (Schmidt-Nielsen and Frog, 1933) in (Oftedal, 1997) |
|  | - | 0.541 | 0.131 | 0.306 | (Heyerdahl, 1930) in (Siversten, 1941) |
| Gray whale | 3 - 5 | 0.507 | 0.115 | 0.353 | (Zenkovich, 1938) |
| Humpback whale |  |  |  |  |  |
|  | 1 | - | 0.131 | 0.392 | (Chittleborough, 1958) |
|  | 1 | - | 0.125 | 0.326 | (Chittleborough, 1958) |
|  | 1 | - | 0.136 | 0.37 | (Chittleborough, 1958) |
|  | 1 | - | 0.107 | 0.413 | (Chittleborough, 1958) |
|  | 1 | - | 0.118 | 0.255 | (Chittleborough, 1958) |
|  | 1 | - | - | 0.266 | (Chittleborough, 1958) |
|  | 1 | - | - | 0.325 | (Chittleborough, 1958) |
|  | 1 | - | - | 0.32 | (Chittleborough, 1958) |
|  | 1 | - | - | 0.336 | (Chittleborough, 1958) |
|  | 1 | - | - | 0.285 | (Chittleborough, 1958) |
|  | 1 | - | - | 0.268 | (Chittleborough, 1958) |
|  | 1 | - | - | 0.318 | (Chittleborough, 1958) |
|  | 1 | - | 0.03 | 0.543 | (Chittleborough, 1958) |
|  | 1 | - | 0.124 | 0.204 | (Chittleborough, 1958) |
|  | 1 | 0.4748 | - | 0.3848 | (Zenkovich, 1938) |
|  | - | 0.4 | 0.1 | 0.48-0.52 | Unpublished data in (Lockyer, 1984) |
|  | 1 | 0.467 | - | 0.385 | (Pedersen, 1952) in (Oftedal, 1997) |
|  | 3 | 0.401 | - | 0.473 | (Symons and Weston, 1958) in (Oftedal, 1997) |
| Minke whale |  |  |  |  |  |
|  | 7 | 0.608 | 0.146 | 0.203 | (Best, 1982) in (Oftedal, 1997) |
|  | 16 | 0.519 | 0.136 | 0.302 | (Best, 1982) in (Oftedal, 1997) |
| Sei whale |  |  |  |  |  |
|  | 2 - 4 | 0.583 | 0.1 | 0.268 | (Takemura, 1927; Schmidt-Nielsen and Frog, 1933) in (Oftedal, 1997) |
|  | - | 0.591 | 0.126 | 0.262 | McConnell pers. comm. in (Lockyer, 1984) |
|  | - | - | 0.12^c^ | 0.222^c^ | (Takata, 1921) in (Lockyer, 1984) |

^a^Abnormal sample

^b^Not in references because it was missing from references in Lockyer (1984). Likely Kirpichnikov, A. A. (1949). On milk composition in whales and on their feeding with plankton Crustacea. Priroda, 10, p. 72, Leningrad

^c^Could not find reference to sei whales in Takata 1921, but reference is in German.

**Table S13.** Proximate composition (water, protein, fat) of odontocete milk, separated by species. The number of samples is shown (n) when available. The original reference is shown from the citing research if we could not access it, indicated when a reference is cited within another reference.

| Species | n | Water | Protein | Fat | Reference |
| --- | --- | --- | --- | --- | --- |
| Amazon river dolphin |  |  |  |  |  |
|  | 1 |  | 0.096 | 0.069 | (Rosas and Lehti, 1996) |
|  | 1 |  | 0.075 | 0.130 | (Gewalt, 1978) in (Rosas and Lehti, 1996) |
| Atlantic spotted dolphin | 1 | 0.6898 | 0.0942 | 0.180 | (Eichelberger *et al.*, 1940) |
| Beluga whale | 1 | 0.59 | 0.106 | 0.269 | (Lauer and Baker, 1969) |
| Bottlenose dolphin |  |  |  |  |  |
|  | 1 | 0.5830 | 0.0680 | 0.3300 | (Jenness and Sloan, 1970) in (West *et al.*, 2007) |
|  | 1 |  |  | 0.1900 | (Ackman *et al.*, 1971) |
|  | 4 |  | 0.122 | 0.294 | (Pervaiz and Brew, 1986) |
|  | 1 | 0.7279 | 0.1210 | 0.1315 | (Peddemors *et al.*, 1989) |
|  | 1 |  | 0.1160 | 0.0600 | (Ridgway *et al.*, 1995) |
|  | 1 |  | 0.1400 | 0.0770 | (Ridgway *et al.*, 1995) |
|  | 1 |  | 0.1500 | 0.0840 | (Ridgway *et al.*, 1995) |
|  | 1 |  | 0.1240 | 0.1360 | (Ridgway *et al.*, 1995) |
|  | 1 |  | 0.1330 | 0.2350 | (Ridgway *et al.*, 1995) |
|  | 1 |  | 0.1270 | 0.2650 | (Ridgway *et al.*, 1995) |
|  | 1 |  | 0.0840 | 0.1030 | (Ridgway *et al.*, 1995) |
|  | 1 |  | 0.0470 | 0.0570 | (Ridgway *et al.*, 1995) |
|  | 1 |  | 0.1090 | 0.2250 | (Ridgway *et al.*, 1995) |
|  | 1 |  | 0.0970 | 0.2000 | (Ridgway *et al.*, 1995) |
|  | 1 |  | 0.0740 | 0.1000 | (Ridgway *et al.*, 1995) |
|  | 1 |  | 0.0980 | 0.2040 | (Ridgway *et al.*, 1995) |
|  | 1 |  | 0.0980 | 0.2190 | (Ridgway *et al.*, 1995) |
|  | 1 |  | 0.1410 | 0.1930 | (Ridgway *et al.*, 1995) |
|  | 1 |  | 0.1830 | 0.2070 | (Ridgway *et al.*, 1995) |
|  | 64^a^ | 0.7300 | 0.0890 | 0.1280 | (West *et al.*, 2007) |
|  | 1 | 0.7137 | 0.0964 | 0.167 | (Eichelberger *et al.*, 1940) |
|  | 1 | 0.6742 | 0.1123^b^ | 0.148 | (Eichelberger *et al.*, 1940) |
|  | 1 | 0.7555 | 0.1111 | 0.108 | (Eichelberger *et al.*, 1940) |
| Common dolphin |  |  |  |  |  |
|  | 1 | 0.5187 | 0.0909 | 0.3733 | (Peddemors *et al.*, 1989) |
|  | 1 | 0.5838 | 0.1030 | 0.3000 | (Peddemors *et al.*, 1989) |
|  | - | 0.577 | 0.113 | 0.292 | (Ross, 1984) |
| Dwarf sperm whale | 1 | 0.622 | 0.106 | 0.185 | (Oftedal, 1997) |
| Franciscana |  |  |  |  |  |
|  | 1 |  | 0.1370 | 0.0821 | (Caon *et al.*, 2008) |
|  | 1 |  | 0.1443 | 0.0822 | (Caon *et al.*, 2008) |
|  | 1 |  | 0.1150 | 0.1900 | (Caon *et al.*, 2008) |
|  | 1 |  | 0.0950 | 0.1380 | (Caon *et al.*, 2008) |
|  | 1 |  | 0.0980 | 0.1400 | (Caon *et al.*, 2008) |
|  | 1 |  | 0.137 | 0.0821 | (Secchi *et al.*, 1994) in (Rosas and Lehti, 1996) |
|  | 1 |  | 0.144 | 0.0820 | (Secchi *et al.*, 1994) in (Rosas and Lehti, 1996) |
| Ganges river dolphin | 1 |  |  | 0.13^c^ | (Kannan *et al.*, 1997; Senthilkumar *et al.*, 1999) |
| Ginkgo-toothed beaked whale |  |  |  |  |  |
|  | 1 | 0.6650 | 0.1760 | 0.1460 | (Sasaki *et al.*, 2004) |
|  | 1 | 0.4840 | 0.1580 | 0.3480 | (Sasaki *et al.*, 2004) |
| Guiana dolphin | 1 |  | 0.095 | 0.171 | (Rosas and Monteiro-Filho, 2002) |
| Harbor porpoise | 1 | 0.4111 | 0.119 | 0.4580 | (Purdie, 1885) in (M’Intosh, 1885; Allen, 1886) |
| Indian Ocean humpback dolphin | 1 | 0.7665 | 0.1130 | 0.1022 | (Peddemors *et al.*, 1989) |
| Indo-Pacific humpback dolphin | 1 |  |  | 0.0199^c^ | (Parsons and Chan, 1998) |
| Long-finned pilot whale |  |  |  |  |  |
|  | 1 | 0.4787 | 0.0772^d^ | 0.4441 | (Frankland and Hambly, 1890) |
|  | 1 | 0.4947 | 0.0742^d^ | 0.4311 | (Frankland and Hambly, 1890) |
| Narrow-ridged finless porpoise |  |  |  |  |  |
|  | 1 | 0.791 | 0.0973 | 0.0708 | (Zeng *et al.*, 2017) |
|  | 1 | 0.623 | 0.109 | 0.2507 | (Zeng *et al.*, 2017) |
|  | 1 | 0.659 | 0.207 | 0.0954 | (Zeng *et al.*, 2017) |
|  | 1 | 0.763 | 0.116 |  | (Zeng *et al.*, 2017) |
|  | 1 | 0.679 | 0.103 | 0.192 | (Zeng *et al.*, 2017) |
|  | 1 | 0.675 | 0.091 | 0.205 | (Zeng *et al.*, 2017) |
|  | 1 | 0.675 | 0.108 |  | (Zeng *et al.*, 2017) |
|  | 1 | 0.588 | 0.087 | 0.262 | (Zeng *et al.*, 2017) |
|  | 1 | 0.691 | 0.104 | 0.197 | (Zeng *et al.*, 2017) |
|  | 1 | 0.733 | 0.0982 |  | (Zeng *et al.*, 2017) |
|  | 1 | 0.692 | 0.107 |  | (Zeng *et al.*, 2017) |
|  | 1 | 0.654 | 0.0987 |  | (Zeng *et al.*, 2017) |
| Pantropical spotted dolphin |  |  |  |  |  |
|  | 1 |  | 0.0777 | 0.1800 | (Pilson and Waller, 1970) |
|  | 1 |  | 0.0781 | 0.2340 | (Pilson and Waller, 1970) |
|  | 1 |  | 0.0788 | 0.2980 | (Pilson and Waller, 1970) |
|  | 1 |  | 0.0815 | 0.2720 | (Pilson and Waller, 1970) |
|  | 1 |  | 0.0930 | 0.1700 | (Pilson and Waller, 1970) |
|  | 1 |  | 0.0778 | 0.2580 | (Pilson and Waller, 1970) |
|  | 1 |  | 0.0795 | 0.2980 | (Pilson and Waller, 1970) |
|  | 1 |  | 0.0956 | 0.3170 | (Pilson and Waller, 1970) |
| Pygmy sperm whale | 1 | 0.743 | 0.082 | 0.153 | (Jenness and Odell, 1978) |
| Sperm whale |  |  |  |  |  |
|  | 1 |  | 0.077 | 0.199 | (Best *et al.*, 1984) |
|  | 1 |  | 0.105 | 0.25 | (Best *et al.*, 1984) |
|  | 1 |  | 0.105 | 0.208 | (Best *et al.*, 1984) |
|  | 1 |  | 0.115 | 0.269 | (Best *et al.*, 1984) |
|  | 1 |  | 0.091 | 0.175 | (Best *et al.*, 1984) |
|  | 1 |  | 0.093 | 0.276 | (Best *et al.*, 1984) |
|  | 1 |  | 0.087 | 0.22 | (Best *et al.*, 1984) |
|  | 1 |  | 0.085 | 0.224 | (Best *et al.*, 1984) |
|  | 1 |  | 0.088 | 0.232 | (Best *et al.*, 1984) |
|  | 1 |  | 0.083 | 0.28 | (Best *et al.*, 1984) |
|  | 1 |  | 0.095 | 0.341 | (Best *et al.*, 1984) |
|  | 1 |  | 0.071 | 0.253 | (Best *et al.*, 1984) |
|  | 1 |  | 0.089 | 0.248 | (Best *et al.*, 1984) |
|  | 1 | 0.549 |  | 0.362 | (Berzin, 1971) in (Best *et al.*, 1984) |
|  | 2 |  |  | 0.365 | (Aguilar, 1983) |
| Stejneger's beaked whale |  |  |  |  |  |
|  | 1 |  | 0.117 | 0.351 | (Komura *et al.*, 2002) |
|  | 1 |  | 0.167 | 0.170 | (Ullrey *et al.*, 1984) |
| Spinner dolphin | 1 |  | 0.0709 | 0.262 | (Pilson and Waller, 1970) |

^a^From three individuals

^b^Protein based on Total N and average non-protein N from two other dolphins

^c^From stomach

**Table S14.** Proximate composition (water, protein, fat) of pinniped milk, separated by species. Ranges represent means at different time periods of lactation or from different years or studies, depending on how the data were presented in each paper. The number of samples is shown (n) as well as the reference, with a (-) indicating sample size could not be determined from the reference. The original reference is shown from the citing research if we could not access, indicated when a reference is cited within another reference. When data from multiple studies are presented, the sample size represents the sum across the studies. Common subspecies from different geographic regions are shown separately, with the species name listed in parentheses.

| Species | n | Water | Protein | Fat | Reference |
| --- | --- | --- | --- | --- | --- |
| *Odobenid* |  |  |  |  |  |
| Walrus | 3 - 4 | 0.599 | 0.0766 | 0.261 | (Fay, 1982) |
|  |  |  |  |  |  |
| *Otariid* |  |  |  |  |  |
| Antarctic fur seal | 89 | 0.448 | 0.104 | 0.405 | (Arnould and Boyd, 1995) |
| Australian (Cape) fur seal | 89 | 0.382 - 0.596 | 0.098 - 0.106 | 0.282 - 0.491 | (Arnould and Hindell, 1999) |
| Australian sea lion | - | 0.624 | 0.105 | 0.254 | (Kretzmann *et al.*, 1991) |
| California sea lion | - | 0.59 | 0.086 | 0.317 | Oftedal et al., 1983 unpub. data in (Oftedal *et al.*, 1987) |
| Cape fur seal | 17 | 0.581 | 0.108 | 0.232 | (Gamel *et al.*, 2005) |
| Galapagos fur seal | 19 | - | 0.121 | 0.251 - 0.324 | (Trillmich and Lechner, 1986) |
| Galapagos sea lion | 1 | - | 0.073 | 0.212 | (Trillmich and Lechner, 1986) |
| Guadalupe (Juan Fernandez) fur seal | - | - | - | 0.430 | (Figueroa-Carranza, 1994) in (Gallo-Reynoso and Figueroa-Carranza, 2010) |
| Juan Fernandez fur seal | 44 | - | 0.119 | 0.414 | (Ochoa-Acuna *et al.*, 1999) |
| New Zealand fur seal | - |  |  | 0.47 | (Baylis and Nichols, 2009) |
| New Zealand sea lion | 308 | 0.679 | 0.092 | 0.213 | (Riet-Sapriza *et al.*, 2012) |
| Northern fur seal | 102 | 0.297 - 0.413 | 0.108 - 0.115 | 0.412 - 0.583 | (Donohue *et al.*, 2002) |
| Steller sea lion | 25 | 0.527 - 0.651 | 0.029 - 0.0411 | 0.313 - 0.366 | (Adams *et al.*, 1996) |
| Subantarctic fur seal | 101 | 0.333 - 0.510 | 0.108 - 0.134 | 0.366 - 0.523 | (Georges *et al.*, 2001) |
| South American fur seal | - |  |  | 0.306 - 0.554 | (Ponce de Leon, 1984) in (Werner, 1996) |
| South American sea lion | - | 0.489 | 0.111 | 0.386 | (Werner, 1996) |
|  |  |  |  |  |  |
| *Phocid* |  |  |  |  |  |
| Bearded seal | 8 |  | 0.088 - 0.103 | 0.422 - 0.477 | (Lydersen *et al.*, 1996) |
| Gray seal | 98 | 0.284 - 0.500 | 0.090 - 0.110 | 0.345 - 0.596 | (Lydersen *et al.*, 1995; Mellish *et al.*, 1999a) |
| Harbor seal | 55 | 0.371 - 0.462 | 0.083 - 0.099 | 0.408 - 0.509 | (Lang *et al.*, 2005) |
| Harp seal | 17 | 0.324 - 0.680 | 0.0656 - 0.104 | 0.23 - 0.571 | (Lavigne *et al.*, 1982; Oftedal *et al.*, 1996) |
| Hooded seal | 28 | 0.313 - 0.338 | 0.047 - 0.067 | 0.517 - 0.611 | (Oftedal *et al.*, 1988; Mellish *et al.*, 1999b) |
| Northern elephant seal | 63 | 0.328 - 0.660 | 0.090 - 0.121 | 0.194 - 0.544 | (Le Boeuf and Ortiz, 1977; Kretzmann *et al.*, 1993; McDonald and Crocker, 2006) |
| Ringed seal | 3 | 0.486 | 0.099 | 0.381 | (Lydersen *et al.*, 1992) |
| Southern elephant seal | 63 | 0.374 - 0.538 | 0.092 - 0.127 | 0.161 - 0.488 | (Carlini *et al.*, 1994; Hindell, 1994) |
| Spotted seal | 2 | 0.355 - 0.586 | 0.072 - 0.090 | 0.312 - 0.515 | (Zhang *et al.*, 2014) |
| Weddell seal | 21 | 0.338 | 0.101 | 0.54 | (Eisert *et al.*, 2013) |

**Table S15**. Proximate composition in milk (water, protein, fat) of sirenians, mustelid, and ursids. Ranges represent means at different time periods of lactation or from different studies. The number of samples is also shown (n).

| Species | n | Water | Protein | Fat | Reference |
| --- | --- | --- | --- | --- | --- |
| West Indian manatee | 7 |  | 0.069 - 0.097 | 0.071 - 0.215 | (Bachman and Irvine, 1979; Pervaiz and Brew, 1986) |
| Sea otter | 4 | 0.57 - 0.68 | 0.089 - 0.123 | 0.209 - 0.236 | (Jenness *et al.*, 1981) |
| Polar bear | 135 |  | 0.091 - 0.132 | 0.206 - 0.358 | (Derocher *et al.*, 1993) |

**Table S16.** Milk intake rates of pinnipeds and ursids. Mass estimates (in kg) are means (or a range of means) between initial and final measurements, different time periods, years, or studies, depending on how data were presented. If mass was not explicitly given, it was estimated based on information provided in the article, such as birth mass and growth rates. For polar bears, mass is based on cubs in single litters. Age (in days) represents the average age or age range of pups or cubs during milk intake measurements. An age of zero denotes birth. Common subspecies from different geographic regions are shown separately, with the species name listed in parentheses.

| Species | Milk intake (kg day^-1^) | Mass (kg) | Age (days) | Reference |
| --- | --- | --- | --- | --- |
| *Phocid* |  |  |  |  |
| Bearded seal | 7.6 | 57.4 | <7 - ~12 | (Lydersen *et al.*, 1996) |
| Gray seal | 1.2 – 3.5 | 16.6 – 43.3 |  | (Iverson *et al.*, 1993; Mellish *et al.*, 1999a) |
| Harp seal | 3.5 – 3.8 | 19.5 – 21.9 | 1 - 11 | (Lydersen and Kovacs, 1996) |
| Hooded seal | 7.5 – 10.4 | 30.8 – 33.3 | 1 - 4 | (Oftedal *et al.*, 1993; Lydersen *et al.*, 1997) |
| Northern elephant seal | 5.0 – 5.5 | 94 | 0 - 24 | (Ortiz *et al.*, 1984; Costa *et al.*, 1986) |
| Ringed seal | 1.4^a^ | 17.9 |  | (Lydersen and Hammill, 1993) |
| Spotted seal | 1.5 – 1.8^b^ | 8.6 – 17.0 | 0 - 10 | (Zhang *et al.*, 2014) |
| Weddell seal | 3.5^a^ | 68.4 | 8 - 27 | (Tedman and Green, 1987) |
|  |  |  |  |  |
| *Otariid* |  |  |  |  |
| Antarctic fur seal | 0.71 – 0.84^c^ | 5.3 – 11.8 | 0 - 57 | (McDonald *et al.*, 2012) |
| Australian (Cape) fur seal | 0.35 – 0.90 | 7.5 – 29.6 | 9 - 278 | (Arnould and Hindell, 2002) |
| California sea lion | 0.61 – 0.72 | 8.5 – 9.8 | 5 - 28 | (Oftedal *et al.*, 1987) |
| Cape fur seal | 0.94^a,d^ | 6.9 | 30 | (Gamel *et al.*, 2005) |
| Northern fur seal | 0.43 – 0.72 | 7.4 – 13.7 | 22 – 95 | (Donohue *et al.*, 2002) |
| Steller sea lion | 1.78 – 2.12 | 25.4 – 26.8 | 5 - 40 | (Higgins *et al.*, 1988; Davis *et al.*, 2002) |
| Subantarctic fur seal | 0.66 | 10.5 | 66 | (Arnould *et al.*, 2003) |
|  |  |  |  |  |
| *Ursidae* |  |  |  |  |
| Polar bear | 0.23 – 0.68 | 35.3 – 87.0 | 243 – 608 | (Arnould and Ramsay, 1994) |

^a^Reported in L day^-1^

^b^Captive pup that was fed milk express from a female that lost her pup

^c^Converted to kg from MJ using milk energy density values from reference text

^d^Converted from mass-specific value using a mass of 6.89 kg

**Table S17.** Lactation duration of mysticetes. When available, values are presented as means with standard deviations and ranges in parentheses. Source refers to whether the lactation duration was derived from data collected from harvested (commercial or subsistence), free-ranging, or captive (managed in human care) whales. Method refers to the general category of the methodology used: SC = stomach contents, S = samples (such as measurements, milk in mammary glands), C = composition of harvested animals, and O = direct observations.

| Species | Duration (days) | Reference | Source-Method |
| --- | --- | --- | --- |
| Blue whale | 213 | (Mackintosh and Wheeler, 1929) | Harvested - SC/S |
| Bowhead whale | 182 - 365^a^ | (Nerini *et al.*, 1984) | Harvested - S |
| Fin whale | 182.5 | (Mackintosh and Wheeler, 1929) | Harvested - SC/S |
| Humpback whale |  |  |  |
|  | 368 ± 18.4 (345 - 371) | (Baker *et al.*, 1987) | Free-ranging - O |
|  | 365^b^ | (Barendse *et al.*, 2013) | Free-ranging - O/S |
|  | 319 | (Chittleborough, 1958) | Harvested - S |
| Gray whale |  |  |  |
|  | 213 | (Rice and Wolman, 1971) | Harvested - SC/S |
|  | 213^c^ | (Wahrenbrock *et al.*, 1974) | Captive - O |
|  | 213 – 243^c^ | (Reidarson *et al.*, 2001) | Captive - O |
|  | 183 - 243 | (Weller *et al.*, 1999) | Free-ranging - O |
| Minke whale | 121 - 182 | (Lockyer, 1984)^d^ | Harvested |
| North Atlantic right whale |  |  |  |
|  | 365 ± 39.8 (290 - 431) | (Hamilton and Cooper, 2010) | Free-ranging - O |
|  | 365 (243 - 517) | (Hamilton *et al.*, 1995) | Free-ranging - O |
| Sei whale |  |  |  |
|  | 273 | (Rice, 1977) | Harvested - C |
|  | 152 | (Matthews, 1938) | Unclear, possibly Free-ranging - O |
|  | 213 | (Masaki, 1976) | Harvested – C/Free-ranging - O |
| Southern right whale |  |  |  |
|  | 364.6 ± 33.1 (303 - 419)^b^ | (Burnell, 2001) | Free-ranging - O |
|  | 412 (395 - 456)^e^ | (Thomas and Taber, 1984) | Free-ranging - O |
|  | 213 - 243 | (Tormosov *et al.*, 1998) | Harvested - C |

^a^Recent literature was somewhat circular and it was largely unclear from newer studies if time of weaning was inferred from their data or the literature

^b^Maximum estimate

^c^Based on timing of when they were weaned from formula

^d^Not the original reference. Numerous references were cited but there was very little reference in these original materials to lactation durations. These values may have come from observations that lactating females were absent from the foraging grounds coupled with average time of birth estimates.

^e^Lactation duration is from a single calf. Range given is approximate

**Table S18.** Lactation duration of odontocetes by species. When available, values represent means. Minimum and maximum values are provided in parentheses when available. The original reference is provided when we either could not access what was noted as the source of the data or when we could not find reference to the specific value in the original reference (see footnotes), indicated when a reference is cited within another reference. Source refers to whether the lactation duration was derived from data collected from harvested (commercial or subsistence), free-ranging, captive (managed in human care), stranded, or bycaught whales. Method refers to the general category of the methodology used: SC = stomach contents, S = samples (such as measurements, milk in mammary glands), C = composition of harvested animals, and O = direct observations.

| Species | Duration (days) | Reference | Source - Method |
| --- | --- | --- | --- |
| Amazon river dolphin |  |  |  |
|  | 1022 ± 985 (547 - 2117) | (Martin and Da Silva, 2018) | Free-ranging - O |
|  | 537 ± 76 | (Boede *et al.*, 2018) | Captive - O |
| Atlantic spotted dolphin | 1095 (max of 1825) | (Herzing, 1997) | Free-ranging - O |
| Atlantic white-sided dolphin | 547 | (Sergeant *et al.*, 1980) | Harvested - C |
| Baird’s beaked whale | < 182 | (Subramanian *et al.*, 1988) | Harvested - S |
| Blainville’s beaked whale | 986 | Pers comm. in (New *et al.*, 2013) | Unknown |
| Beluga whale |  |  |  |
|  | 730 | (Brodie, 1971) | Harvested - C |
|  | 638 | (Sergeant, 1973) | Harvested - C |
|  | 972 | (Doidge, 1990b) | Harvested - C |
|  | 511 | (Kleinenberg *et al.*, 1969) in (Doidge, 1990b) | Unknown |
|  | 949 | (Kleinenberg *et al.*, 1969) in (Doidge, 1990b) | Unknown |
|  | >365 and <1460^a^ | (Matthews and Ferguson, 2015) | Harvested - S |
| Bottlenose dolphin |  |  |  |
|  | 321 | (Cockcroft, 1989) | Free-ranging - O |
|  | 1891 | (Cockcroft, 1989) | Free-ranging - O |
|  | 213.5 | (Ridgway and Benirschke, 1977) in (Peddemors *et al.*, 1992) | Unknown |
|  | 1251 (985 - 2920)^b^ | (Mann *et al.*, 2000) | Free-ranging - O |
|  | 730 | (Fruet *et al.*, 2015) | Stranded - S |
|  | 638-700 | (Sergeant, 1962) | Captive - O |
|  | 730 | (Reddy *et al.*, 1993) | Captive - O |
|  | 1058 (730 – 1460) | (Baker *et al.*, 2018) | Free-ranging - O |
|  | 547 | (Tavolga and Essapian, 1957) in (Ohsumi, 1965) | Captive - O |
| Commerson’s dolphin |  |  |  |
|  | 152 | (Kastelein *et al.*, 1993) | Captive - O |
|  | 365 | (Joseph *et al.*, 1987) | Captive - O |
| Common dolphin | 502 | (Danil and Chivers, 2007) | Bycaught - C |
| Dall’s porpoise |  |  |  |
|  | 730 (182-1277) | (Kasuya, 1978) | Harvested - C |
|  | <304 | (Newby, 1982) | Harvested - S |
| Dusky dolphin | 365 | (van Waerebeek and Read, 1994) | Harvested - C |
| Dwarf sperm whale | 182 - 730 | (Plön, 2004) | Stranded - S |
| Finless porpoise |  |  |  |
| *Indo-Pacific* | 182 - 465 | (Kasuya and Kureha, 1979) | Free-ranging - O |
| *Narrow-ridged* | 483 | (Xian *et al.*, 2012) | Captive - O |
| Franciscana |  |  |  |
|  | 274.5 | (Harrison *et al.*, 1981) | Bycaught - S |
|  | 243 - 273 | (Kasuya and Brownell-Jr., 1979) | Bycaught - C |
|  | 196 | (Denuncio *et al.*, 2013) | Stranded/bycaught - SC |
| Guiana dolphin | 265 | (Rosas and Monteiro-Filho, 2002) | Bycaught - SC |
| Harbor porpoise |  |  |  |
|  | 273 | (Sørensen and Kinze, 1994) | Stranded/bycaught - SC |
|  | 213.5 | (Ólafsdóttir *et al.*, 2003) | Bycaught - C |
|  | 273 | (Read, 1990) | Bycaught - S/U |
|  | 243 - 344 | (Learmonth *et al.*, 2014) | Stranded - C/SC |
| Hector’s dolphin | 365^c^ | (Manning and Grantz, 2017) | Stranded/bycaught - SC |
| Indian Ocean humpback dolphin | 730^d^ | (Cockcroft, 2002) in (Plön *et al.*, 2015) | Unknown - SC |
| Indo-Pacific bottlenose dolphin |  |  |  |
|  | 1452 (934 - 3135) | (Karniski *et al.*, 2018) | Free-ranging - O |
|  | 1277 (1095 - 2190) | (Kogi *et al.*, 2004) | Free-ranging - O |
| Indo-Pacific humpback dolphin |  |  |  |
|  | <1460 | (Zeng *et al.*, 2021) | Free-ranging - O |
|  | <730 (max of 3285) | (Jefferson *et al.*, 2012) | Free-ranging - O |
| Irrawaddy dolphin | 730 | (Tas’an *et al.*, 1980; Marsh *et al.*, 1989) in (Stacey and Arnold, 1999) | Captive - O |
| Killer whale |  |  |  |
|  | 365 - 426 | (Robeck *et al.*, 1993) and references therein | Captive - O |
|  | 365 - 730 | (Kastelein *et al.*, 2003) | Captive - O |
|  | 547 | (Asper *et al.*, 1988) | Captive - O |
| Long-finned pilot whale |  |  |  |
|  | 669 | (Sergeant, 1962) | Harvested - C |
|  | 1241 | (Martin and Rothery, 1993) |  |
|  | 587 | (Betty, 2019) | Stranded - C |
| Narwhal |  |  |  |
|  | 608 | Ohsumi (pers. comm.) in (Braham, 1984) | Unknown |
|  | 441 | (Hay, 1984) | Harvested – SC/S/C |
| Northern bottlenose whale |  |  |  |
|  | 365^e^ | (Christensen, 1973) in (New *et al.*, 2013) | Unclear |
|  | 365 | (Benjaminsen and Christensen, 1979) | Harvested - SC |
|  | 1095 - 1460 | (Feyrer *et al.*, 2020) | Harvested/Stranded - S |
| Pacific white-sided dolphin | 246 - 304 | (Heise, 1996) | Harvested - SC |
| Pantropical spotted dolphin |  |  |  |
|  | 605 | (Myrick *et al.*, 1986) | Bycaught - C |
|  | 273 (730) | (Archer and Robertson, 2004) | Bycaught - SC |
|  | 747 | (Kasuya *et al.*, 1974) | Harvested - C |
| Pygmy sperm whale | 365 - 730 | (Plön, 2004) | Stranded - S |
| Risso’s dolphin |  |  |  |
|  | <730 | (Bloch *et al.*, 2012) | Harvested – S/C |
|  | 365 - 547 | (Amano and Miyazaki, 2004) | Harvested – S/C |
|  | 397 (male)  660 (female) | (Evacitas *et al.*, 2017) | Stranded/bycaught - S |
| Short-finned pilot whale |  |  |  |
|  | 1277 - 2007 | (Kasuya and Marsh, 1984) | Harvested – S/C/SC |
|  | 730 - 1015 | (Kasuya and Tai, 1993) | Harvested - C |
| Sperm whale |  |  |  |
|  | 395 | (Clarke, 1956) | Harvested – SC/S |
|  | 730 - 760 | (Best, 1968) in (Clarke *et al.*, 2011) | Unknown |
|  | 304 - 334 | (Chuzhakina, 1961) in (Clarke *et al.*, 2011) | Unknown |
|  | 730 - 760 | (Ohsumi, 1965) | Harvested – S/O |
|  | 577 | (Clarke *et al.*, 2011) | Free-ranging - O |
|  | 584 - 1270 (max of 4745) | (Best *et al.*, 1984) | Harvested - C |
| Spinner dolphin |  |  |  |
|  | 561 | (Larese and Chivers, 2009) | Bycaught - C |
|  | 532 (398 - 903) | (Perrin *et al.*, 1977) | Bycaught - C |
|  | 307 (285 - 322) | (Perrin *et al.*, 1977) | Bycaught – C |
| Striped dolphin |  |  |  |
|  | 547 (1095) | (Miyazaki, 1977) | Harvested – C/SC |
|  | 487 | (Calzada *et al.*, 1996) | Stranded - C |
| Tucuxi | 213^f^ | (Best and da Silva, 1984) in (Rosas *et al.*, 2010) | Unclear |

^a^Most whales weaned in their second year of life

^b^*Tursiops* sp.

^c^Based on stomach contents from Miller *et al.* (2013) from a single calf with milk and food in its stomach

^d^Maximum estimate

^e^Did not find mention of lactation length, but they do discuss a 12-month gestation period and a nearly 1:1 ratio of pregnant:lactating females, which may be where this value came from

^f^Did not find mention of lactation length in original reference

**Table S19**. Lactation duration of phocids. We noted when values appeared to be based on limited data (denoted as tentative or potentially tentative estimate).

| Species | Duration (days) | Reference |
| --- | --- | --- |
| Baikal seal | 45 - 90^a^ | (references in Thomas *et al.*, 1982) |
| Bearded seal | 23.5 | (Gjertz *et al.*, 2000) |
| Caspian seal | 21.0 - 28.0^a^ | (Wilson *et al.*, 2016) |
| Crabeater seal | 17^a^ | (Shaughnessy and Kerry, 1989) |
| Gray seal | 14.9 - 17.3 | (Baker *et al.*, 1995; Lang *et al.*, 2009) |
| Harbor seal | 19.6 | (Arso Civil *et al.*, 2021) |
| Harp seal | 12.5 | (Kovacs and Lavigne, 1985) |
| Hawaiian monk seal | 36.4 - 39.0 | (Johanos *et al.*, 1994) |
| Hooded seal | 4.0 | (Bowen *et al.*, 1985) |
| Leopard seal | see^b^ | (Southwell *et al.*, 2003) |
| Mediterranean monk seal | 119.4 | (Aguilar *et al.*, 2007) |
| Northern elephant seal | 26.0 | (McDonald and Crocker, 2006) |
| Ribbon seal | 21.0 - 28.0^c^ | (Boveng *et al.*, 2013) |
| Ringed seal | 39.0 | (Hammill *et al.*, 1991) |
| Ross seal | See^d^ | (Southwell *et al.*, 2003) |
| Southern elephant seal | 23.6 | (Fedak *et al.*, 1996) |
| Spotted seal | 18.0 - 28.0^e^ | (Burns, 1973; Zhang *et al.*, 2014) |
| Weddell seal | 33.0 - 39.8 | (Wheatley *et al.*, 2006) |

^a^Tentative estimate

^b^Speculative estimates of 2 – 4 weeks. See Southwell *et al.* (2003) for discussion as well as Kienle *et al.* (2022)

^c^Potentially tentative estimate

^d^Uncited reports of 4 – 6 weeks. See Southwell *et al.* (2003) for discussion

^e^Unclear how upper estimate was determined

**Table S20**. Lactation duration of otariids and odobenids. When available, values represent mean lactation duration or a range of mean values; when not available, they represent point estimates or the typical range of lactation duration observed in a species. Common subspecies from different geographic regions are shown separately, with the species name listed in parentheses.

| Species | Duration (days) | Reference |
| --- | --- | --- |
| Antarctic fur seal | 111.5 - 120.2 | (Lunn and Arnould, 1997) |
| Australian (Cape) fur seal | 313 | (Arnould and Hindell, 2001) |
| Australian sea lion | 365 – 547 | (Higgins and Gass, 1993; Lowther and Goldsworthy, 2016) |
| California sea lion | 304 - 334 | (Harris, 2016) |
| Cape fur seal | 273 - 334 | (David and Rand, 1986) and references therein |
| Galapagos fur seal | 547 - 1,095 | (Trillmich, 1986) |
| Galapagos sea lion | 365 - 1,095 | (Trillmich and Wolf, 2008) |
| Guadalupe (Juan Fernandez) fur seal | 273 - 304 | (Gallo-Reynoso and Figueroa-Carranza, 2010) |
| Juan Fernandez fur seal | >212 and <334^a^ | (Francis *et al.*, 1998) |
| New Zealand fur seal | 285 | (Goldsworthy, 2006) |
| New Zealand sea lion | 273 - 334^a^ | (Gales and Mattlin, 1997) |
| Northern fur seal | 118.9 - 127.0 | (Goebel, 2002; McHuron *et al.*, 2020) |
| South American fur seal | 243 - 365^b^ | (Vaz-Ferreria and Ponce de Leon, 1987 and refereince therein) |
| South American sea lion | 304 - 365^a^ | (Ponce de Leon and Pin, 2006) |
| Steller sea lion | 365 - 730 | (Hastings *et al.*, 2021) |
| Subantarctic fur seal | 300 | (Kerley, 1985) |
| Walrus | 365 - 730^a,c^ | (Fay, 1982) |

^a^Not well resolved

^b^Most weaned in 8^th^ month

^c^Some may nurse up to three years

**Table S21**. Lactation duration of mustelids, ursids, and sirenians. Ranges represent variation of point estimates. We noted when values appeared to be based on limited data (denoted as tentative estimate).

| Species | Duration (days) | Reference |
| --- | --- | --- |
| West Indian manatee | 441 - 730^a^ | (Reid *et al.*, 1995) |
| Marine otter | 243 - 304 | (references in Valqui, 2012) |
| Sea otter | 182 | (Jameson and Johnson, 1993) |
| Polar bear | 547 - 1,022^b^ | (Ramsay and Stirling, 1988; Derocher *et al.*, 1993) |

^a^Tentative estimate

^b^Not well resolved

**Table S22**. Age at which prey supplementation has been documented in odontocete calves.

| Species | Age (months) | Reference |
| --- | --- | --- |
| Amazon river dolphin | 5 | (Boede *et al.*, 2018) |
| Beluga whale | 12 | (Sergeant, 1973; Leung *et al.*, 2010) |
| Bottlenose dolphin | 6 – 18 | (Cockcroft and Ross, 1990a; Peddemors *et al.*, 1992; Reddy *et al.*, 1993; Kastelein *et al.*, 2002) |
| Commerson’s dolphin | 2.4 - 7.8 | (Joseph *et al.*, 1987; Kastelein *et al.*, 1993) |
| Common dolphin | 3 – 6 | (Brophy *et al.*, 2009) |
| Franciscana | 2.4 | (Denuncio *et al.*, 2013) |
| Harbor porpoise | 4^a^ – 5 | (Mohl-Hansen, 1954 in Smith and Read, 1992; Smith and Read, 1992) |
| Irrawaddy dolphin | 6 | (Tas’an *et al.*, 1980) in (Stacey and Arnold, 1999) |
| Killer whale | 2 – 6 | (Asper *et al.*, 1988; Kastelein *et al.*, 2003) |
| Long-finned pilot whale | 6 – 9 | (Sergeant, 1962) |
| Narrow-ridged finless porpoise | 3.2 | (Xian *et al.*, 2012) |
| Pacific white-sided dolphin | 0.7 - 4.1 | (Dalton *et al.*, 1995, 2005) |
| Pantropical spotted dolphin | 3 – 6 | (Kasuya *et al.*, 1974) |
| Short-finned pilot whale | 6 – 12 | (Kasuya and Marsh, 1984) |
| Sperm whale | 8 – 10 | (Best *et al.*, 1984; Tønnesen *et al.*, 2018) |
| Striped dolphin | 3 | (Miyazaki, 1977) |

^a^Age is approximate and given by Lockyer (2003)

**References**

Ackman RG, Eaton CA, Mitchell ED (1971) The bottle-nosed dolphin *Tursiops truncatus*: fatty acid composition of milk triglycerides. *Can J Biochem* 49: 1172–1174.

Adams TC, Bradley D, Brandon EAA, Calkins DG, Castellini MA, Davis RW, Fadely BS, Milette LL, Mcallister DC, Pendleton GW, *et al.* (1996) Alaska Department of Fish and Game Division of Wildlife Conservation Wildlife Technical Bulletin No. 13 May 1996 1992–1994.

Adulyanukosol K, Prasittipornkul C, Man-Anansap S, Boukaew P (2009) Stranding Records of Dugong (*Dugong Dugon*) in Thailand. Proceedings of the 4th International Symposium on SEASTAR2000 and Asian Bio-Logging Science (The 8th SEASTAR2000 Workshop).

Agbayani S, Fortune SME, Trites AW (2020) Growth and development of North Pacific gray whales (*Eschrichtius robustus*). *J Mammal* 101: 742–754.

Aguilar A (1983) Organochlorine pollution in sperm whales, *Physeter macrocephalus*, from the temperate waters of the eastern North Atlantic. *Mar Pollut Bull* 14: 349–352.

Aguilar A (1991) Calving and early mortality in the western Mediterranean striped dolphin, *Stenella coeruleoalba*. *Can J Zool* 69: 1408–1412.

Aguilar A, Cappozzo LH, Gazo M, Pastor T, Forcada J, Grau E (2007) Lactation and mother-pup behaviour in the Mediterranean monk seal *Monachus monachus*: An unusual pattern for a phocid. *J Mar Biol Assoc United Kingdom* 87: 93–99.

Allen AH (1886) Note on the fat of porpoise milk. *Analyst* 190.

Amano M, Miyazaki N (2004) Composition of a school of Risso’s dolphins, *Grampus griseus*. *Mar Mammal Sci* 20: 152–160.

Amano M, Miyazaki N, Petrov EA (2000) Age determination and growth of Baikal seals (*Phoca sibirica*). *Adv Ecol Res* 31: 449–462.

Amano M, Miyazaki N, Yanagisawa F (1996) Life history of Fraser’s dolphin, *Lagenodelphis hosei*, based on a school captured off the Pacific coast of Japan. *Mar Mammal Sci* 12: 199–214.

Amano M, Yamada TK, Kuramochi T, Hayano A, Kazumi A, Sakai T (2014) Life history and group composition of melon-headed whales based on mass strandings in Japan. *Mar Mammal Sci* 30: 480–493.

Amaral RS, Silva VMF da, Rosas FCW (2010) Body weight/length relationship and mass estimation using morphometric measurements in Amazonian manatees *Trichechus inunguis* (Mammalia: Sirenia). *Mar Biodivers Rec* 3: 1–4.

Anabella SF, María Florencia G, Aníbal GN, Alberto CE, Laura DS (2017) Reproductive parameters of female long- finned pilot whales (*Globicephala melas edwardii*) from the Southwestern Atlantic. *Zool Stud* 56: e39.

Andersen M, Hjelset AM, Gjertz I, Lydersen C, Gulliksen B (1999) Growth, age at sexual maturity and condition in bearded seals (*Erignathus barbatus*) from Svalbard, Norway. *Polar Biol* 21: 179–185.

Archer FI, Robertson KM (2004) Age and length at weaning and development of diet of pantropical spotted dolphins, *Stenella attenuata*, from the eastern tropical Pacific. *Mar Mammal Sci* 20: 232–245.

Arnbom T, Fedak MA, Boyd IL (1997) Factors affecting maternal expenditure in southern elephant seals during lactation. *Ecology* 78: 471–483.

Arnold PW, Heinsohn GH (1996) Phylogenetic status of the Irrawaddy dolphin *Orcaella brevirostris* (Owen in Gray): a cladistic analyses. *Mem Queensl Museum* 39: 141–204.

Arnould J, Boyd IL (1995) Temporal patterns of milk production in Antarctic fur seals (*Arctocephalus gazella*). *J Zool* 237: 1–12.

Arnould JP, Hindell MA (1999) The composition of Australian fur seal (*Arctocephalus pusillus doriferus*) milk throughout lactation. *Physiol Biochem Zool* 72: 605–612.

Arnould JPY, Hindell MA (2001) Dive behaviour, foraging locations, and maternal-attendance patterns of Australian fur seals (*Arctocephalus pusillus doriferus*). *Can J Zool* 79: 35–48.

Arnould JPY, Hindell MA (2002) Milk consumption, body composition and pre-weaning growth rates of Australian fur seal (*Arctocephalus pusillus doriferus*) pups. *J Zool* 256: 351–359.

Arnould JPY, Luque SP, Guinet C, Costa DP, Kingston J, Shaffer SA (2003) The comparative energetics and growth strategies of sympatric Antarctic and subantarctic fur seal pups at Îles Crozet. *J Exp Biol* 206: 4497–4506.

Arnould JPY, Ramsay MA (1994) Milk production and milk consumption in polar bears during the ice-free period in western Hudson Bay. *Can J Zool* 72: 1365–1370.

Arnould JPY, Warneke RM (2002) Growth and condition in Australian fur seals (*Arctocephalus pusillus doriferus*) (Carnivora: Pinnipedia). *Aust J Zool* 50: 53–66.

Arso Civil M, Hague E, Langley I, Scott-Hayward L (2021) Allo-suckling occurrence and its effect on lactation and nursing duration in harbour seals (*Phoca vitulina*) in Orkney, Scotland. *Behav Ecol Sociobiol* 75: 121.

Asper ED, Young WG, Walsh MT (1988) Observations on the birth and development of a captive‐born killer whale *Orcinus orca*. *Int Zoo Yearb* 27: 295–304.

Authier M, Cam E, Guinet C (2011) Selection for increased body length in Subantarctic fur seals on Amsterdam Island. *J Evol Biol* 24: 607–616.

Auttila M, Kurkilahti M, Niemi M, Levänen R, Sipilä T, Isomursu M, Koskela J, Kunnasranta M (2016) Morphometrics, body condition, and growth of the ringed seal (*Pusa hispida saimensis*) in Lake Saimaa: Implications for conservation. *Mar Mammal Sci* 32: 252–267.

Bachman KC, Irvine AB (1979) Composition of milk from the Florida manatee, *Trichechus manatus latirostris*. *Comp Biochem Physiol -- Part A Physiol* 62: 873–878.

Backhaus (1904) Blue whale, *Balaenoptera sibbaldi*. *Molkerei Zeit* 14: 481.

Baker C, Perry A, Herman L (1987) Reproductive histories of female humpback whales *Megaptera novaeangliae* in the North Pacific. *Mar Ecol Prog Ser* 41: 103–114.

Baker I, O’Brien J, McHugh K, Berrow S (2018) Female reproductive parameters and population demographics of bottlenose dolphins (*Tursiops truncatus*) in the Shannon Estuary, Ireland. *Mar Biol* 165: 1–19.

Baker JD, Johanos TC, Wurth TA, Littnan CL (2014) Body growth in Hawaiian monk seals. *Mar Mammal Sci* 30: 259–271.

Baker SR, Barrette C, Hammill MO (1995) Mass transfer during lactation of an ice-breeding pinniped, the grey seal (*Halichoerus grypus*), in Nova Scotia, Canada. *J Zool* 236: 531–542.

Baranov EA, Elagin OK, Petrov EA, Shoshenko KA (2001) Oxygen requirement and the affecting factors in the Baikal seal *Pusa sibirica*. *J Evol Biochem Physiol* 37: 648–655.

Barendse J, Best PB, Carvalho I, Pomilla C (2013) Mother knows best: Occurrence and associations of resighted humpback whales suggest maternally derived fidelity to a Southern Hemisphere coastal feeding ground. *PLoS One* 8: e81238.

Barreto AS, Rosas FCW (2006) Comparative growth analysis of two populations of *Pontoporia blainvillei* on the Brazilian coast. *Mar Mammal Sci* 22: 644–653.

Baylis AMM, Nichols PD (2009) Milk fatty acids predict the foraging locations of the New Zealand fur seal: continental shelf versus oceanic waters. *Mar Ecol Prog Ser* 380: 271–286.

Bejarano AC, Wells RS, Costa DP (2017) Development of a bioenergetic model for estimating energy requirements and prey biomass consumption of the bottlenose dolphin *Tursiops truncatus*. *Ecol Modell* 356: 162–172.

Bell CM, Burton HR, Lea MA, Hindell MA (2005) Growth of female southern elephant seals *Mirounga leonina* at Macquarie Island. *Polar Biol* 28: 395–401.

Benjaminsen T (1972) On the biology of the bottlenose whale, *Hyperodon ampullatus* (Forster). *Nor J Zool* 20: 233–241.

Benjaminsen T (1973) Age determination and the growth and age distribution from cementum growth layers of bearded seals at Svalbard. *FiskDir Skr Ser HavUnders* 16: 159–170.

Benjaminsen T, Christensen I (1979) The natural history of the bottlenose whale, *Hyperoodon ampullatus* (Forster). In: Winn HE, Olla BL, eds. Behavior of Marine Animals. Springer, pp 143–164.

Berzin A (1971) The Sperm Whale. Pishchevaya Promyshlennost Moscow [Translated by Israel Program for Scientific Translations].

Best P, Canham P, MacLeod N (1984) Patterns of reproduction in sperm whales, *Physeter macrocephalus*. *Rep Int Whal Comimssion Spec Issue* 6: 51–79.

Best P, Meyer M, Lockyer C (2010) Killer whales in South African waters - a review of their biology. *African J Mar Sci* 32: 171–186.

Best PB (1960) Further information on Bryde’s whale (*Balaenoptera edeni* Anderson) from Saldanha Bay, South Africa. *Nor Hvalfangst-Tid* 49: 201–215.

Best PB (1968) The sperm whale (*Physeter catodon*) off the west coast of South Africa. 2. Reproduction in the female. *Investl Rep Div Sea Fish S Afr*.

Best PB (1982) Seasonal abundance, feeding, reproduction, age and growth in minke whales off Durban (with incidental observations from the Antarctic. *Rep Int Whal Comm* 32: 759–786.

Best PB (1994) Seasonality of reproduction and the length of gestation in southern right whales *Eubalaena australis*. *J Zool* 232: 175–189.

Best PB (2001) Distribution and population separation of Bryde’s whale *Balaenoptera edeni* off southern Africa. *Mar Ecol Prog Ser* 220: 277–289.

Best RC, da Silva VMF (1984) Preliminary analysis of reproductive parameters of the bouto, *Inia geoffrensis*, and the tucuxi, *Sotalia fluviatilis*, in the Amazaon River system. *Rep Int Whal Comm Spec Issue* 6: 361–369.

Bester MN, Van Jaarsveld AS (1994) Sex-specific and latitudinal variance in postnatal growth of the subantarctic fur seal (*Arctocephalus tropicalis*). *Can J Zool* 72: 1126–1133.

Betty EL (2019) Life History of the Long-Finned Pilot Whale (*Globicephala Melas Edwardii*); Insights from Strandings on the New Zealand Coast. Auckland University of Technology.

Betty EL, Stockin KA, Hinton B, Bollard BA, Smith ANH, Orams MB, Murphy S (2022) Age, growth, and sexual dimorphism of the Southern Hemisphere long-finned pilot whale (*Globicephala melas edwardii*). *J Mammal* 103: 560–575.

Bigg M (1982) An assessment of killer whale (*Orcinus orca*) stocks off Vancouver Island, British Columbia. *Rep Int Whal Comm* 36: 655–666.

Bishop A (2014) Age, Growth, Reproduction and Sexual Dimorphism of the Striped Dolphin, *Stenella Coeruleoalba*, off the South-East Coast of Southern Africa. Rhodes University.

Bloch D, Desportes G, Harvey P, Lockyer C, Mikkelsen B (2012) Life history of Risso’s dolphin (*Grampus griseus*) (G. Cuvier, 1812) in the Faroe Islands. *Aquat Mamm* 38: 250–266.

Bloch D, Desportes G, Zachariassen M, Christensen I (1996) The northern bottlenose whale in the Faroe Islands, 1584-1993. *J Zool* 239: 123–140.

Bloch D, Lockyer C, Zachariassen M (1993) Age and growth parameters of the long-finned pilot whale off the Faroe Islands. *Rep Int Whal Comm* 163–207.

Boede EO, Mujica-Jorquera E, Boede F, Varela C (2018) Reproductive management of the Orinoco river dolphin *Inia geoffrensis humboldtiana* in Venezuela. *Int Zoo Yearb* 52: 245–257.

Boltnev AI, York AE, Antonelis GA (1998) Northern fur seal young: interrelationships among birth size, growth, and survival. *Can J Zool* 76: 843–854.

Borges JCG, Da Baviagem Freire AC, Attademo FLN, De Lima Serrano I, Anzolin DG, De Carvalho PSM, Vergara-Parente JE (2012) Growth pattern differences of captive born antillean manatee (*Trichechus manatus*) calves and those rescued in the brazilian northeastern coast. *J Zoo Wildl Med* 43: 494–500.

Börjesson P, Read AJ (2003) Variation in timing of conception between populations of the harbor porpoise. *J Mammal* 84: 948–955.

Botta S, Secchi ER, Muelbert MMC, Danilewicz D, Negri MF, Cappozzo HL, Hohn AA (2010) Age and growth of franciscana dolphins, *Pontoporia blainvillei* (Cetacea: Pontoporiidae) incidentally caught off southern Brazil and northern Argentina. *J Mar Biol Assoc United Kingdom* 90: 1493–1500.

Boveng PL, Bengtson JL, Buckley TW, Cameron MF, Dahle SP, Kelly BP, Megrey BA, Overland JE, Williamson NJ (2009) Status review of the spotted seal (*Phoca largha*). *NOAA Tech Memo NMFS-AFSC* 200: 1–153.

Boveng PL, Bengtson JL, Buckley TW, Cameron MF, Dahle SP, Megrey BA, Overland JE, Williamson NJ (2013) Status review of the ribbon seal (*Histriophoca fasciata*). *NOAA Tech Memo NMFS-AFSC* 191: 1–115.

Bowen WD, Oftedal OT, Boness DJ (1985) Birth to weaning in 4 days: remarkable growth in the hooded seal, *Cystophora cristata*. *Can J Zool* 63: 2841–2846.

Bowen WD, Oftedal OT, Boness DJ (1992) Mass and energy transfer during lactation in a small phocid, the harbor seal (*Phoca vitulina*). *Physiol Zool* 65: 844–866.

Boy CC, Dellabianca N, Goodall RNP, Schiavini ACM (2011) Age and growth in Peale’s dolphin (*Lagenorhynchus australis*) in subantarctic waters off southern South America. *Mamm Biol* 76: 634–639.

Boyd I, McCann T (1989) Pre-natal investment in reproduction by female Antarctic fur seals. *Behav Ecol Sociobiol* 24: 377–385.

Boye TK, Garde E, Nielsen J, Hedeholm R, Olsen J, Simon M (2020) Estimating the age of West Greenland humpback whales through aspartic acid racemization and eye lens bomb radiocarbon methods. *Front Mar Sci* 6: 811.

Braham HW (1984) Review of reproduction in the white whale, *Delphinapterus leucas*, narwhal, *Monodon monoceros*, and Irrawaddy dolphin, *Orcaella brevirostris*, with comments on stock assessment. *Rep Int Whal Comm Spec Issue* 6: 81–90.

Branch T (2008) Biological Parameters for Pygmy Blue Whales. 60th Annual Meeting the International Whaling Commission. Santiago. SC/60/SH6.

Braulik GT, I. Archer F, Khan U, Imran M, Sinha RK, Jefferson TA, Donovan C, Graves JA (2021) Taxonomic revision of the South Asian River dolphins (Platanista): Indus and Ganges River dolphins are separate species. *Mar Mammal Sci* 37: 1022–1059.

Brodie PF (1971) A reconsideration of aspects of growth, reproduction, and behavior of the white whale (*Delphinapterus leucas*), with reference to the Cumberland Sound, Baffin Island, population. *J Fish Res Board Canada* 28: 1309–1318.

Brophy J, Murphy S, Rogan E (2009) The Diet and Feeding Ecology of the Short-Beaked Common Dolphin (*Delphinus Delphis*) in the Northeast Atlantic. International Whaling Commission Scientific Committee Paper SC/61/SM14 18.

Brown KG (1952) Observations on the newly born leopard seal. *Nature* 4336: 9820983.

Bryden M, Smith M, Tedman R, Featherston D (1984) Growth of the Weddell seal, *Leptonychotes weddelli* (Pinnipedia). *Aust J Zool* 32: 33–41.

Bryden MM, Harrison RJ, Lear RJ (1977) Some aspects of the biology of *Peponocephala electra* (Cetacea: Delphinidae) I. General and eeproductive biology. *Aust J Mar Freshw Res* 28: 703–715.

Burnell SR (2001) Aspects of the reproductive biology movements and site fidelity of right whales off Australia. *J Cetacean Res Manag* Special Is: 89–102.

Burns JJ (1970) Remarks on the distribution and natural history of pagophilic pinnipeds in the Bering and Chukchi Seas. *J Mammal* 51: 445–454.

Burns JJ (1973) Marine Mammal Report. Volume XIII Project Progress Report. Alaska Department of Fish and Game.

Burns JJ, Frost KJ (1979) The natural history and ecology of the bearded seal (*Erignathus barbatus*). Final Report. Outer Continental Shelf Environmental Assessment Program.

Burns JJ, Gol’tsev VN (1984) Comparative biology of harbor seals, *Phoca vitulina* Linnaeus, 1758, of the Bommander, Aleutian, and Pribilof Islands. NOAA Technical Report NMFS 12.

Burns JJ, Seaman GA (1986) Investigations of belukha whales in coastal waters of western and northern Alaska. II. Biology and Ecology. of Commerce, NOAA, OCSEAP Final Report 136.

Calzada N, Aguilar A, Lockyer C, Grau E (1997) Patterns of growth and physical maturity in the western Mediterranean striped dolphin, *Stenella coeruleoalba* (Cetacea: Odontoceti). *Can J Zool* 75: 632–637.

Calzada N, Aguilar A, Sorensen T, Lockyer C (1996) Reproductive biology of female striped dolphin (*Stenella coeruleoalba*) from the western Mediterranean. *J Zool* 240: 581–591.

Caon G, Secchi ER, Capp E, Kucharski LC (2008) Milk composition of franciscana dolphin (*Pontoporia blainvillei*) from Rio Grande do Sul, southern Brazil. *J Mar Biol Assoc United Kingdom* 88: 1099–1101.

Cappozzo HL, Campagna C, Monserrat J (1991) Sexual dimorphism in newborn southern sea lions. *Mar Mammal Sci* 7: 385–394.

Carlini AR, Márquez MEI, Soave G, Vergani DF, de Ferrer PAR (1994) Southern elephant seal, *Mirounga leonina*: composition of milk during lactation. *Polar Biol* 14: 37–42.

Carter AM, Miglino MA, Ambrosio CE, Santos TC, Rosas FCW, Lazzarini SM, Carvalho AF (2008) Placentation in the Amazonian manatee (*Trichechus inunguis*). *Reprod Fertil Dev* 20: 537–545.

Chabot D, Stenson GB (2002) Growth and seasonal fluctuations in size and condition of male Northwest Atlantic harp seals *Phoca groenlandica*: An analysis using sequential growth curves. *Mar Ecol Prog Ser* 227: 25–42.

Chambellant M, Stirling I, Gough WA, Ferguson SH (2012) Temporal variations in Hudson Bay ringed seal (*Phoca hispida*) life-history parameters in relation to environment. *J Mammal* 93: 267–281.

Chen I, Watson A, Chou LS (2011) Insights from life history traits of Risso’s dolphins (*Grampus griseus*) in Taiwanese waters: Shorter body length characterizes northwest Pacific population. *Mar Mammal Sci* 27: 43–64.

Chen P, Liu R (1989) Captive husbandry of the Baiji, *Lipotes vexillifer*. In: Perrin WF, Brownell-Jr. RL, Zhou K, Liu J, eds. Biology and Conservation of the River Dolphins: Proceedings of the Workshop on Biology and Conservation of the Platanistoid Dolphin Held at Wuhan, People’s Republic of China, October 28-30, 1986. pp 146–149.

Chen P, Liu R, Lin K (1984) Reproduction and the reproductive system in the beiji, *Lipotes vexillifer*. In: Reproduction in Whales, Dolphins and Porpoises. Proc. Conference, La Jolla, CA, 1981. p 495.

Cherdsukjai P, Buddhachat K, Brown J, Kaewkool M, Poommouang A, Kaewmong P, Kittiwattanawong K, Nganvongpanit K (2020) Age relationships with telomere length, body weight and body length in wild dugong (*Dugong dugon*). *PeerJ* 8: 1–18.

Childerhouse SJ, Dawson SJ, Fletcher DJ, Slooten E, Chilvers BL (2010) Growth and reproduction of female New Zealand sea lions. *J Mammal* 91: 165–176.

Chilvers BL, Robertson BC, Wilkinson IS, Duignan PJ (2007) Growth and survival of New Zealand sea lions, *Phocarctos hookeri*: birth to 3 months. *Polar Biol* 30: 459–469.

Chittleborough R (1958) The breeding cycle of the female humpback whale, *Megaptera nodosa* (Bonnaterre). *Mar Freshw Res* 9: 1–18.

Chivers SJ, Perryman WL, Lynn MS (2019) Reproduction and morphology of Central American and Tres Marias spinner dolphins in the eastern tropical Pacific. *Mar Mammal Sci* 35: 210–233.

Christensen I (1973) Age determination, age distribution and growth of bottlenose whales, *Hyperoodon ampullatus* (Forster), in the Labrador Sea. *Nor J Zool* 21: 331–340.

Christiansen F, Bejder L, Burnell S, Ward R, Charlton C (2022a) Estimating the cost of growth in southern right whales from drone photogrammetry data and long-term sighting histories. *Mar Ecol Prog Ser* 687: 173–194.

Christiansen F, Sironi M, Moore MJ, Di Martino M, Ricciardi M, Warick H, Irschick D, Gutierrez R, Uhart M (2019) Estimating body mass of free-living whales using aeiral photogrammetry and 3D volumetrics. *Methods Ecol Evol* 10: 2034–2044.

Christiansen F, Sprogis KR, Gross J, Castrillon J, Warick HA, Leunissen E, Nash SB (2020) Variation in outer blubber lipid concentration does not reflect morphological body condition in humpback whales. *J Exp Biol* 223. doi:10.1242/jeb.213769

Christiansen F, Uhart M, Bejder L, Ivashchenko Y, Tormosov D, Lewin N, Sironi M (2022b) Foetal growth, birth size and energetic cost of gestation in southern right whales. *J Physiol* 600: 2245–2266.

Chuzhakina E (1961) Morfologichescaya Kharakteristica Yaichnikov Samok Kashalota (*Physeter Catodon*, L., 1758) v Svyazi Opredeleniem Vozrasta. Trudy Instituto Morfologii Zhivotnykh Akademya Nauk SSSR. 34: 33-53. Consulted in Translation NIOT/81 of the National Institut.

Cipriano F (1992) Behavior and Occurrence Patterns, Feeding Ecology, and Life History of Dusky Dolphins (*Lagenorhynchus Obscurus*) off Kaikoura, New Zealand. University of Arizona.

Clark ST, Odell DK, Lacinak CT (2000) Aspects of growth in captive killer whales (*Orcinus orca*). *Mar Mammal Sci* 16: 110–123.

Clarke R (1956) Sperm whales of the Azores. *Discov Reports* 28: 237–298.

Clarke R, Paliza O, Van Vaerebeek K (2011) Sperm whales of the Southeast Pacific. Part VII. Reproduction and grwoth in the female. *Lat Am J Aquat Mamm* 10: 8–39.

Clinton WL (1994) Sexual selection and growth in male northern elephant seals. In: Le Boeuf BJ, Laws RM, eds. Elephant Seals: Population Ecology, Behavior, and Physiology. University of California Press, pp 155–168.

Clowes A. (1929) A note on the composition of whale milk. *Discov Reports* 472–475.

Cockcroft VG (1989) Biological Studies of Bottlenose Dolphins from Natal Coastal Waters. University of Natal.

Cockcroft VG (2002) Natural History of Indopacific Humpback Dolphins (*Sousa Chinensis*) off Southern Africa: International Whaling Commission. Report SC/54/SM22, Shimonoseki, Japan.

Cockcroft VG, Ross GJB (1990a) Observations on the early development of a captive bottlenose dolphin calf. In: Leatherwood S, Reeves RR, eds. The Bottlenose Dolphin. Academic Press, pp 461–478.

Cockcroft VG, Ross GJB (1990b) Age, growth and reproduction in bottlenose dolphins from the east coast of southern Africa. *Fish Bull* 88: 289–302.

Cockcroft VG, Ross GJB (1990c) Biological studies of bottlenose dolphins from Natal coastal waters. In: Leatherwood S, Reeves RR, eds. The Bottlenose Dolphin. Academic Press, pp 461–478.

Costa D, Le Boeuf BJ, Huntley AC, Ortiz C (1986) The energetics of lactation in the northern elephant seal, *Mirounga angustirostris*. *J Zool* 209: 21–33.

Dabin W, Beauplet G, Crespo EA, Guinet C (2004) Age structure, growth, and demographic parameters in breeding-age female subantarctic fur seals, *Arctocephalus tropicalis*. *Can J Zool* 82: 1043–1050.

Dalton L, Robeck TR, Young G (1995) Growth and development of a Pacific white-sided dolphin (*Lagenorhynchus obliquedens*). In: IAAM Conference Proceedings.

Dalton LM, Greger H, Urby M (2005) Growth and development of 10 Pacific white-sided dolphins, (*Lagenorhyncus obliquedens*). In: IAAM Conference Proceedings.

Danil K, Chivers SJ (2007) Growth and reproduction of female short-beaked common dolphins, *Delphinus delphis*, in the eastern tropical Pacific. *Can J Zool* 85: 108–121.

David JHM, Rand RW (1986) Attendance behavior of South African fur seals. In: Gentry RL, Kooyman GL, eds. Fur Seals: Maternal Strategies on Land and at Sea. Princeton University Press, pp 126–141.

Davis R, Adams T, Brandon E, Calkins D, Loughlin T (2002) Female attendance, lactation, and pup growth in Steller sea lions. In: DeMaster D, Atkinson S, eds. Steller Sea Lion Decline: Is It Food II. University of Alaska Sea Grant, AK-SG-02-02, Fairbanks.

Denuncio P, Negri MF, Bastida R, Rodríguez D (2018) Age and growth of Franciscana dolphins from northern Argentina. *J Mar Biol Assoc United Kingdom* 98: 1197–1203.

Denuncio PE, Bastida RO, Danilewicz D, Morón S, Rodríguez-Heredia S, Rodríguez DH (2013) Calf chronology of the Franciscana dolphin (*Pontoporia blainvillei*): Birth, onset of feeding, and duration of lactation in coastal waters of Argentina. *Aquat Mamm* 39: 73–80.

Derocher AE, Andriashek D, Arnould JPY (1993) Aspects of milk composition and lactation in polar bears. *Can J Zool* 71: 561–567.

Derocher AE, Wiig (2002) Postnatal growth in body length and mass of polar bears (*Ursus maritimus*) at Svalbard. *J Zool* 256: 343–349.

Di Beneditto APM, Ramos RMA (2004) Biology of the marine tucuxi dolphin (*Sotalia fluviatilis*) in south-eastern Brazil. *J Mar Biol Assoc United Kingdom* 84: 1245–1250.

Dickie GS, Dawson SM (2003) Age, growth, and reproduction in New Zealand fur seals. *Mar Mammal Sci* 19: 173–185.

Doidge DW (1990a) Age-length and length-weight comparisons in the beluga, *Delphinapterus leucas*. *Can Bull Fish Aquat Sci* 224: 59–68.

Doidge DW (1990b) Age and Stage Based Analysis of the Population Dynamics of Beluga Whales, *Delphinapterus Leucas*, with Particular Reference to the Northern Quebec Population. McGill University.

Donohue MJ, Costa DP, Goebel E, Antonelis GA, Baker JD (2002) Milk intake and energy expenditure of free-ranging northern fur seal, *Callorhinus ursinus*, pups. *Physiol Biochem Zool* 75: 3–18.

Dunkin RC, McLellan WA, Blum JE, Pabst DA (2005) The ontogenetic changes in the thermal properties of blubber from Atlantic bottlenose dolphin *Tursiops truncatus*. *J Exp Biol* 208: 1469–1480.

Eichelberger L, Fetcher ES, Geiling EMK, Vos BJ (1940) The composition of dolphin milk. *J Biol Chem* 134: 171–176.

Eisert R, Oftedal OT, Barrell GK (2013) Milk composition in the Weddell seal *Leptonychotes weddellii*: Evidence for a functional role of milk carbohydrates in pinnipeds. *Physiol Biochem Zool* 86: 159–175.

Ellis SL, Don Bowen W, Boness DJ, Iverson SJ (2000) Maternal effects on offspring mass and stage of development at birth in the harbor seal, *Phoca vitulina*. *J Mammal* 81: 1143–1156.

Evacitas FC, Kao WY, Worthy GAJ, Chou LS (2017) Annual variability in dentin δ^15^N and δ^13^C reveal sex differences in weaning age and feeding habits in Risso’s dolphins (*Grampus griseus*). *Mar Mammal Sci* 33: 748–770.

Evans K, Hindell MA (2004) The age structure and growth of female sperm whales (*Physeter macrocephalus*) in southern Australian waters. *J Zool* 263: 237–250.

Fay FH (1982) Ecology and biology of the Pacific walrus, *Odobenus rosmarus divergens* illiger. *North Am Fauna* 74: 1–279.

Fearnbach H, Durban JW, Ellifrit DK, Balcomb KC (2011) Size and long-term growth trends of endangered fish-eating killer whales. *Endanger Species Res* 13: 173–180.

Fedak M, Arnbom T, Boyd I (1996) The relation between the size of southern elephant seal mothers, the growth of their pups, and the use of maternal energy, fat, and protein during lactation. *Physiol Zool* 69: 887–911.

Ferguson SH, Zhu X, Young BG, Yurkowski DJ, Thiemann GW, Fisk AT, Muir DCG (2018) Geographic variation in ringed seal (*Pusa hispida*) growth rate and body size. *Can J Zool* 96: 649–659.

Ferreira IM, Kasuya T, Marsh H, Best PB (2014) False killer whales (*Pseudorca crassidens*) from Japan and South Africa: Differences in growth and reproduction. *Mar Mammal Sci* 30: 64–84.

Ferrero RC, Walker WA (1993) Growth and reproduction of the northern right whale dolphin, *Lissodelphis borealis*, in the offshore waters of the North Pacific Ocean. *Can J Zool* 71: 2335–2344.

Ferrero RC, Walker WA (1995) Growth and reproduction of the common dolphin, *Delphinus delphis* Linnaeus, in the offshore waters of the North Pacific Ocean. *Fish Bull* 93: 483–494.

Ferrero RC, Walker WA (1996) Age, growth, and reproductive patterns of the Pacific white-sided dolphin (*Lagenorhynchus obliquidens*) taken in high seas drift nets in the central North Pacific Ocean. *Can J Zool* 74: 1673–1687.

Ferrero RC, Walker WA (1999) Age, growth, and reproductive patterns of Dall’s porpoise (*Phocoenoides dalli*) in the central North Pacific Ocean. *Mar Mammal Sci* 15: 273–313.

Feyrer LJ, Zhao ST, Whitehead H, Matthews CJD (2020) Prolonged maternal investment in northern bottlenose whales alters our understanding of beaked whale reproductive life history. *PLoS One* 15: e0235114.

Figueroa-Carranza AL (1994) Early Lactation and Attendance Behavior of the Guadalupe Fur Seal Females, (*Arctocephalus Townsendi*). University of California Santa Cruz.

Fortune SME, Moore MJ, Perryman WL, Trites AW (2021) Body growth of North Atlantic right whales (*Eubalaena glacialis*) revisited. *Mar Mammal Sci* 37: 433–447.

Fortune SME, Trites AW, Perryman WL, Moore MJ, Pettis HM, Lynn MS (2012) Growth and rapid early development of North Atlantic right whales (*Eubalaena glacialis*). *J Mammal* 93: 1342–1354.

Francis J, Boness D, Ochoa-Acuña H (1998) A protracted foraging and attendance cycle in female Juan Fernandez fur seals. *Mar Mammal Sci* 14: 552–574.

Franco-Trecu V (2017) Ecology and conservation status of the South American fur seal in Uraguay. In: Alava JJ, ed. Tropical Pinnipeds: Bio-Ecology, Threats and Conservation. CRC Press.

Frankland PF, Hambly FH (1890) The composition of the milk of the bottle-nose whale (*Globiocephalus melas*). *Chem News J Phys Sci* 61: 63.

Frazer JFD, Huggett ASG (1973) Specific foetal growth rates of cetaceans. *J Zool* 169: 111–126.

Fruet PF, Genoves RC, Möller LM, Botta S, Secchi ER (2015) Using mark-recapture and stranding data to estimate reproductive traits in female bottlenose dolphins (*Tursiops truncatus*) of the Southwestern Atlantic Ocean. *Mar Biol* 162: 661–673.

Galatius A, Jansen OE, Kinze CC (2013) Parameters of growth and reproduction of white-beaked dolphins (*Lagenorhynchus albirostris*) from the North Sea. *Mar Mammal Sci* 29: 348–355.

Gales NJ, Mattlin RH (1997) Summer diving behaviour of lactating New Zealand sea lions, *Phocarctos hookeri*. *Can J Zool* 75: 1695–1706.

Gallo-Reynoso J-P, Figueroa-Carranza A-L (2010) Pup growth of the guadalupe fur seal, *Arctocephalus townsendi*. *Therya* 1: 75–90.

Gálvez C, Pardo MA, Elorriaga-Verplancken FR (2020) Impacts of extreme ocean warming on the early development of a marine top predator: The Guadalupe fur seal. *Prog Oceanogr* 180: 102220.

Gamel CM, Davis RW, David JHM, Meÿer M a., Brandon E (2005) Reproductive energetics and female attendance patterns of Cape fur seals (*Arctocephalus pusillus pusillus*) during early lactation. *Am Midl Nat* 153: 152–170.

Gao A, Zhou K (1992) Sexual dimorphism in the baiji, *Lipotes vexillifer*. *Can J Zool* 70: 1484–1493.

Garde E, Heide-Jørgensen MP, Hansen SH, Nachman G, Forchhammer MC (2007) Age-specific growth and remarkable longevity in narwhals (*Monodon monoceros*) from West Greenland as estimated by aspartic acid racemization. *J Mammal* 88: 49–58.

Garlich-Miller JL, Stewart REA (1998) Growth and sexual dimorphism of Atlantic walruses (*Odobenus rosmarus rosmarus*) in Foxe Basin, Northwest Territories, Canada. *Mar Mammal Sci* 14: 803–818.

Garlich-Miller JL, Stewart REA (1999) Female reproductive patterns and fetal growth of Atlantic walruses (*Odobenus rosmarus rosmarus*) in Foxe Basin, Northwest Territories , Canada. *Mar Mammal Sci* 15: 179–191.

Gauthier JMI, Brochu C, Moore S, Metcalfe CD, Beland P (1998) Environmental contaminants in tissues of a neonate St Lawrence beluga whale (*Delphinapterus leucas*). *Mar Pollut Bull* 36: 102–108.

George JC (2009) Growth, Morphology and Energetics of Bowhead Whales (*Balaena Mysticetus*). University of Alaska Fairbanks.

Georges JY, Groscolas R, Guinet C, Robin JP (2001) Milking strategy in subantarctic fur seals *Arctocephalus tropicalis* breeding on Amsterdam Island: evidence from changes in milk composition. *Physiol Biochem Zool* 74: 548–559.

Gewalt D von W (1978) Unsere tonina (*Inia geoffrensis* Blainvile 1817) - Expedition 1975. *Der Zool Garten* 48: 323–384.

Gjertz I, Kovacs KM, Lydersen C, Wiig (2000) Movements and diving of bearded seal (*Erignathus barbatus*) mothers and pups during lactation and post-weaning. *Polar Biol* 23: 559–566.

Glarou M, Gero S, Frantzis A, Brotons JM, Vivier F, Alexiadou P, Cerdà M, Pirotta E, Christiansen F (2022) Estimating body mass of sperm whales from aerial photographs. *Mar Mammal Sci* 1–23.

Goebel ME (2002) Northern Fur Seal Lactation, Attendance and Reproductive Success in Two Years of Contrasting Oceanography. University of California Santa Cruz.

Gol’din P, Gladilina E (2015) Small dolphins in a small sea: Age, growth and life-history aspects of the black sea common bottlenose dolphin *Tursiops truncatus*. *Aquat Biol* 23: 159–166.

Goldsworthy SD (2006) Maternal strategies of the New Zealand fur seal: Evidence for interannual variability in provisioning and pup growth strategies. *Aust J Zool* 54: 31–44.

Goodall RNP, Schiavini ACM (1995) On the biology of the spectacled porpoise. *Rep Int Whal Comm Spec Issue* 16: 411–454.

Goto M, Ito C, Yahaya MS, Wakamura K, Asano S, Wakai Y, Oka Y, Furuta M, Kataoka T (2004) Effects of age, body size and season on food consumption and digestion of captive dugongs (*Dugong dugon*). *Mar Freshw Behav Physiol* 37: 89–97.

Grandi MF, Crespo EA, Heredia FM, Dellabianca NA (2022) Body growth and reproductive parameters of common dolphins from the southwestern Atlantic 018: 1470–1488.

Grandi MF, Dans SL, García NA, Crespo EA (2010) Growth and age at sexual maturity of South American sea lions. *Mamm Biol* 75: 427–436.

Gregory ME, Kon SK, Rowland SJ, Thompson SY (1955) 578. The composition of the milk of the blue whale. *J Dairy Res* 22: 108–112.

Greig DJ, Gulland FMD, Kreuder C (2005) A decade of live California sea lion (*Zalophus californianus*) strandings along the central California coast: causes and trends, 1991-2000. *Aquat Mamm* 31: 11–22.

Guerrero MA (2018) Body Composition Changes of Hooded Seal (*Cystophora Cristata*) Pups during Extreme Lactation. The Arctic University of Norway.

Guinet C, Georges JY (2000) Growth in pups of the subantarctic fur seal (Arctocephalus tropicalis) on Amsterdam Island. *J Zool* 251: 289–296.

Hale H (1931) Beaked whales-*Hyperoodon planiforms* and *Mesoplodon layarlii*-from south Australia. *Aust Museum* 4: 291–311.

Hamilton PK, Cooper LA (2010) Changes in North Atlantic right whale (*Eubalaena glacialis*) cow-calf association times and use of the calving ground: 1993-2005. *Mar Mammal Sci* 26: 896–916.

Hamilton PK, Marx MK, Kraus SD (1995) Weaning in North Atlantic right whales. *Mar Mammal Sci* 11: 386–390.

Hammill MO, Kingsley MCS, Beck GG, Smith TG (1995) Growth and condition in the northwest Atlantic harp seal. *Can J Fish Aquat Sci* 52: 478–488.

Hammill MO, Lydersen C, Ryg M, Smith TG (1991) Lactation in the ringed seal (*Phoca hispida*). *Can J Fish Aquat Sci* 48: 2471–2476.

Harding KC, Salmon M, Teilmann J, Dietz R, Harkonen T (2018) Population wide decline in somatic growth in harbor seals-early signs of density dependence. *Front Ecol Evol* 6: 259.

Harris JD (2016) Estimation of an Unobservable Transition: From Dependence to Weaning in the California Sea Lion (*Zalophus Californianus*). University of Washington.

Harrison R, Bryden M, McBrearty D (1981) The ovaries and reproduction in *Pontoporia blainvillei* (Cetacea: Platanistidae). *J Zool London* 193: 563–580.

Hastings KK, Johnson DS, Pendleton GW, Fadely BS, Gelatt TS (2021) Investigating life-history traits of Steller sea lions with multistate hidden Markov mark–recapture models: Age at weaning and body size effects. *Ecol Evol* 11: 714–734.

Hauksson E (2007) Growth and reproduction in the Icelandic grey seal. *NAMMCO Sci Publ* 6: 153.

Hay KA (1984) The Life History of the Narwhal (*Monodon Monoceros* L.) in the Eastern Canadian Arctic. McGill University.

Heide-Jørgensen MP, Garde E (2011) Fetal growth of narwhals (*Monodon monoceros*). *Mar Mammal Sci* 27: 659–664.

Heide-Jørgensen MP, Teilmann J (1994) Growth, reproduction, age structure, and feeding habits of white whales (*Delphinapterus leucas*) in West Greenland waters. *Meddelelser om Gr¢nland, Biosci* 39: 195–212.

Heise KA (1996) Life History Parameters of the Pacific White-Sided Dolphin (*Lagenorhynchus Obliquidens*) and Its Diet and Occurrence in the Coastal Waters of British Columbia. University of British Columbia.

Herzing DL (1997) The life history of free-ranging Atlantic spotted dolphins (*Stenella frontalis*): age classes, color phases, and female reproduction. *Mar Mammal Sci* 13: 576–595.

Heyerdahl E. (1930) Om Sammensetningen Av Hvaldyrenes Melk Og Om Forholdene Ved Deres Laktasjon. Publication No. 8. Kommandoer Christian Chrisetensens Hvalfangstmuseum, Sandefjord, Norway.

Higgins L V., Costa DP, Huntley AC, LeBoeuf BJ (1988) Behavioral and physiological measurements of maternal investment in the Steller sea lion, *Eumetopias jubatus*. *Mar Mammal Sci* 4: 44–58.

Higgins L V, Gass L (1993) Birth to weaning: parturition, duration of lactation, and attendance cycles of Australian sea lions (*Neophoca cinerea*). *Can J Zool* 71: 2047–2055.

Hindell M (1994) Early growth and milk-composition in southern elephant seals (*Mirounga leonina*. *Aust J Zool* 42: 723–732.

Hohn A, Read A, Fernandez S, Vidal O, Findley L (1996) Life history of the vaquita. *J Zool London* 239: 235–251.

Hohn AA, Hammond (1985) Early postnatal growth of the spotted dolphin, *Stenella attenuata*, in the offshore eastern tropical Pacific. *Fish Bull* 83: 553–566.

Holser RR, Crocker DE, Robinson PW, Condit R, Costa DP (2021) Density-dependent effects on reproductive output in a capital breeding carnivore, the northern elephant seal (*Mirounga angustirostris*). *Proc R Soc B Biol Sci* 288: 421–425.

Hutchinson E, Atkinson S, Hoover-Miller A (2016) Growth and reproductive tracts from fetal to adult harbor seals in the Gulf of Alaska. *Mar Ecol Prog Ser* 557: 277–288.

Iverson SJ, Bowen W, Boness DJ, Oftedal OT (1993) The effect of maternal size and milk energy output on pup growth in grey seals (*Halichoerus grypus*). *Physiol Zool* 66: 61–88.

Iwasaki T, Kasuya T (1997) Life history and catch bias of Pacific white-sided (*Lagenorhynchus obliquidens*) and northern right whale dolphins (*Lissodelphis borealis*) incidentally taken by the Japanese high seas squid driftnet fishery. *Rep Int Whal Comm* 47: 683–692.

Jameson R, Johnson A (1993) Reproductive characteristics of female sea otters. *Mar Mammal Sci* 9: 156–167.

Jefferson TA (1995) Notes on the biology of the Clymene dolphin (*Stenella clymene*). *Mar Mammal Sci* 11: 564–573.

Jefferson TA, Hung SK, Robertson KM, Archer FI (2012) Life history of the Indo-Pacific humpback dolphin in the Pearl River Estuary, southern China. *Mar Mammal Sci* 28: 84–104.

Jefferson TA, Robertson KM, Wang JY (2002) Growth and reproduction of the finless porpoise in southern China. *Raffles Bull Zool* 105–113.

Jenness R, Odell DK (1978) Composition of milk of the pygmy sperm whale (*Kogia breviceps*). *Comp Biochem Physiol -- Part A Physiol* 61: 383–386.

Jenness R, Sloan R (1970) The composition of milks of various species: a review. *Dairy Sci Abstr* 32: 599–612.

Jenness R, Williams TD, Mullin RJ (1981) Composition of milk of the sea otter (*Enhydra lutris*). *Comp Biochem Physiol Part A Physiol* 70: 375–379.

Johanos TC, Becker BL, Ragen TJ (1994) Annual reproductive cycle of the female Hawaiian monk seal (*Monachus schauinslandi*). *Mar Mammal Sci* 10: 13–30.

Jonsgard A (1951) Studies on the little piked whale. *Nor Hvalfangst-Tid* 40.

Joseph BE, Antrim JE, Cornell LH (1987) Commerson’s dolphin (*Cephalorhynchus commersonii*): A discussion of the first live birth within a marine zoological park. *Zoo Biol* 6: 69–77.

Kajiwara N, Kamikawa S, Amano M, Hayano A, Yamada TK, Miyazaki N, Tanabe S (2008) Polybrominated diphenyl ethers (PBDEs) and organochlorines in melon-headed whales, *Peponocephala electra*, mass stranded along the Japanese coasts: Maternal transfer and temporal trend. *Environ Pollut* 156: 106–114.

Kannan K, Senthilkumar K, Sinha RK (1997) Sources and accumulation of butyltin compounds in Ganges river dolphin, *Platanista gangetica*. *Appl Organomet Chem* 11: 223–230.

Karamanlidis AA, Dendrinos P, de Larrinoa PF, Gücü AC, Johnson WM, Kiraç CO, Pires R (2016) The Mediterranean monk seal *Monachus monachus*: Status, biology, threats, and conservation priorities. *Mamm Rev* 46: 92–105.

Karniski C, Krzyszczyk E, Mann J (2018) Senescence impacts reproduction and maternal investment in bottlenose dolphins. *Proc R Soc B Biol Sci* 285. doi:10.1098/rspb.2018.1123

Kastelein R, McBain J, Neurorhr B (1993) Information on the biology of Commerson’s dolphins (*Cephalorhynchus commersonii*). *Aquat Mamm* 19: 13–19.

Kastelein RA (1994) Food Consumption and Growth of Marine Mammals. University of Wageningen.

Kastelein RA, Kershaw J, Berghout E, Wiepkema PR (2003) Food consumption and suckling in killer whales *Orcinus orca* at Marineland Antibes. *Int Zoo Yearb* 38: 204–218.

Kastelein RA, Mosterd J, Schooneman NM, Wiepkema PR (2000) Food consumption, growth, body dimensions, and respiration rates of captive false killer whales (*Pseudorca crassidens*). *Aquat Mamm* 61: 33–44.

Kastelein RA, Vaughan N, Walton S, Wiepkema PR (2002) Food intake and body measurements of Atlantic bottlenose dolphins (*Tursiops truncatus*) in captivity. *Mar Environ Res* 53: 199–218.

Kasuya T (1972a) Some informations on the growth of the Ganges Dolphin with a comment on the Indus Dolphin. *Sci Reports Whales Res Inst* 24: 87–108.

Kasuya T (1972b) Growth and reproduction of *Stenella caeruleoalba* based on the age determination by means of dentinal growth layers. *Sci Reports Whales Res Inst* 24: 57–79.

Kasuya T (1976) Reconsideration of life history parameters of the spotted and striped dolphins based on cemental layers. *Sci Reports Whales Res Inst* 73–106.

Kasuya T (1977) Age determination and growth of the Baird’s beaked whale with a comment on the fetal growth rate. *Sci Reports Whales Res Inst* 1–20.

Kasuya T (1978) The life history of Dall’s porpoise with special reference to the stock off the Pacific coast of Japan. *Sci Rep from Whales Res Inst* 30: 1–63.

Kasuya T, Brownell-Jr. RL (1979) Age determination, reproduction, and growth of the Franciscana dolphin *Pontoporia blainvillei*. *Sci Reports Whales Res Inst* 31: 45–67.

Kasuya T, Kureha K (1979) The population of finless porpoise *Neophocaena phocaenoides* in the inland Sea of Japan. *Sci Reports Whales Res Inst* 1–44.

Kasuya T, Marsh H (1984) Life history and reproductive biology of the short-finned pilot whale, *Globicephala macrorhynchus*, off the Pacific coast of Japan. *Reports Int Whal Comm Spec Issue* 6: 259–310.

Kasuya T, Matsui S (1984) Age determination and growth of the short-finned pilot whale off the Pacific coast of Japan. *Sci Reports Whales Res Inst* 35: 57–91.

Kasuya T, Miyazaki N, Dawbin WH (1974) Growth and reproduction of Stenella attenuata in the Pacific coast of Japan. *Sci Reports Whales Res Inst Tokyo* 157–226.

Kasuya T, Sergeant DE, Tanaka K (1988) Re-examination of life history parameters of long-finned pilot whales in the Newfoundland waters. *Sci Rep from Whales Res Inst* 39: 103–119.

Kasuya T, Shiraga S (1985) Growth of Dall’s porpoise in the western North Pacific and suggested geographic growth differentiation. *Sci Rep from Whales Res Inst* 139–152.

Kasuya T, Tai S (1993) Life history of short-finned pilot whale stocks off Japan and a discription of the fishery. (*Globicephala sieboldii*). *Rep Int Whal Comm Spec Issue* 439–473.

Kasuya T, Tobayama T, Saiga T, Kataoka T (1986) Perinatal growth of delphinoids information from aquarium reared bottlenose dolphins and finless porpoises. *Sci Reports Whales Res Inst* 85–98.

Kemper C, Talamonti M, Bossley M, Steiner A (2019) Sexual maturity and estimated fecundity in female Indo-Pacific bottlenose dolphins (*Tursiops aduncus*) from South Australia: Combining field observations and postmortem results. *Mar Mammal Sci* 35: 40–57.

Kenny DE, Bickel C (2005) Growth and development of Polar bear *Ursus maritimus* cubs at Denver Zoological Gardens. *Int Zoo Yearb* 39: 205–214.

Kenyon KW (1969) The sea otter in the eastern Pacific Ocean. *North Am Fauna* 68: 1–341.

Kenyon KW, Rice DW (1957) Life history of the Hawaiian monk seal. *Pacific Sci* 8: 215–252.

Kerley GIH (1985) Pup growth in the fur seals *Arctocephalus tropicalis* and *A. gazella* on Marion Island. *J Zool* 205: 315–324.

Kienle SS, Goebel ME, LaBrecque E, Borras-Chavez R, Trumble SJ, Kanatous SB, Crocker DE, Costa DP (2022) Plasticity in the morphometrics and movements of an Antarctic apex predator, the leopard seal. *Front Mar Sci* 9. doi:10.3389/fmars.2022.976019

Kleinenberg S, Yablokov A, Bel’kovich B, Tarasevich M (1969) Beluga: Investigation of the Species. Translated from Russian by Israel Program for Sci Tranl, Jerusalem.

Kogi K, Hishii T, Imamura A, Iwatani T, Dudzinski MK (2004) Indo-Pacific bottlenose dolphins (*Tursiops aduncus*) Around Mikura Island, Japan. *Mar Mammal Sci* 20: 510–526.

Komura H, Hashikata M, Yoshida T, Kanzaki B, Hosono A, Yamada TK (2002) Composition of Stejneger’s beaked whale (*Mesoplodon stejnegeri)* milk and characteristics of milk fat. *Milk Sci* 51: 133–135.

Koski W, Davis R, GW M, Withrow D (1993) Reproduction. In: Burns J, Montague J, Cowles C, eds. The Bowhead Whale. Special Publication Number 2, Society for Marine Mammalogy, pp 239–274.

Kotik C, Durban JW, Barrett-lennard LG (2022) Morphometrics of mammal-eating killer whales from drone photogrammetry, with comparison to sympatric fish-eating killer whales in the eastern North Pacific. *Mar Mammal Sci* 1–17.

Kovacs K, Lavigne D (1986) Growth of grey seal (*Halichoerus grypus*) neonates: differential maternal investment in the sexes. *Can J Zool* 64: 1937–1943.

Kovacs KM, Krafft BA, Lydersen C (2020) Bearded seal (*Erignathus barbatus*) birth mass and pup growth in periods with contrasting ice conditions in Svalbard, Norway. *Mar Mammal Sci* 36: 276–284.

Kovacs KM, Lavigne DM (1985) Neonatal growth and organ allometry of Northwest Atlantic harp seals (*Phoca groenlandica*). *Can J Zool* 63: 2793–2799.

Krafft BA, Kovacs KM, Frie AK, Haug T, Lydersen C (2006) Growth and population parameters of ringed seals (Pusa hispida) from Svalbard, Norway, 2002-2004. *ICES J Mar Sci* 63: 1136–1144.

Kretzmann MB, Costa DP, Higgins L V, Needham D (1991) Milk composition of Australian sea lions, *Neophoca cinerea*: variability in lipid content. *Can J Zool* 69: 2556–2561.

Kretzmann MB, Costa DP, Le Boeuf BJ (1993) Maternal energy investment in elephant seal pups: evidence for sexual equality? *Am Nat* 141: 466–480.

Kriete B (1995) Bioenergetics in the Killer Whale, *Orcinus Orca*. The University of British Columbia.

Laake JL, Melin SR, Orr AJ, Greig DJ, Prager KC, Delong RL, Harris JD (2016) California sea lion sex- and age-specific morphometry. U.S. Dep. Commer., NOAA Tech. Memo. NMFS-AFSC-312 1–21.

Laidre KL, Estes JA, Tinker MT, Bodkin J, Monson D, Schneider K (2006) Patterns of growth and body condition in sea otters from the Aleutian archipelago before and after the recent population decline. *J Anim Ecol* 75: 978–989.

Lang SLC, Iverson SJ, Bowen WD (2005) Individual variation in milk composition over lactation in harbour seals (*Phoca vitulina*) and the potential consequences of intermittent attendance. *Can J Zool* 83: 1525–1531.

Lang SLC, Iverson SJ, Bowen WD (2009) Repeatability in lactation performance and the consequences for maternal reproductive success in gray seals. *Ecology* 90: 2513–2523.

Lang SLC, Iverson SJ, Bowen WD (2011) The influence of reproductive experience on milk energy output and lactation performance in the grey seal (*Halichoerus grypus*). *PLoS One* 6: e19487.

Lanzetti A, Berta A, Ekdale EG (2020) Prenatal development of the humpback whale: growth rate, tooth loss and skull shape changes in an evolutionary framework. *Anat Rec* 303: 180–204.

Larese JP, Chivers SJ (2009) Growth and reproduction of female eastern and whitebelly spinner dolphins incidentally killed in the eastern tropical Pacific tuna purse-seine fishery. *Can J Zool* 87: 537–552.

Lauer B., Baker BE (1969) Whale milk. I. Fin whale (*Balaenoptera physalus*) and beluga whale (*Delphinapterus leucas*) milk: gross composition and fatty acid constitution. *Can J Zool* 47: 95–97.

Lavigne D, Steward R, Fletcher F (1982) Changes in composition and energy content of harp seal milk during lactation. *Physiol Zool* 55: 1–9.

Laws RM, Baird A, Bryden MM (2003a) Breeding season and embryonic diapause in crabeater seals (*Lobodon carcinophagus*). *Reproduction* 126: 365–370.

Laws RM, Baird A, Bryden MM (2003b) Size and growth of the crabeater seal *Lobodon carcinophagus* (Mammalia: Carnivora). *J Zool* 259: 103–108.

Le Boeuf BJ, Ortiz CL (1977) Composition of elephant seal milk. *J Mammal* 58: 683–685.

Learmonth JA, Murphy S, Luque PL, Reid RJ, Patterson IAP, Brownlow A, Ross HM, Barley JP, Santos MB, Pierce GJ (2014) Life history of harbor porpoises (Phocoena phocoena) in Scottish (UK) waters. *Mar Mammal Sci* 30: 1427–1455.

Leung ES, Vergara V, BarLancerett-Lennard LG (2010) Allonursing in captive belugas (*Delphinapterus leucas*). *Zoo Biol* 29: 633–637.

Lima M, Paez E (1995) Growth and reproductive patterns in the South American fur seal. *J Mammal* 76: 1249–1255.

Lockyer C (1976) Body weights of some species of large whales. *ICES J Mar Sci* 36: 259–273.

Lockyer C (1981a) Growth and energy budgets of large baleen whales from the Southern Hemisphere. *Mamm seas, vol 3, (FAO Fish Ser no 5)* 379–487.

Lockyer C (1981b) Estimation of the energy costs of growth, maintenance and reproduction of the female minke whale, (*Balaenoptera acutorostrata*), from the Southern Hemisphere. *Rep Int Whal Comm* 31: 337–343.

Lockyer C (1984) Review of baleen whale (Mysticeti) reproduction and implications for management. *Rep Int Whal Comm Spec Issue* 6: 27–50.

Lockyer C (1991) Body Composition of the Sperm Whale, *Physter Catodon*, with Special Reference to the Possible Functions of Fat Depots. Marine Research Institute.

Lockyer C (1993) Seasonal changes in body fat condition of northeast Atlantic pilot whales, and their biological significance. *Rep Int Whal Comm* 14: 325–350.

Lockyer C (1995) Aspects of the biology of the harbour porpoise, *Phocoena phocoena*, from British waters. In: Blix AS, Walløe L, Ulltang O, eds. Whales, Seals, Fish and Man. Elsevier Science, pp 443–464.

Lockyer C (2003) Harbour porpoises (*Phocoena phocoena*) in the North Atlantic: Biological parameters. *NAMMCO Sci Publ* 5: 71–89.

Lockyer C, Kinze C (2003) Status, ecology and life history of harbour porpoise (*Phocoena phocoena*), in Danish waters. *NAMMCO Sci Publ* 5: 143.

Lowther AD, Goldsworthy SD (2016) When were the weaners weaned? Identifying the onset of Australian sea lion nutritional independence. *J Mammal* 97: 1304–1311.

Lubetkin SC, Zeh JE, George JC (2012) Statistical modeling of baleen and body length at age in bowhead whales (*Balaena mysticetus*). *Can J Zool* 90: 915–931.

Lubetkin SC, Zeh JE, Rosa C, George JC (2008) Age estimation for young bowhead whales (Balaena mysticetus) using annual baleen growth increments. *Can J Zool* 86: 528–538.

Lunn NJ, Arnould JPY (1997) Maternal investment in Antarctic fur seals: Evidence for equality in the sexes? *Behav Ecol Sociobiol* 40: 351–362.

Luque P, Ferguson SH (2010) Age structure, growth, mortality, and density of belugas (*Delphinapterus leucas*) in the Canadian Arctic: responses to environment? *Polar Biol* 33: 163–178.

Lydersen C, Hammill M, Ryg M (1992) Water flux and mass gain during lactation in free-living ringed seal (*Phoca hispida*). *J Zool* 228: 361–369.

Lydersen C, Hammill MO (1993) Activity, milk intake and energy consumption in free-living ringed seal (*Phoca hispida*) pups. *J Comp Physiol B* 163: 433–8.

Lydersen C, Hammill MO, Kovacs KM (1995) Milk intake, growth and energy consumption in pups of ice-breeding grey seals (*Halichoerus grypus*) from the Gulf of St. Lawrence, Canada. *J Comp Physiol B* 164: 585–592.

Lydersen C, Kovacs KM (1996) Energetics of lactation in harp seals (*Phoca groenlandica*) from the Gulf of St. Lawrence, Canada. *J Comp Physiol B* 166: 295–304.

Lydersen C, Kovacs KM, Hammill MO (1997) Energetics during nursing and early postweaning fasting in hooded seal (*Cystophora cristata*) pups from the Gulf of St Lawrence, Canada. *J Comp Physiol - B Biochem Syst Environ Physiol* 167: 81–88.

Lydersen C, Kovacs KM, Hammill MO, Gjertz I (1996) Energy intake and utilisation by nursing bearded seal (*Erignathus barbatus*) pups from Svalbard, Norway. *J Comp Physiol - B Biochem Syst Environ Physiol* 166: 405–411.

M’Intosh (1885) XLVII.— Notes from the St. Andrews Marine Laboratory (under the Fishery Board for Scotland).—No. III . *Ann Mag Nat Hist* 16: 480–487.

Mackintosh N, Wheeler J (1929) Southern blue and fin whales. *Discov Reports* 1: 257–540.

Mann J, Connor RC, Barre LM, Heithaus MR (2000) Female reproductive success in bottlenose dolphins (*Tursiops* sp.): Life history, habitat, provisioning, and group-size effects. *Behav Ecol* 11: 210–219.

Manning L, Grantz K (2017) Endangered Species Act Status Review Report for Hector’s Dolphin (*Cephalorhynchus Hectori*). Report to the National Marine Fisheries Service, Office of Protected Resources, Silver Spring, Maryland.

Markussen NH, Ryg M, Lydersen C (1992) Food consumption of the NE Atlantic minke whale (*Balaenoptera acutorostrata*) population estimated with a simulation model. *ICES J Mar Sci* 49: 317–323.

Marsh H (1980) Age determination of the dugong (*Dugong dugon* (Muller)) in Northern Australia and its biological implications. *Rep Int Whal Comm* 181–201.

Marsh H, Heinsohn G, Marsh L (1984) Breeding cycle, life history and population dynamics of the dugong,*Dugon dugon* (Sirenia: Dugongidae). *Aust J Zool* 32: 767–788.

Marsh H, Lloze R, Heinsohn G, Kasuya T (1989) Irrawaddy dolphin *Orcaella brevirostris* (Gray, 1866). In: Harrison R, Ridgway S, eds. Handbook of Marine Mammals. Academic Press, New York, pp 101–118.

Martin AR, Da Silva VMF (2018) Reproductive parameters of the Amazon river dolphin or boto, *Inia geoffrensis* (Cetacea: Iniidae); an evolutionary outlier bucks no trends. *Biol J Linn Soc* 123: 666–676.

Martin AR, Rothery P (1993) Reproductive parameters of female long-finned pilot whales (*Globicephala melas*) around the Faroe Islands. *Rep Int Whal Comimssion Spec Issue* 14: 263–304.

Masaki Y (1976) Biological studies on the North Pacific sei whale. *Bull Far Seas Fish Res Lab* 14: 1–104.

Matthews CJD, Ferguson SH (2015) Weaning age variation in beluga whales (*Delphinapterus leucas*). *J Mammal* 96: 425–437.

Matthews L (1938) The sei whale, *Balaenoptera borealis*. *Discov Reports* 17: 183–290.

Mattson MC, Mullin KD, Ingram GW, Hoggard W (2006) Age structure and growth of the bottlenose dolphin (*Tursiops truncatus*) from strandings in the Mississippi sound region of the north-central Gulf of Mexico from 1986 to 2003. *Mar Mammal Sci* 22: 654–666.

McDonald BI, Crocker DE (2006) Physiology and behavior influence lactation efficiency in northern elephant seals (*Mirounga angustirostris*). *Physiol Biochem Zool* 79: 484–496.

McDonald BI, Goebel ME, Crocker DE, Costa DP (2012) Dynamic influence of maternal and pup traits on maternal care during lactation in an income breeder, the Antarctic fur seal. *Physiol Biochem Zool* 85: 243–254.

McFee WE, Adams JD, Fair PA, Bossart GD (2012) Age distribution and growth of two bottlenose dolphin (*Tursiops truncatus*) populations from capture-release studies in the Southeastern United States. *Aquat Mamm* 38: 17–30.

McFee WE, Schwacke JH, Stolen MK, Mullin KD, Schwacke LH (2010) Investigation of growth phases for bottlenose dolphins using a Bayesian modeling approach. *Mar Mammal Sci* 26: 67–85.

McHuron EA, Luxa K, Pelland NA, Holsman K, Ream R, Zeppelin T, Sterling JT (2020) Practical application of a bioenergetic model to inform management of a declining fur seal population and their commercially important prey. *Front Mar Sci* 7: 597973.

McIntosh RR, Kennedy CW (2013) Morphology, sex ratio and cause of death in Australian sea lion (*Neophoca cinerea*) pups. *Aust Mammal* 35: 93–100.

McKenzie J, Page B, Goldsworthy SD, Hindell MA (2007) Growth strategies of New Zealand fur seals in southern Australia. *J Zool* 272: 377–389.

McLaren IA (1958) Some aspects of growth and reproduction of the bearded Seal, *Erignathus barbatus* (Erxleben). *J Fish Res Board Canada* 15: 219–227.

McLaren IA (1993) Growth in pinnipeds. *Biol Rev Camb Philos Soc* 68: 1–79.

Mead JG (1984) Survey of reproductive data for the beaked whales (Ziphiidae). *Rep Int Whal Comm Spec Issue* 91–96.

Mead JG, Walker WA, Houck WJ (1982) Biological observations on *Mesoplodon carlhubbsi* (Cetacea, Ziphiidae). *Smithson Contrib to Zool* 344: 1–25.

Mellish JE, Iverson SJ, Bowen WD (1999a) Variation in milk production and lactation performance in grey seals and consequences for pup growth and weaning characteristics. *Physiol Biochem Zool* 72: 677–690.

Mellish JE, Iverson SJ, Bowen WD, Hammill MO (1999b) Fat transfer and energetics during lactation in the hooded seal: The roles of tissue lipoprotein lipase in milk fat secretion and pup blubber deposition. *J Comp Physiol B* 169: 377–390.

Mendoza P, Velasquez J, Sanchez J, Davila L, Loja D, Riveros R, Vilchez C (2019) Growth curve of Amazonian manatee (*Trichechus inunguis*) in captivity. *Aquat Mamm* 45: 389–397.

Miller E, Lalas C, Dawson S, Ratz H, Slooten E (2013) Hector’s dolphin diet: The species, sizes and relative importance of prey eaten by *Cephalorhynchus hectori*, investigated using stomach content analysis. *Mar Mammal Sci* 29: 606–628.

Miyazaki N (1977) Growth and reproduction of *Stenella coeruleoalba* off the Pacific coast of Japan. *Sci Rep from Whales Res Inst* 21–48.

Miyazaki N, Fujise Y, Iwata K (1998) Biological analysis of a mass stranding of melon-headed whales (*Peponocephala electra*) at Aoshima, Japan. *Bull Natl Sci Musuem Tokyo* 24: 31–60.

Mohl-Hansen (1954) Investigations on reproduction and growth of the porpoise (*Phocaena phocaena* (L.)) from the Baltic. *Vidensk Medd Dan Naturhist Foren* 116: 369–396.

Monnett C, Rotterman LM, Siniff DB (1991) Sex-related patterns of postnatal development of sea otters in Prince William Sound, Alaska. *J Mammal* 72: 37–41.

Mueller B, Pörschmann U, Wolf JBW, Trillmich F (2011) Growth under uncertainty: The influence of marine variability on early development of Galapagos sea lions. *Mar Mammal Sci* 27: 350–365.

Murphy S, Spradlin TR, Mackey B, McVee J, Androukaki E, Tounta E, Karamanlidis AA, Dendrinos P, Joseph E, Lockyer C, *et al.* (2012) Age estimation, growth and age-related mortality of Mediterranean monk seals *Monachus monachus*. *Endanger Species Res* 16: 149–163.

Murphy S, Winship A, Dabin W, Jepson PD, Deaville R, Reid RJ, Spurrier C, Rogan E, López A, González AF, *et al.* (2009) Importance of biological parameters in assessing the status of *Delphinus delphis*. *Mar Ecol Prog Ser* 388: 273–291.

Myrick A, Hohn A, Barlow J, Sloan P (1986) Reproductive biology of female spotted dolphins, *Stenella attenuata*, from the eastern tropical Pacific. *Fish Bull* 84.

Naito Y, Nishiwaki M (1972) The growth of two species of the harbour seal in the adjacent waters of Hokkaido. *Sci Reports Whales Res Inst* 24: 127–144.

Nerini MK, Braham HW, Marquette WM, Rugh DK (1984) Life history of the bowhead whale, *Balaena mysticetus* (Mammalia: Cetacea). *J Zool* 204: 443–468.

Neuenhoff RD, Cowan DF, Whitehead H, Marshall CD (2011) Prenatal data impacts common bottlenose dolphin (*Tursiops truncatus*) growth parameters estimated by length-at-age curves. *Mar Mammal Sci* 27: 195–216.

New LF, Moretti DJ, Hooker SK, Costa DP, Simmons SE (2013) Using energetic models to investigate the survival and reproduction of beaked whales (family Ziphiidae). *PLoS One* 8: e68725.

Newby TC (1982) Life History of Dall Porpoise (*Phocoenoides Dalli*, True 1885) Incidentally Taken by the Japanase High Seas Salmon Mothership Fishery in the Northwestern North Pacific and Western Bering Sea, 1978 and 1980. University of Washington.

Nolte Z (2013) The Natural History of the Humpback Dolphin, *Sousa chinensis*, in KwaZulu-Natal, South Africa: Age, Growth and Reproduction. M.Sc. thesis 158 pp.

Ochoa-Acuna H, Francis JM, Boness DJ (1998) Interannual variation in birth mass and postnatal growth rate of Juan Fernández fur seals. *Can J Zool* 76: 978–983.

Ochoa-Acuna H, Francis JM, Oftedal OT (1999) Influence of long intersuckling interval on composition of milk in the Juan Fernandez fur seal, *Arctocephalus philippii*. *J Mammal* 80: 758–767.

Oftedal OT (1997) Lactation in whales and dolphins: Evidence of divergence between baleen- and toothed-species. *J Mammary Gland Biol Neoplasia* 2: 205–230.

Oftedal OT, Boness DJ, Bowen WD (1988) The composition of hooded seal (*Cystophora cristata*) milk: an adaptation for postnatal fattening. *Can J Zool* 66: 318–322.

Oftedal OT, Bowen W., Boness DJ (1993) Energy transfer by lactating hooded seals and nutrient deposition in their pups during the four days from birth to weaning. *Physiol Zool* 66: 412–436.

Oftedal OT, Bowen WD, Boness DJ (1996) Lactation performance and nutrient deposition in pups of the harp seal, *Phoca groenlandica*, on ice floes off southeast Labrador. *Physiol Zool* 69: 635–657.

Oftedal OT, Iverson SJ, Boness DJ (1987) Milk and energy intakes of suckling California sea lion *Zalophus californianus* pups in relation to sex, growth, and predicted maintenance requirements. *Physiol Zool* 5: 560–575.

Ohsumi S (1965) Reproduction of the sperm whale in the north-west Pacific. *Sci Reports Whales Res Inst* 19: 1–35.

Ohsumi S (1966) Allomorphosis between body length at sexual maturity and body length at birth in the Cetacea. *J Mammal Soc Japan* 3: 3–7.

Ohsumi S, Kawasaki M, Nishiwaki S (1994) Biological results of beaked whales surveyed by Japanese whale research programme under special permit in the Antarctic and the need of their research take. Paper submitted to the 46th IWC/SC May (1994).

Ohsumi S, Masaki. Y, Kawamura. A (1970) Stock of the Antarctic minke whale. *Sci Rep Whales Res Inst* 22: 75–125.

Ohsumi S, Masaki Y (1975) Biological parameters of the Antarctic minke whale at the virginal population level. *J Fish Res Board Canada* 32: 995–1004.

Ohsumi S, Nishiwaki M, Hibiya T (1958) Growth of fin whale in the Northern Pacific. *Sci Reports Whales Res Inst* 13: 97–133.

Ohta K, Watarai T, Oishi T, Ueshiba Y, Hirose S, Yoshizawa T, Akikusa Y, Sato M, Okano H (1953) Composition of fin whale milk. *Proc Jpn Acad* 29: 392–398.

Ólafsdóttir D, Víkingsson GA, Halldórsson SD, Sigurjónsson J (2003) Growth and reproduction in harbour porpoises (*Phocoena phocoena*) in Icelandic waters. *NAMMCO Sci Publ* 5: 195.

Omura H, Fujino K, Kimura S (1955) Beaked whale *Berardius bairdi* of Japan, with notes on *Ziphius cavirostris*. *Sci Reports Whales Res Inst* 10: 89–132.

Omura H, Ohsumi S, Nemoto T, Nasu K, Kasuya T (1969) Black right whales in the North Pacific. *Sci Reports Whales Res Inst* 21: 1–66.

Ono KA, Boness DJ, Oftedal OT (1987) The effect of a natural environmental disturbance on maternal investment and pup behavior in the California sea lion. *Behav Ecol Sociobiol* 21: 109–118.

Ortiz CL, Le Boeuf BJ, Costa DP (1984) Milk intake of elephant seal pups: an index of parental investment. *Am Nat* 124: 416–422.

Parsons E, Chan H (1998) Organochlorines in Indo-Pacific hump-backed dolphins (*Sousa chinensis*) and finless porpoise from Hong Kong. In: Morton B, ed. The Marine Biology of the South China Sea. pp 423–427.

Pastukhov V (1993) Nerpa Baikala: Biologicheskie Osnovy Ratsional’nogo Ispol’zpvaniya i Okhrany Resursov (Baikal Seal: Biological Bases of Rational Use and Preservation of Resources). Novosibirsk.

Peddemors VM, de Muelenaere HJH, Devchand K (1989) Comparative milk composition of the bottlenosed dolphin (*Tursiops truncatus*), humpback dolphin (*Sousa plumbea*) and common dolphin (*Delphinus delphis*) from southern African waters. *Comp Biochem Physiol -- Part A Physiol* 94: 639–641.

Peddemors VM, Fothergill M, Cockcroft VG (1992) Feeding and growth in a captive-born bottlenose dolphin *Tursiops truncatus* . *South African J Zool* 27: 74–80.

Pedersen T (1952) A note on humpback oil and on the milk and milk fat from this species (*Megaptera nodosa*). *Nor Hvalfangst-Tid* 41: 375–378.

Pedro S, Boba C, Dietz R, Sonne C, Rosing-Asvid A, Hansen M, Provatas A, McKinney MA (2017) Blubber-depth distribution and bioaccumulation of PCBs and organochlorine pesticides in Arctic-invading killer whales. *Sci Total Environ* 601–602: 237–246.

Perrin WF, Coe JM, Zweifel JR (1976) Growth and Reproduction of the spotted porpoise, *Stenella attenuata* , in the offshore Eastern Tropical Pacific. *Fish Bull* 74: 229–269.

Perrin WF, Dolar MLL, Chan CM, Chivers SJ (2005) Length-weight relationships in the spinner dolphin (*Stenella longirostris*). *Mar Mammal Sci* 21: 765–778.

Perrin WF, Holts DB, Miller RB (1977) Growth and reproduction of the eastern spinner dolphin, a geographical form of *Stenella longirostris*. *Fish Bull* 75: 725–750.

Perrin WF, Reilly SB (1984) Reproductive parameters of dolphins and small whales of the family Delphinidae. *Reprod whales, dolphins porpoises Proc Conf La Jolla, CA, 1981* 97–133.

Pervaiz S, Brew K (1986) Composition of the milks of the bottlenose dolphin (*Tursiops truncatus*) and the florida manatee (*Trichechus manatus latirostris*). *Comp Biochem Physiol -- Part A Physiol* 84: 357–360.

Pilson MEQ, Waller DW (1970) Composition of milk from spotted and spinner porpoises. *J Mammal* 51: 74–79.

Plön S (2004) The Status and Natural History of Pygmy (*Kogia Breviceps*) and Dwarf (*K. Sima*) Sperm Whales off Southern Africa. Rhodes University.

Plön S, Cockcroft VG, Froneman WP (2015) The natural history and conservation of Indian ocean humpback dolphins (*Sousa plumbea*) in South African waters. *Adv Mar Biol* 72: 143–162.

Ponce de Leon A (1984) Lactancia y composicion cunatitativa de la leche del lobo fino sudamericano (*Arctocephalus australis* Zimmermann, 1873). Industria Lobera y Pesquera del Estado, Montevideo, Uruguay. *Anales* 1: 43–58.

Ponce de Leon A, Pin OD (2006) Distribución, reproducción y alimentación del lobo fino *Arctocephalus australis* y del león marino Otaria flavescens en Uruguay. In: Menafra R, Rodríguez-Gallego L, Scarabino F, Conde D, eds. Bases Para La Conservación y El Manejo de La Costa Uruguaya. VIDA SILVESTRE URUGUAY, pp 305–314.

Purdie P (1885) Chemical composition of the milk of the porpoise. *Chem News J Phys Sci* 52: 170.

Quakenbush L, Citta J, Crawford J (2009) Biology of the Spotted Seal (*Phoca Largha*) in Alaska from 1962 to 2008. National Marine Fisheries Service.

Quakenbush LT, Citta JJ (2008) Biology of the Ribbon Seal in Alaska. National Marine Fisheries Service.

Ramos RMA, Di Beneditto APM, Lima NRW (2000) Growth parameters of *Pontoporia blainvillei* and *Sotalia fluviatilis* (Cetacea) in northern Rio de Janeiro, Brazil. *Aquat Mamm* 65–75.

Ramos RMA, Di Beneditto APM, Siciliano S, Santos MCO, Zerbini AN, Vicente AFC, Zampirolli E, Alvarenga FS, Fragoso AB, Lailson-Brito J, *et al.* (2010) Morphology of the Guiana dolphin (*Sotalia guianensis*) off southeastern Brazil: growth and geographic variation. *Lat Am J Aquat Mamm* 8: 137–149.

Ramsay MA, Stirling I (1988) Reproductive biology and ecology of female polar bears (*Ursus maritimus*). *J Zool* 214: 601–633.

Rea LD, Costa DP (1992) Changes in standard metabolism during long-term fasting in northern elephant seal pups (*Mirounga angustirostris*). *Physiol Zool* 65: 97–111.

Read AJ (1990) Reproductive seasonality in harbour porpoises, *Phocoena phocoena,* from the Bay of Fundy. *Can J Zool* 68: 284–288.

Read AJ, Hohn AA (1995) Life in the fast lane: the life history of harbor porpoises from the Gulf of Maine. *Mar Mammal Sci* 11: 423–440.

Read AJ, Wells RS, Hohn AA, Scott MD (1993) Patterns of growth in wild bottlenose dolphins, *Tursiops truncatus*. *J Zool* 231: 107–123.

Reddy M, Kamolnick T, Skaar D, Curry C, Ridgway S (1993) Bottlenose dolphins: Energy consumption during pregnancy, lactation, and growth. *Int Mar Mammal Trainers Assoc Annu Conf* 1–37.

Reese CS, Calvin JA, George JC, Tarpley RJ (2001) Estimation of fetal growth and gestation in bowhead whales. *J Am Stat Assoc* 96: 915–923.

Reid JP, Bonde R, O’Shea T (1995) Reproduction and Mortality of Radio-Tagged and Recognizable Manatees on the Atlantic Coast of Florida. Information and Technology Report, U.S. Fish and Wildlife Service.

Reidarson TH, McBain JF, Yochem PK (2001) Medical and nutritional aspects of a rehabilitating California gray whale calf. *Aquat Mamm* 27: 215–221.

Reyes JC, van Waerebeek K (1995) Aspects of the biology of Burmeister’s porpoise from Peru. *Rep - Int Whal Comm Spec Issue*.

Rice DW (1977) Synopsis of biological data on the sei whale and Bryde’s whale in the eastern North Pacific. *Reports Int Whal Comm Spec Issue* 1: 92–97.

Rice DW, Wolman AA (1971) The Life History and Ecology of the Gray Whale (*Eschrichtius Robustus*). American Society of Mammalogists.

Richardson SF, Stenson GB, Hood C (2003) Growth of the harbour porpoise (*Phocoena phocoena*) in eastern Newfoundland, Canada. *NAMMCO Sci Publ* 5: 211.

Ridgway S, Benirschke K (1977) Breeding Dolphins: Present Status, Suggestions for the Future. Proceedings of the 1975 Dolphin Breeding Workshop. San Diego, CA.

Ridgway S, Kamolnick T, Reddy M, Curry C, Tarpley RJ (1995) Orphan-induced lactation in *Tursiops* and analysis of collected milk. *Mar Mammal Sci* 11: 172–182.

Riet-Sapriza FG, Duignan PJ, Chilvers BL, Wilkinson IS, Lopez-Villalobos N, Mackenzie DDS, MacGibbon A, Costa DP, Gales N (2012) Interannual and individual variation in milk composition of New Zealand sea lions (*Phocarctos hookeri*). *J Mammal* 93: 1006–1016.

Robeck TR, Monfort SL, Calle PP, Dunn JL, Jensen E, Boehm JR, Young S, Clark ST (2005) Reproduction, growth and development in captive beluga (*Delphinapterus leucas*). *Zoo Biol* 49: 29–49.

Robeck TR, Schmitt TL, Osborn S (2015) Development of predictive models for determining fetal age-at-length in belugas (*Delphinapterus leucas*) and their application toward in situ and ex situ population management. *Mar Mammal Sci* 31: 591–611.

Robeck TR, Schneyer AL, McBain JF, Dalton LM, Walsh MT, Czekala NM, Kraemer DC (1993) Analysis of urinary immunoreactive steroid metabolites and gonadotropins for characterization of the estrous cycle, breeding period, and seasonal estrous activity of captive killer whales (Orcinus orca). *Zoo Biol* 12: 173–187.

Rogan E, Baker J, Jepson P, Berrow S, Kiely O (1997) A mass stranding of white-sided dolphins (*Lagenorhynchus acutus*) in Ireland: biological and pathological studies. *J Zool* 242: 217–227.

Rosas FCW, Lehti KK (1996) Nutritional and mercury content of milk of the Amazon river dolphin, *Inia geoffrensis*. *Comp Biochem Physiol - A Physiol* 115: 117–119.

Rosas FCW, Marigo J, Laeta M, Rossi-Santos MR (2010) Natural history of dolphins of the genus *Sotalia*. *Lat Am J Aquat Mamm* 8: 57–68.

Rosas FCW, Monteiro-Filho ELA (2002) Reproduction of the estuarine dolphin (*Sotalia guianensis*) on the coast of Paraná, southern Brazil. *J Mammal* 83: 507–515.

Ross G (1979) The Smaller Ceteaceans of the South East Coast of Southern Africa. University of Port Elizabeth.

Ross GJB (1984) The smaller cetaceans of the south east coast of southern Africa. *Ann Cape Prov Museums* 15: 173–410.

Ross GJB, Best PB, Donnelly BG (1975) New records of the pygmy right whale (*Caperea marginata*) from South Africa, with comments on distribution, migration, appearance, and behavior. *J Fish Res Board Canada* 32: 1005–1017.

Sasaki S, Komura H, Hashikata M, Yoshida T, Kanzaki B, Hosono A, Lien-Siang C, Yao C (2004) Composition and characteristics of Ginkgo-toothed beaked whale (*Mesoplodon ginkgodes*) milk. *Milk Sci* 53: 15–18.

Scheibe A (1908) Die Zusammensetzund der Walfischmilch. *Munchener Med Wochenschr* 55: 795–796.

Schmidt-Nielsen S, Frog F (1933) On the composition of the fat from a sample of whale milk. *Kongl Nor Vidensk Selsk Forh* 6: 127–129.

Schwarz LK, Runge MC (2009) Hierarchical Bayesian analysis to incorporate age uncertainty in growth curve analysis and estimates of age from length: Florida manatee (*Trichechus manatus*) carcasses. *Can J Fish Aquat Sci* 66: 1775–1789.

Secchi ER, Fagundes CM, Zerbini AN, Möller LM, Dalla Rosa L (1994) Composição físico-química do leite de duas *Pontoporia blainvillei* capturadas acidentalmente no sul do Brasil. In: Abstracts of the VI Reunião de Trabalho de Especialistas em Mamíferos Aquáticos da América do Sul, 24-28 October 1994, Florianópolis,B.

Senthilkumar K, Kannan K, Sinha RK, Tanabe S, Giesy JP (1999) Bioaccumulation profiles of polychlorinated biphenyl congeners and organochlorine pesticides in Ganges river dolphins. *Environ Toxicol Chem* 18: 1511–1520.

Sergeant D (1962) The biology of the pilot or pothead whale *Globicephala melaena* (Traill) in Newfoundland waters. *Fish Res Board Canada* 132: 1–84.

Sergeant D (1973) Biology of white whales (*Delphinapterus leucas*) in western Hudson Bay. *J Fish Res Board Canada* 30: 1065–1090.

Sergeant D, Ronald K, Boulva J, Berkes F (1978) The recent status of *Monachus monachus*, the Mediterranean monk seal. *Biol Conserv* 259–287.

Sergeant DE, St. Aubin DJ, Geraci J (1980) Life history and northwest Atlantic status of the Atlantic white-sided dolphin, *Lagenorhynchus acutus*. *Cetology* 37: 1–12.

Shaughnessy PD, Jones R, Viggers K (2019) On the size of crabeater seal, *Lobodon carcinophaga*, pups. *Mar Mammal Sci* 35: 1653–1658.

Shaughnessy PD, Kerry KR (1989) Crabeater seals *Lobodon carcinophagus* during the breeding season: observations on five groups near Enderby Land, Antarctica. *Mar Mammal Sci* 5: 68–77.

Shirakihara M, Takemura A, Shirakihara K (1993) Age, growth, and reproduction of the finless porpoise, *Neophocaena hocaenoides*, in the coastal waters of Western Kyushu, Japan. *Mar Mammal Sci* 9: 392–406.

Siciliano S, Ramos RMA, Di Beneditto APM, Santos MCO, Fragoso AB, Brito JL, Azevedo AF, Vicente AFC, Zampirolli E, Alvarenga FS, *et al.* (2007) Age and growth of some delphinids in south-eastern Brazil. *J Mar Biol Assoc United Kingdom* 87: 293–303.

Siversten E (1941) On the biology of the harp seal, *Phoca groenlandica*. *Hvalrad Skr* 26: 1–164.

Skinner J, Klages N (1994) On some aspects of the biology of the Ross seal *Ommatophoca rossii* from King Haakon VII Sea, Antarctica. *Polar Biol* 14: 467–472.

Slooten E (1991) Age, growth, and reproduction in Hector’s dolphins. *Can J Zool* 69: 1689–1700.

Smith RJ, Read AJ (1992) Consumption of euphausiids by harbour porpoise (*Phocoena phocoena*) calves in the Bay of Fundy. *Can J Zool* 70: 1629–1632.

Sørensen TB, Kinze CC (1994) Reproduction and reproductive seasonality in Danish harbour porpoises, *Phocoena phocoena*. *Ophelia* 39: 159–176.

Southwell C, Kerry K, Ensor P, Woehler EJ, Rogers T (2003) The timing of pupping by pack-ice seals in East Antarctica. *Polar Biol* 26: 648–652.

Stacey PJ, Arnold PW (1999) *Orcaella brevirostris*. *Mamm Species* 1: 1–8.

Stacey PJ, Leatherwood S (1997) The Irrawaddy dolphin, *Orcaella brevirostris*: A summary of current knowledge and recommendtations for conservation action. *Asian Mar Biol* 14: 195–214.

Stevick PT (1999) Age-length relationships in humpback whales: A comparison of strandings in the western North Atlantic with commercial catches. *Mar Mammal Sci* 15: 725–737.

Stewart REA, Lavigne DM (1980) Neonatal growth of northwest Atlantic harp seals, *Phoca groenlandica*. *J Mammal* 61: 670–680.

Stewart REA, Stewart BE, Lavigne DM, Miller GW (1989) Fetal growth of Northwest Atlantic harp seals, *Phoca groenlandica*. *Can J Zool* 67: 2147–2157.

Struntz DJ, Mclellan WA, Dillaman RM, Blum JE, Kucklick JR, Pabst DA (2004) Blubber development in bottlenose dolphins (*Tursiops truncatus*). *J Morphol* 259: 7–20.

Subramanian A, Tanabe S, Tatsukawa R (1988) Estimating some biological parameters of Baird’s beaked whales using PCBs and DDE as tracers. *Mar Pollut Bull* 19: 284–287.

Sumich JL, Blokhin SA, Tiupeleyev PA (2013) Revised estimates of foetal and post-natal growth in young gray whales (*Eschrichtius robustus*). *J Cetacean Res Manag* 13: 89–96.

Suydam RS (2009) Age, Growth, Reproduction, and Movements of Beluga Whales (*Delphinapterus Leucas*) from the Eastern Chukchi Sea. University of Washington.

Symons H, Weston R (1958) Studies on the humpback whale (*Megaptera nodosa*) in the Bellingshausen Sea. *Nor Hvalfangst-Tid* 47: 53–81.

Takata M (1921) Untersuchugen uber Cetacea. II. Uber die Milch des Finnwals. *Tohoku J Exp Med* 2: 344–354.

Takemura K (1927) Untersuchungen uber Cetacea. XXVII. Uber die Milch verschiedener Walarten. *Japanese J Med Sci II Biochem* 1: 131–134.

Tas’an, Irwandy A, Sumitro M, Hendrokusumo S (1980) *Orcaella brevirostris* (Gray, 1866) from Mahakam River. *Jaya Ancol Ocean Jakarta* 60.

Tavolga M, Essapian E (1957) The behavior of the bottle-nosed dolphin (*Tursiops truncatus*): mating, pregnancy, parturition and mother-infant behavior. *Zoologica* 42: 11–31.

Tedman R, Green B (1987) Water and sodium fluxes and lactational energetics in suckling pups of Weddell seals (*Leptonychotes weddellii*). *J Zool* 212: 29–42.

Thomas J, DeMaster D, Stone S, Andriashek D (1980) Observations of a newborn Ross seal pup (*Ommatophoca rossi*) near the Antarctic Peninsula. *Can J Zool* 58: 2156–2158.

Thomas J, Pastukhov V, Elsner R, Petrov E (1982) *Phoca sibirica*. *Mamm Species* 1148–1148.

Thomas PO, Taber SM (1984) Mother-infant interaction and behavioral development in southern right whales, *Eubalaena australis*. *Behaviour* 88: 42–60.

Tikhomirov E (1968) Body growth and development of reproductive organs of the North Pacific phocids. In: Arsen’ev V, Panin K, eds. Pinnipeds of the North Pacific. Pischevaya Promyshlennost (Food Industry), Moscow, Russia. (Translated from Russian by J. Salkind, Isreal Program for Science Translations, Jersualem, Israel, 29 p)., pp 213–241.

Tinker MT, Tomoleoni, Joseph A. Weitzman BP, Staedler M, Jessup D, Murray MJ, Miller M, Burgess T, Bowen L, Miles AK, Thometz N, *et al.* (2019) Southern sea otter (*Enhydra lutris nereis*) population biology at Big Sur and Monterey, California: Investigating the consequences of resource abundance and anthropogenic stressors for sea otter recovery. U.S. Geological Survey Open-File Report 2019.

Tomilin AG (1957) SSSR I Prilezhashchikn Stran (The Mammals of the USSR and Adjacent Countries). Vol IX. Kitoobraznye (Cetacea). Publ. Acad. Nauk USSR, Moscow.

Tønnesen P, Gero S, Ladegaard M, Johnson M, Madsen PT (2018) First-year sperm whale calves echolocate and perform long, deep dives. *Behav Ecol Sociobiol* 72. doi:10.1007/s00265-018-2570-y

Tormosov DD, Mikhaliev YA, Best PB, Zemsky VA, Sekiguchi K, Brownell RL (1998) Soviet catches of southern right whales *Eubalaena australis*, 1951-1971. Biological data and conservation implications. *Biol Conserv* 86: 185–197.

Trillmich F (1986) Attendance behavior of Galápagos fur seals. In: Gentry RL, Kooyman GL, eds. Fur Seals: Maternal Strategies on Land and at Sea. Princeton University Press, pp 168–185.

Trillmich F, Lechner E (1986) Milk of the Galapagos fur seal and sea lion, with a comparison of the milk of Eared seals (Otariidae). *J Zool* 209: 271–277.

Trillmich F, Wolf JBW (2008) Parent – offspring and sibling conflict in Galápagos fur seals and sea lions. *Behav Ecol Sociobiol* 62: 363–375.

Trites AW (1991) Fetal growth of northern fur seals: life-history strategy and sources of variation. *Can J Zool* 69: 2608–2617.

Trites AW, Bigg MA (1996) Physical growth of northern fur seals (*Callorhinus ursinus*): seasonal fluctuations and migratory influences. *J Zool Soc London* 238: 459–482.

Ullrey DE, Schwartz CC, Whetter PA, Rajeshwar Rao T, Euber JR, Cheng SG, Brunner JR (1984) Blue-Green color and composition of Stejneger’s beaked whale (*Mesoplodon stejnegeri*) milk. *Comp Biochem Physiol -- Part B Biochem* 79: 349–352.

Valqui J (2012) The marine otter *Lontra felina* (Molina, 1782): A review of its present status and implications for future conservation. *Mamm Biol* 77: 75–83.

van Waerebeek K, Read AJ (1994) Reproduction of dusky dolphins, *Lagenorhynchus obscurus*, from coastal Peru. *J Mammal* 75: 1054–1062.

Vaz-Ferreria R, Ponce de Leon A (1987) South American fur seal, *Arctocephalus australis*, in Uruguay. In: Croxall JP, Gentry RL, eds. Status, Biology, And Ecology of Fur Seals. Proceedings of an International Symposium and Workshop.

Vaz Ferreira R (1982) *Otaria flavescens* (Shaw), South American sea lion. *FAO Mamm Seas IV* 477–495.

Venuto R, Botta S, Barreto AS, Secchi ER, Fruet PF (2020) Age structure of strandings and growth of Lahille’s bottlenose dolphin (*Tursiops truncatus gephyreus*). *Mar Mammal Sci* 36: 813–827.

Vergara-Parente JE, Parente CL, Marmontel M, Silva JCR, Sá FB (2010) Growth curve of free-ranging *Trichechus inunguis*. *Biota Neotrop* 10: 89–92.

Vos DJ, Shelden KEW, Friday NA, Mahoney BA (2020) Age and growth analyses for the endangered belugas in Cook Inlet, Alaska. *Mar Mammal Sci* 36: 293–304.

Wahrenbrock EA, Maruschak GF, Elsner R, Kenney DW (1974) Respiration and metabolism in two baleen whale calves. *Mar Fish Rev* 36: 3–8.

Webster T, Dawson S, Slooten E (2010) A simple laser photogrammetry technique for measuring Hector’s dolphins (*Cephalorhynchus hectori*) in the field. *Mar Mammal Sci* 26: 296–308.

Weller DW, Würsig B, Bradford AL, Burdin AM, Blokhin SA, Minakuchi H, Brownell RL (1999) Gray whales (*Eschrichtius robustus*) off Sakhalin Island, Russia: Seasonal and annual patterns of occurrence. *Mar Mammal Sci* 15: 1208–1227.

Werner R (1996) Composition and energy content of milk from southern sea lions (*Otaria flavescens*). *Mar Mammal Sci* 12: 313–317.

West KL (2002) Ecology and Biology of the Rough-Toothed Dolphin (*Steno Bredanensis*). *Dissertation*. University of Hawaii at Manoa.

West KL, Oftedal OT, Carpenter JR, Krames B., Campbell M, Sweeney J. (2007) Effect of lactation stage and concurrent pregnancy on milk composition in the bottlenose dolphin. *J Zool* 273: 148–160.

Wheatley KE, Bradshaw CJA, Davis LS, Harcourt RG, Hindell MA (2006) Influence of maternal mass and condition on energy transfer in Weddell seals. *J Anim Ecol* 75: 724–33.

White J (1953) Composition of whales’ milk. *Nature* 171: 612.

Wiig Ø (1985) Morphometric variation in the hooded seal *Cystophora cristata*. *J Zool* 206: 497–508.

Wilson SC, Dolgova E, Trukhanova I, Dmitrieva L, Crawford I, Baimukanov M, Goodman SJ (2016) Breeding behavior and pup development of the Caspian seal, *Pusa caspica*. *J Mammal* 98: 143–153.

Winship A, Trites A, Rosen D (2002) A bioenergetic model for estimating the food requirements of Steller sea lions *Eumetopias jubatus* in Alaska, USA. *Mar Ecol Prog Ser* 229: 291–312.

Winship AJ, Trites AW, Calkins DG (2001) Growth in body size of Steller sea lion (*Eumetopias jubatus*). *J Mammal* 82: 500–519.

Xian Y, Wang K, Xiao J, Wang D (2012) Suckling behavior and its development in two Yangtze finless porpoise calves in captivity. *Zoo Biol* 31: 229–234.

Yablokov A, Bel’kovich B, Borisov V (1974) Whales and Dolphins Part II. Joint Publications Research Service 62150-2.

Yunker GB, Hammill MO, Gosselin JF, Dion DM, Schreer JF (2005) Foetal growth in north-west Atlantic grey seals (*Halichoerus grypus*). *J Zool* 265: 411–419.

Zeng Q, Wang X, Zhu Q (2021) Preliminary study on the reproductive ecology of a threatened Indo-Pacific humpback dolphin (*Sousa chinensis*) population in Xiamen Bay, China. *Aquat Mamm* 47: 43–52.

Zeng X, Huang SL, Qian Z, Hao Y, Wang D, Ji J, Nabi G (2017) Characterization of milk composition in narrow-ridged finless porpoises (*Neophocaena asiaeorientalis*) at different lactation stages. *Mar Mammal Sci* 33: 803–816.

Zenkovich B (1938) Milk of large-sized cetaceans. *Comptes Rendus l’Academie des Sci l’URSS* 20.

Zhang PJ, Song XR, Han JB, Wang LM, Yang Y (2014) Milk composition, milk consumption, and growth rate of a captive spotted seal (*Phoca largha*) pup from Liaodong Bay, China. *Can J Zool* 92: 449–452.

Zhou K (2008) Baiji *Lipotes vexillifer*. In: Wursig B, Perrin WF, Thewissen J, eds. Encyclopedia of Marine Mammals. Elsevier Science & Technology, pp 71–76.
